# The Origin of Prebiotic Information System in the Peptide/RNA World: A Simulation Model of the Evolution of Translation and the Genetic Code

**DOI:** 10.3390/life9010025

**Published:** 2019-03-01

**Authors:** Sankar Chatterjee, Surya Yadav

**Affiliations:** 1 Department of Geosciences, Museum of Texas Tech University, Box 43191, 3301 4th Street, Lubbock, TX 79409, USA; 2 Rawls College of Business, Texas Tech University, Box 42101, 703 Flint Avenue, Lubbock, TX 79409, USA; surya.yadav@ttu.edu

**Keywords:** peptide/RNA world, prebiotic information system, translation and the genetic code, bridge peptide and aaRS, ribozyme and tRNA, tRNA and mRNA, coevolution of translation machine and the genetic code, MVC architecture pattern and biological information, numerical codons, AnyLogic software for computer simulation of translation machine

## Abstract

Information is the currency of life, but the origin of prebiotic information remains a mystery. We propose transitional pathways from the cosmic building blocks of life to the complex prebiotic organic chemistry that led to the origin of information systems. The prebiotic information system, specifically the genetic code, is segregated, linear, and digital, and it appeared before the emergence of DNA. In the peptide/RNA world, lipid membranes randomly encapsulated amino acids, RNA, and peptide molecules, which are drawn from the prebiotic soup, to initiate a molecular symbiosis inside the protocells. This endosymbiosis led to the hierarchical emergence of several requisite components of the translation machine: transfer RNAs (tRNAs), aminoacyl-tRNA synthetase (aaRS), messenger RNAs (mRNAs), ribosomes, and various enzymes. When assembled in the right order, the translation machine created proteins, a process that transferred information from mRNAs to assemble amino acids into polypeptide chains. This was the beginning of the prebiotic *information* age. The origin of the genetic code is enigmatic; herein, we propose an evolutionary explanation: the demand for a wide range of protein enzymes over peptides in the prebiotic reactions was the main selective pressure for the origin of information-directed protein synthesis. The molecular basis of the genetic code manifests itself in the interaction of aaRS and their cognate tRNAs. In the beginning, aminoacylated ribozymes used amino acids as a cofactor with the help of bridge peptides as a process for selection between amino acids and their cognate codons/anticodons. This process selects amino acids and RNA species for the next steps. The ribozymes would give rise to pre-tRNA and the bridge peptides to pre-aaRS. Later, variants would appear and evolution would produce different but specific aaRS-tRNA-amino acid combinations. Pre-tRNA designed and built pre-mRNA for the storage of information regarding its cognate amino acid. Each pre-mRNA strand became the storage device for the genetic information that encoded the amino acid sequences in triplet nucleotides. As information appeared in the digital languages of the codon within pre-mRNA and mRNA, and the genetic code for protein synthesis evolved, the prebiotic chemistry then became more organized and directional with the emergence of the translation and genetic code. The genetic code developed in three stages that are coincident with the refinement of the translation machines: the GNC code that was developed by the pre-tRNA/pre-aaRS /pre-mRNA machine, SNS code by the tRNA/aaRS/mRNA machine, and finally the universal genetic code by the tRNA/aaRS/mRNA/ribosome machine. We suggest the coevolution of translation machines and the genetic code. The emergence of the translation machines was the beginning of the Darwinian evolution, an interplay between information and its supporting structure. Our hypothesis provides the logical and incremental steps for the origin of the programmed protein synthesis. In order to better understand the prebiotic information system, we converted letter codons into numerical codons in the Universal Genetic Code Table. We have developed a software, called CATI (**C**odon-**A**mino Acid-**T**ranslator-**I**mitator), to translate randomly chosen numerical codons into corresponding amino acids and vice versa. This conversion has granted us insight into how the genetic code might have evolved in the peptide/RNA world. There is great potential in the application of numerical codons to bioinformatics, such as barcoding, DNA mining, or DNA fingerprinting. We constructed the likely biochemical pathways for the origin of translation and the genetic code using the Model-View-Controller (MVC) software framework, and the translation machinery step-by-step. While using AnyLogic software, we were able to simulate and visualize the entire evolution of the translation machines, amino acids, and the genetic code.

## 1. Introduction

The origin of life on early Earth remains one of the deepest mysteries in modern science. Recent evidence suggests that life may have emerged about four billion years ago through the spontaneous interaction of biomolecules in steaming hydrothermal environments, but the actual pathways of biogenesis are still shrouded in mystery [1]. Life’s first building blocks had their origin in the tiny ice granules of interstellar space and they can be found on carbonaceous chondrites, comets, and the Murchison meteorite [2,3,4]. Asteroids were continuously battering the Hadean Earth [5]. As a result, thousands of craters probably pocked the surface of the Eoarchean crust, like the surface of the Moon and Mercury. Unlike our planetary neighbors, however, the crater basins of Eoarchean Earth filled with water and biomolecules, and developed a complex network of hydrothermal systems [6]. Carbonaceous chondrites delivered both water and the building blocks of life to the planetary surface, thus creating innumerable crater basins [7]. The meteorite collisions that created hydrothermal crater lakes in the Eoarchean crust filled with water, organic molecules, and various hydrothermal fluids, gases, and energy; inadvertently, these became the perfect crucibles for prebiotic chemistry [6,7,8,9,10,11,12,13]. There is now evidence that the Late Heavy Bombardment impact spike (4.1–3.8 Ga) during the Hadean–Eoarchean interval may not have happened; most likely, there was a continuous decrease of the bolide flux during this interval [14]. Minerals, such as zircons, and water-lain sediments in the ancient Hadean/Archean crust indicate that liquid water was prevalent as early as four billion years ago. Earth was no longer an alien inhospitable world, but it was transforming into a life-supporting environment [15].

The early atmosphere of Eoarchean Earth was dominated by CO_2_ and N_2_, not by CH_4_ and NH_3_. Moreover, the main source of carbon on the primitive Earth was atmospheric CO_2_, which might have contributed to the formation of many organic compounds [16]. In the hydrothermal crater lake, cosmic and terrestrial chemicals were mixed, concentrated, and linked together by convective currents in these sequestered crater lakes, which were powered by hydrothermal, solar, tidal, and chemical energies; here, life began to brew [6,7,8,9,10,11,12,13]. Both the chemicals and the energy that were found in these hydrothermal crater lakes fueled most of the chemical reactions that are necessary for prebiotic synthesis and the resulting emergence of life [13]. Monomers, such as nucleotides and amino acids, were selected from random assemblies of molecular pools and then polymerized on the pores and pockets of the mineral substrate to create RNAs and peptides, heralding the peptide/RNA world [9,17,18,19,20,21,22,23,24,25,26]. Most likely, pores and crevices of the mineral substrate of the crater floor acted as receptacles for concentrations of simple RNA and peptide molecules [21,22]. The establishment of a symbiotic relationship between peptides and RNA was a landmark threshold in the evolution of life. These two biopolymers, with distinct structures and functions, became codependent and partner. The prebiotic peptides functioned as stabilizing and catalytic agents in the chemical reactions and they adapted to the high temperature vent environment.

The ability of the lipid membranes to encapsulate various monomers and biopolymers was crucial in terms of efficiency, stability, and molecular symbiosis. Encapsulation further ensured the concentration and protection of life-encouraging ingredients from the vent environment, thus enhancing further biosynthesis [1,9,22]. Molecular symbiosis among membranes, RNAs, amino acids, and peptides was the driving force for the origin of complex cellular components. Lipid membranes were randomly encapsulated RNA and peptide molecules from the mineral substrates of the crater floor to initiate a molecular symbiosis inside the protocells that led to the hierarchical emergence of several cell components and their functionalities: first plasma membranes, then peptides and RNAs, then transfer RNAs, messenger RNAs, and then ribosomes; these cooperative molecules created the prebiotic information system step-by-step for programmed protein synthesis [9].

Three classes of RNA molecules, messenger RNAs (mRNA), transfer RNAs (tRNA), and rRNA were the prime players in the expression of genetic information: mRNA was the initial storage molecule of genetic information, tRNA was the carrier of specific amino acids, and rRNA was the essential constituent of the protein producing ribosomes. The interactions between diverse RNA molecules and the myriad of amino acids and enzymes led to the gradual evolution of translation and the genetic code [27]. The peptide/RNA partnership performed two major functions during the origin of translation: storing information and stabilizing and catalyzing chemical reaction. As these two molecules began to develop in concert, the mRNA specified, in triplet code, the amino acid sequence of proteins. RNA molecules and amino acids began to communicate in different languages via bilingual enzymes that allowed for biomolecules to cooperate with each other, leading to information systems and translation. The key processes of the information flow from mRNA to proteins emerged during this stage. As information was stored in the symbolic languages of nucleotides and amino acids, biosynthesis became less random and more organized and directional. With the advent of DNA, genetic information began to flow from DNA, to mRNA, to protein by a two-step process: transcription and translation [27,28].

Recently, it has been argued that the genetic software provides a singular definition regarding what life is [29]. In this view, life emerged in that instant when information gained control over the biomolecules. The information-directed protein synthesis is a unique signature of life. Biological information separates life from nonlife. Although it is difficult to define what makes life so distinctive and remarkable, there is a general agreement that its informational aspect is a key property, perhaps being the key property [30].

We agree with the view of an algorithmic origin of life that indicates a complex system that is comprised of informational networks [31]. However, we suggest that life is more sophisticated than any man-made computer system where the software/hardware dichotomy is blurred and integrated. We find that this computer analogy too simplistic. Both the informational and functional biopolymers in the translational machinery can be viewed as highly mobile molecular nanobots, which are fully equipped with both the information and the material that are needed to accomplish their tasks. These nanobots ‘know’ how to put themselves together by self-assembly or by cooperation with other molecules. It is our proposition that these complex molecular characteristics of life actually appeared before first life. These molecular nanobots are complex, self-replicating, and self-managing information systems in themselves, being analogous to the ‘Universal Constructor’ (UC) conceived by von Neumann [32].

To begin to understand how nature invented highly complex and specialized information systems from the vast array of disparate possibilities, we began this quest by running computer simulations of the major biosynthetic steps that might help to explain the emergence of the system. In this paper, we use the Model-View-Controller (MVC) architecture [33] to reconstruct the molecular translation machinery; we built our model from the components that are known to have existed in the prebiotic environment, such as amino acids, nucleotides, and various peptides. In information systems, the MVC architectural pattern has been used for consolidating information together, processing it into a model, isolating it from its manipulation (controller), and then presenting the component (the view) that determines the output form of the product (the artifact). The major premise of the pattern is the modularity and distribution of processing. MVC separates the three different aspects of information processing: the data (the model), the visual representation of the data (the view), and the interface between the view and the model (the controller). The purpose of this article is to review the latest views on the origin of an information system in the prebiotic world during the emergence of translation and the genetic code.

## 2. Peptide/RNA World

Among several competing hypotheses regarding how life arose on early Earth, the ‘RNA world’ model is widely accepted [27,28,34,35,36,37,38,39]. The RNA world has become the main paradigm in the current origin of life research in which RNA assumed informational and functional roles. RNA molecules, such as ribozymes, can act as catalysts for chemical reactions between other RNA molecules. The discovery of catalytic RNAs and the revelation that the ribosome is, in fact, a ribozyme, together added strong circumstantial evidence for the RNA world theory [39].

Despite the conceptual elegance of the RNA world, this hypothesis faces formidable difficulties, primarily the immense challenge of RNA synthesis under plausible prebiotic conditions [22,40,41]. Various building blocks of RNA molecules, such as sugar, phosphorous, and the purine and pyrimidine nucleobases have been identified in carbonaceous chondrites, comets, and interplanetary dust particles [2,3]. During the polymerization of activated nucleotides on the surface of the clay substrates to from primitive RNA molecules, a steady input of peptides was essential [22]. Conversely, amino acids could be easily polymerized on the mineral surface to form peptide molecules [42]. The RNA molecule is inherently fragile in the natural environment and constantly degrades into smaller fragments through hydrolysis, preventing the faithful reproduction. Peptides provided stability to RNA molecules.

The RNA world might have existed, but the exclusivity of RNA and the neglect of peptide and lipid membrane could have been overstated. Vent environments that could support RNA synthesis no doubt also spawned many other organic compounds. It is irrational to think that vent environments exclusively created a load of nucleotides or RNA. Amino acids are easier to synthesize than RNA, as the Miller-type experiment suggests. The versatility of RNA molecules does not prevent the formation of peptides concurrently in the vent environments, especially when peptides were the likely outcome in prebiotic synthesis [9]. Peptides were easy to synthesize than RNAs in the primordial environment. Moreover, amino acids were probably among the most abundant biogenetic building blocks available on both the prebiotic Earth and meteorites [2,4]. There is a growing consensus that RNAs and peptides simultaneously appeared during prebiotic synthesis. The primordial vent environment that could support RNA synthesis no doubt also created many other organic compounds—for example, peptides—and lipid-like membranes, which are much less chemically challenging to generate [43]. In recent times, the RNA world paradigm is shifting to a peptide/RNA world paradigm [9,17,18,19,20,21,22,23,24,25,26].

There is increasing evidence that RNA and peptide molecules interacted very early on in the origin of the genetic code (rather than RNA and RNA worlds giving rise to proteins), even short peptides had significant catalytic capabilities. Recent experiment suggests that ribozyme recruits an assortment of proteins to the RNA world as it evolves [44]. Ribozyme, such as RNase P, recognizes pre-tRNA and processes to generate mature tRNAs in collaboration with an assemblage of proteins, thus favoring peptide/RNA world in the early stage of RNA evolution. RNA and protein, two complementary molecules that exist in the prebiotic environment, mingled and interacted to form a dynamic system; one cannot exist without the other. Given that life depends on a diversity of molecule types in a symbiotic effort, each interacting with the other in complicated ways, it is hard to imagine that it would have started with just a single type of molecule.

The duality of replication and metabolism is the intrinsic property of life and it must have simultaneously appeared before the origin of the DNA [45]. RNAs provide instructions to build proteins with the help of various enzymes. The establishment of symbiotic relationships between peptides and RNAs was a fundamental threshold in the evolution of life. These two biopolymers, with distinct structures and functions, became codependent. RNA and peptides worked in tandem to expand their informational, structural, and functional repertoires. The fundamental property of life, replication, and metabolism is believed to have evolved in the peptide/RNA world, where RNA stores genetic information and peptide enzymes function as catalysts [45]. The direct evolution of inherited genetic information coupled to encoded functional proteins, as is observed in real-world molecular biology, is far more plausible than any scenario in which there was, as initial RNA, a world of ribozymes sophisticated enough to operate a genetic code [19].

## 3. The Age of Information

The age of information arose as an emergent property in the peptide/RNA world before the origin of DNA. The informational and functional molecules, such as RNAs and peptides, have a high degree of specific complexity. The age of *information* introduces molecular communication and complementarity—the lock-and-key relationship—between RNA and peptides. The base pairing of RNA and its replication played a crucial role in building the information system. Different species of RNA would evolve to specify different functions in the information system.

The genetic information is essentially a digital data, plus meaning [46]. The base sequences of mRNA provide the data and the meaning is the translation of the data into a functional protein. Translation is transferring information from the language of mRNA to the language of proteins [27,28]. During translation, mRNAs serve as a data-storage system, transmitting digital instruction to molecular machines, the ribosomes, which manufacture protein molecules. RNAs are essential in encoding information. Three kinds of RNA molecules play major roles in translation: The messenger RNAs (mRNAs) carry genetic information to the ribosomes where the proteins are synthesized. The transfer RNAs (tRNAs) function as adaptors between amino acids and the codons in mRNA during translation. The tRNAs also carry the specific amino acids to the ribosome during protein synthesis; they are the handler by which the mRNA is pulled through the ribosome via-codon anticodon interactions in the course of translocation. The ribosomal RNAs (rRNAs) are the structural and catalytic components of the ribosomes. Arguably, the ribosomes are the most intricate and sophisticated nanomachines in nature that translate the nucleotide sequences of mRNAs into amino acid-sequences of proteins.

The RNA-based information system mostly depends on enzymic peptides for the replication and translation of the nucleic acids. However, the specificity of the enzyme depends on their amino acid sequences, which are determined by the sequences of nucleotides in RNAs. In the beginning, the amino acids were utilized by ribozymes as cofactors, developing complex interactions between different RNAs and amino acids that led to the origin of translation and genetic code. Several enzymes were essential for protein synthesis, including ribozymes, peptides, peptidyl transferase, and aminoacyl-tRNA synthetase (aaRS).

## 4. The Use of Information Theory in Biology

The discovery of genetic encoding of the DNA molecule, and its mode of translation into protein structures, secured the modern view of biology as an information science [30,31,32]. Biological systems have embedded information structure for supporting their functions [47,48,49,50]. An information system can be defined as a set of related components that work together for storing and processing data and for providing information [51]. This definition of an information system views it as an open system. Like an open system, an information system has a purpose and it interacts with its environment. It differentiates and elaborates itself in dealing with the changing environmental conditions just like biological systems. Terms, such as ‘automata’ and ‘machine’, refer to a form of an information system.

**Figure 1:**
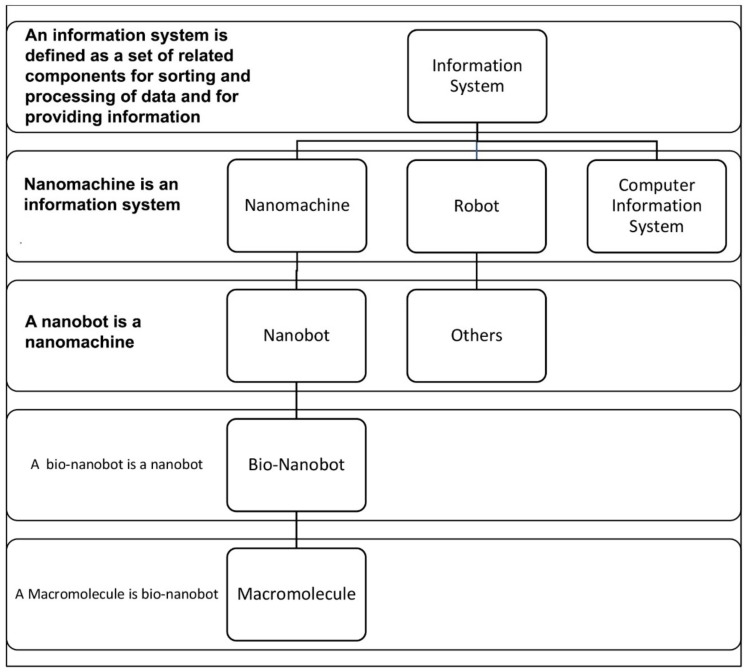
A hierarchy of Information Systems and Nanobots. This diagram shows a unified definition of various terms used for molecular systems. It related the idea of an information system with terms like ‘nanomachines’, etc.

Even though the ideas of artificial automata came from the observation and study of natural automata, such as biological systems, we tend to use man-made machines and other artifacts as a metaphor to better understand the biological systems. Various metaphors, such as nanobot, bio-nanobot, etc. have been used in the literature to refer to different types of information system. Metaphors are useful because they are efficient: they transfer a complex meaning in a few words. Some of the popular metaphors, including robot and nanomachine, are deeply entrenched in our social life, news, and literature. Figure 1 relates these terms to the biological information system. Biological systems exhibit characteristics that relate to processing and using messages that convey information.

We use metaphors, such as nanobots and computers, for information systems in cells. This practice may be fine as long as we understand the limitations of these metaphors. It’s obvious that a cell is more complex than a computer system. The metaphors and analogies only explain a portion of the activities of the biological systems. We believe that, in most cases, a basic-level metaphor is more useful and discriminatory in explaining difficult concepts by association than a higher-level or system-level metaphor. For example, to say ‘a cell is a nanobot’ is not very revealing about the complexity of a cell. However, it is more meaningful if we say that a cell is a combination of assembler, transcriptor, translator, adapter, pattern-recognizer, pattern-copier, builder, inventory of materials, etc. Metaphors, like assembler, translator, adapter, etc., can be called as the basic-level metaphors. Taken together, they reveal closely the functions and structure of a cell. Another example, a ribosome can be metaphorically described as an assembler that assembles a protein with the help of charged tRNAs. This also points out the fact that a ribosome is part of a cell. It also illustrates the hierarchical nature of relationships between biological systems and between metaphors. To our knowledge, there is no higher-level metaphor that adequately describes a cell or, for that matter, any other biological system. A basic-level metaphor or a combination of basic-level metaphors can better describe a biological system. It is important to note that any of these basic-level metaphors can be viewed (modeled) as an information system.

Many fundamental biological processes involve the flow of information. The potential for new biological knowledge arises from investigating the complex interaction of many different levels of biological information from DNA to mRNA to protein to cells to organs to individuals. All macromolecules, organelles and cells, no matter how rudimentary, use information and material to conduct their tasks. Information that is used by them is in various forms such as attractiveness, proximity, pattern, match, symmetry, sequence, rule, and feedback, etc. These informational terms have the usual meanings. Attractiveness between modules relate to various chemical bonds that form easily between them. Proximity refers to the closeness between molecules. A pattern is a configuration of things in a certain way. Match involves the similarity and complementarity between molecular elements and surfaces. Symmetry relates to the shape of molecules and organisms. A sequence is a specific order in which related things follow each other. A biological sequence is a molecule that includes smaller molecules, such as nucleotides in RNA or amino acids in proteins. Rules specify conditional information. Feedbacks are information in the form of signals. By the time of DNA-mRNA-Protein synthesis, the cells had developed a very advanced, stable, and streamlined biological information system to help carry out the translation. Our discussion here is limited to the emergence of the information system in the peptide/RNA world, before the appearance of DNA and the first cells.

The characteristics of biological systems were identified and recognized by early pioneers in information systems. They have made tremendous contributions to our understanding of the biological processes by envisioning and proposing the concepts, frameworks, and models that help to imitate the biological processes. Von Neumann [32] proposed the idea of natural and artificial automata and developed detailed models to emulate the behavior and actions of natural automata. Turing [52,53] was instrumental in recognizing organized shapes, patterns, forms, and decision making in biological organisms. Shannon [54] formalized the concepts of information as a message, the transmission of message, and the semantic aspect of communication and information. The Shannon equation is practical for characterizing a signal (or message) and estimating the physical space that it may occupy; most random sequences give the highest possible entropy value (bits). Shannon’s information entropy (H) is often confused with the physical entropy (S), because both concepts have a very similar mathematical formulation, but different meanings. Thermodynamic entropy characterizes a statistical ensemble of molecular states, while Shannon’s entropy characterizes a statistical ensemble of messages [55,56]. For Shannon, information can be defined through entropy as a discrete set of probabilities to a receiver that reduces uncertainties. In biology, there is another dimensional aspect: information has both a probabilistic and linguistic context over an observable data set. Information in a biological context must exist within ‘meaning’ [46]. Genetically encoded biological information appears to be somewhat different from Shannon entropy.

Wiener [57] enunciated the concepts of control and feedback in systems. Bertlanffy’s general systems theory [58] suggests that all the systems share some common organizing principles. All of these pioneering works in the form of theory, framework, and model have given rise to many advances in technology and biological knowledge. These advances have allowed us to develop better methods to design information systems for the simulation and visualization of biological information systems.

### Evolution of Biological Information System

Life may be defined operationally as an information processing system—a structural hierarchy of various functional units—that has acquired through evolution, the ability to store and process the *information* that is necessary for its own accurate reproduction. Here, it is very useful to take a wider meaning of the word ‘information’ as opposed to just the classical definition of information based upon the information theory [31,52]. There are various definitions of the word information over the years. Historically, the word information has represented three different types of meanings [59]:

(1) information as the process of being informed,
(2) information as a state of an object, and
(3) information as the disposition to inform.

Information as a process includes the ideas of message communication, meaning, and error due to a noisy channel [54]; information as a state of an object covers the idea of knowledge; and, information as the disposition to inform includes the ideas regarding the capacity of an object to inform another object and information as a specific thing [60].

Information can be signals, natural patterns (including shape, space, size, etc.), match, proximity, attractiveness (i.e., hydrophobicity and hydrophilicity), symmetry, sequence, rules, feedback, instructions, algorithms, content, and knowledge, etc. [60,61,62,63]. Biological information involves all of the above types of information.

In this paper, we first reconstruct the plausible biochemical pathways in the prebiotic world for the origin of the translation machines and the genetic code. Later, we apply a biological information system to simulate the origin of translation and the genetic code using different stages of translation machines.

## 5. Temporal Order of Emergence of the Translation Machines

The molecular translation machine consists of various parts and accessories, such as ribozymes, amino acids, tRNAs, aaRS, mRNAs, ribozymes, peptides, and various enzymes. In the modern translation machine, mRNA is decoded in a ribosome to produce a specific amino acid chain or polypeptide. The polypeptide then folds into an active protein and performs its function in the cell. The list of parts of translation machine is not sufficient condition for understanding its biologic function, such as programmed protein synthesis. Understanding how the parts work in unison is also important. However, it is not enough. We have to do reverse engineering to reconstruct how these parts might have evolved and interacted in the prebiotic environment. The origin and evolution of the translation machine may shed new light on how the information system emerged in the peptide/RNA world.

We have now some idea about the molecular milieu in the prebiotic environment in which the genetic code originated in the peptide/RNA world. The prebiotic soup was a rich collection of biomolecules in a highly reactive environment, owing to the constant input of hydrothermal energy. Some of these biomolecules were selected and encapsulated in protocells. The origin of the translation system is the central and the most difficult problem in the study of the origin of life [27,28,64,65,66,67,68]. All of the hypotheses for the origin of the genetic code incorporate peptides as the stabilizing factor in prebiotic reaction [17,18,19,20,21,23,24]. We propose that the transition from peptide to protein in the peptide/RNA world might have given rise to the translation system and the genetic code. This remarkable development could have incrementally occurred by natural selection as the demand for more and more efficient and specific protein enzymes became greater than the supply for protocellular functions. The solution that arose was the translation system—the recipe for making custom-made proteins.

In the peptide/RNA world, peptides played significant roles in accelerating the chemical reactions, by lowering activation energy. Some long peptides are good catalysts and show some enzymic activity. Most likely, ten proteinogenic amino acids were abiotically synthesized [67]. These ancestral amino acids gave rise to a limited variety of random peptides and polypeptides; most were useless, without much specificity, but a few were specifically selected for their catalytic activity. The need for both specific and a wide range of protein enzymes became essential in the peptide/RNA world for biogenesis. Peptides are distinguished from proteins on the basis of size and origin. The peptide is short (only few amino acids long), the protein is long (more than ~40 amino acids), folded, and it forms the catalytic center with a fixed start and end. As a result, the protein enzyme is much more versatile for catalytic reaction than the primitive peptide. Peptides and polypeptides could abiotically form in the prebiotic environment, but the proteins could not arise by chance but are coded, because it is achieved after a long evolutionary process. The evolution of peptides to proteins occurred from a small motif of short peptides to longer folded peptides, and finally to proteins that form complex catalytic centers with almost unlimited possible functions [24]. Darwinian selection provided the driving force for the evolution of specific protein over peptide, so that protein synthesis became essential to the protocell function. These coded proteins were custom-made by translation machines consisting of a repertoire of RNA and protein molecules, and they were highly specified for protocellular functions. The evolution of protein was a long evolutionary process that was driven by incremental advances of the translation machinery, which facilitated the transition from random, simple, peptide produced through an abiotic process, to the eventual production of specific, complex, proteins by RNA-directed protein synthesis.

One of the early manifestations of the transition from peptide to protein is the emergence of the ‘bridge peptide’ that facilitated the aminoacylation of RNA to specific amino acid. The bridge peptide is a short peptide and stereochemical interactions mediate its property to bind a specific RNA with a specific amino acid. Hybridization-induced proximity of short aminoacylated RNAs led to the emergence of bridge peptides, which were capable of stimulating the interaction between specific RNAs and specific amino acids [24]. Eventually, bridge peptides would give rise to protozymes, urzymes, pre-aaRS, and aaRS. The proposed transition mechanism from peptide to proteins, aided by the translation machines, provided a continuity of functions so that each subsequent step was an improvement.

The evolution of complex information system must consist of plausible, elementary steps, with each conferring a distinct advantage on the evolving ensemble of genetic elements. Here, we map the emergence of potential informational and catalytic oligomers, derived from the assembly of building blocks, and reconstruct the probable steps that lead to the translation machinery and the genetic code. It is well-known that modern protein synthesis proceeds with the participation of 20 amino acids, ribozymes, tRNA, various enzymes, including aminoacyl tRNA synthetase (aaRS), mRNA, ribosomal RNA, ribosomal proteins, ribosome, a considerable number of proteinous factors, ATP, GTP, etc. More than 120 species of RNAs and proteins are involved in the process of protein synthesis [65]. The most important steps include: base pair complementarity, the origin of ribozyme, the origin of tRNA, the origin of aminoacyl-tRNA synthetase, the origin of mRNA, the origin of ribosome, the synthesis of protein, and the origin of the genetic code and translation.

The translation system is ancient and highly conserved, and it must have started with protobiopolymers [41,64,65,66,67]. It is most likely that the translation system employed by the cell today has undergone the most extensive and involved evolution; but we do not know this process because the transitional stages have been lost in time. Modern translation requires at least five kinds of macromolecules and amino acids: the set of tRNAs, the set of activating enzymes, the set of amino acids, mRNAs, and ribosomes. Most likely, the evolutionary beginnings of translation could not have involved the interaction of all these components. Thus, the first assumption that we need to make is that the beginning of translation involved, plausibly, a small number of ancestral macromolecules with similar functional capabilities [66]. In our view, the ancestral forms of tRNAs, mRNAs, and aaRS, along with amino acids, were used in the initial stage of translation. Most likely, the ribosome was the last molecular component to appear in the translation machine assembly.

Perhaps, aminoacylated ribozymes, as discussed later (Section 5.3), was the first crude translation machine. Eventually, the ribozyme would give rise to pre-tRNA, and the bridge peptide to pre-aaRS [24]. Later, variants would appear and evolution would produce different by specific aaRS-tRNA-amino acid combinations. Here we start the translation system with two distinct evolutionary precursor macromolecules: pre-tRNA and pre-aaRS that would design and tailor small pre-mRNA molecules for storage information and initiate translation. Eventually, these macromolecules would evolve into tRNA, mRNA, and aaRS. Finally, as the fidelity of translation was refined, ribosomes appeared on the scene, making protein synthesis more efficient.

We identify nine major stages for the origin and evolution of the translation machinery complex and the genetic code leading to the protein synthesis. These possible biochemical pathways are: (1) the selection of amino acids; (2) the origin of RNA; (3) the origin of ribozyme; (4) the origin of transfer RNA; (5) the origin of metabolism; (6) the origin of aminoacyl-tRNA synthetase; (7) the origin of messenger RNA and translation; (8) the origin of ribosome; and finally, (9) protein synthesis. During the emergence of these biochemical pathways, the genetic code and the translation system coevolved with the translation machine.

### 5.1. Selection of Amino Acids

Carbonaceous chondrites carry a large number of amino acids and they were probably the major source of naturally occurring amino acids in the prebiotic cradle. A large pool of amino acids was available in the prebiotic environment. 70 amino acids have been identified in Murchison meteorite [1,4]. It is likely that similar numbers of amino acids were present in the prebiotic environment. Similarly, Miller’s experiments have produced more than 40 different amino acids [69]. Out of 70 amino acids that were likely present in the prebiotic environment, only four L-amino acids, which were most easily formed in the primordial soup (such as alanine, glycine, aspartic acid, and valine), were selected and recruited through molecular recognition, by pre-tRNA and its corresponding pre-aminoacyl-tRNA synthetase. Later, six more amino acids were recruited from the prebiotic environment (such as glutamic acid, leucine, proline, histidine, arginine, and glutamic acid) for tRNA-mRNA-aaRS interactions. These ten amino acids were precursors for the formation of other ten amino acids along prebiotic pathways [69]. The choice of ten primordial amino acids from prebiotic soup for the synthesis of peptides may have been the first product of molecular selection in the *information* age.

### 5.2. The Origin of RNA

The basic constituents of RNA molecules, such as D-ribose, phosphate, and the four bases—adenine (A), guanine (G), cytosine (C), and uracil (U), along with unused nucleotides, were delivered to the hydrothermal crater lake by meteorites [2,3,4]. The polymerization of RNA molecules occurred by mineral catalysis in the prebiotic environment. Nucleotide monomers were linked on the montmorillonite clay substrates of the crater floor in an ATP-rich environment [1,9,22]. The accumulation of phosphates in the vent environment was an important requirement in making the sugar-phosphate backbone of RNA. Nucleotides underwent spontaneous polymerization on the mineral substrate with the loss of water. The resulting product was a mixture of polynucleotides that were random in length and sequence.

**Figure 2:**
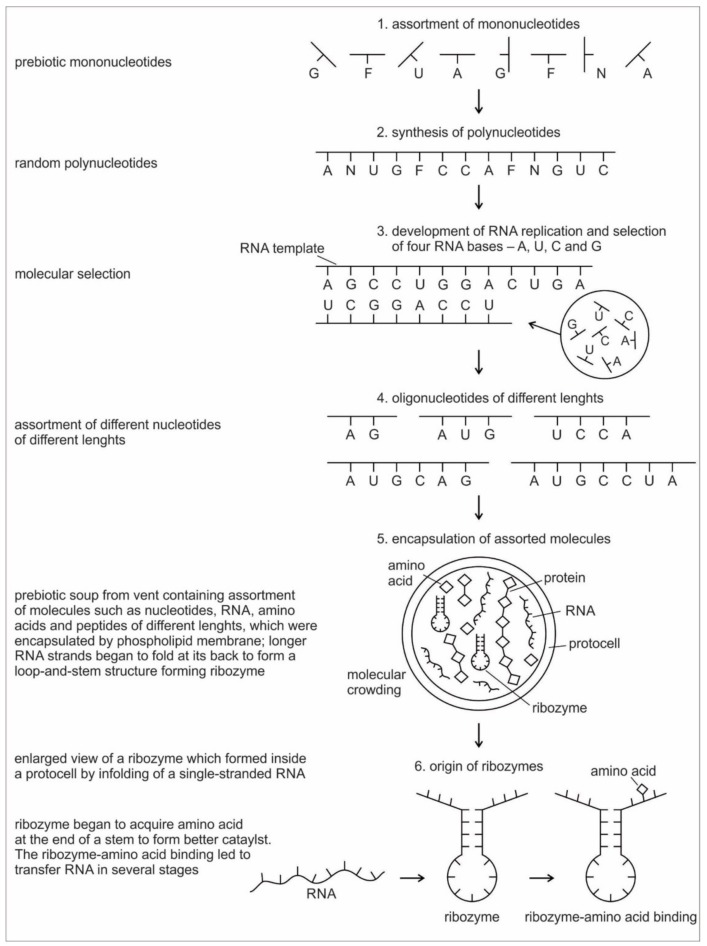
Six main steps represent the early evolution of non-coding RNA in the hydrothermal crater vent environment. In the first stage, there is an assortment of different nucleotides (including some not found in RNA). In the second stage, these nucleotides randomly assemble into polynucleotides by polymerization with the removal of water molecules. In the third stage, four nucleotides, A, U, G, and C were selected out during replication by the Watson–Crick base pairing. In the fourth stage, the nucleotides undergo polymerization to create a mixture of polynucleotides that are random in length and sequence. In the fifth stage, a variety of biomolecules from the vent environment, such as amino acids, mononucleotides, oligonucleotides, and peptides, are randomly encapsulated, creating molecular crowding. Because of crowding, the single-stranded RNA begins to fold, forming the double-stranded stem and single stranded loop that make the hairpin. In the sixth stage, this secondary structure of RNA is shown separately: it forms a ribozyme and begins to act as an enzyme. Stems are created by hydrogen bonding between complementary base pairs. The ribozyme acquires amino acids, at the CCA sequence of the stem, as “cofactors” increasing its catalytic efficiency. The opposite end of the loop consists of three, unpaired bases facing outward, forming a binding site for the attaching of three corresponding mononucleotides. This is the beginning of the emergence of the proto-tRNA.

Six hypothetical stages for the formation of the RNA molecules in the prebiotic environment is shown in Figure 2. It seems unlikely that the prebiotic soup in the prebiotic environment produced only the four bases that were found in RNA—A, U, G, and C—which formed the polynucleotide chain. Certainly, there were other nucleotides (including hypothetical F and N bases in Figure 2), which were incapable of Watson–Crick base pairing. Initially, all of these mononucleotides were randomly polymerized into short oligonucleotides of different lengths by peptide bonding [40]. The process was mediated by natural selection and RNA replication. Natural selection led to the elimination of useless random oligonucleotide sequences during base pairing. From these chaotic assemblages of oligonucleotides, only four bases, such as A, U, C, and G, were selected by exploiting the properties of the Watson–Crick base pairing, whereas hypothetical F and N bases were eliminated. The four standard bases are better than 2 or 6 based on estimates of arbitrary catalysis and the actual pairing energy of standard bases [45]. The four nucleotides were strung together to produce short pieces of oligonucleotide and RNA molecules, which could replicate with the aid of the peptide enzyme. Replication selected prebiotic RNA molecular bases from overwhelmingly large assortment of mononucleotides. These RNA molecules were random and noncoded, being a jumble assortment of nucleotide bases.

Once some rudimentary template-dependent synthetic mechanism allowing for base-pairing was in place, molecules rich in A, U, C, and G were then progressively selected and amplified. These bases joined to form primordial RNA strands of different lengths, which began to self-replicate through a process of base pairing. Short sequences of nucleotides are normally better replicators than long sequences. Longer sequences suffer from an important evolutionary disadvantage; it takes longer to replicate a long sequence than a short once. If a pool of nucleotide sequences containing a range of length is left to code and replicate, then short sequences will dominate and long ones will become extinct [70]. The base pairing principle would later give rise to codon-anticodon hybridization, the origin of messenger RNA, transcription, and replication.

RNA is generally single-stranded and an informational molecule. The self-replication of RNA molecules occurs through a process of base pairing and dissociation. When one RNA strand is made in the vent environment, a second strand would automatically form through base pairing in such a way that cytosine always pairs with guanine, while adenine always pairs with uracil. Consequently, pairing is always between purine and pyrimidine. Because the hydrothermal vents in the crater basins were hot, double-stranded RNA, which formed by base-pairing, came apart through the dissociation of the two chains. When the strands separate, the cycle repeats with another round of base pairing, leading to two more double-stranded RNA molecules, one of which contains the original strand, containing its exact copy. By exploiting the properties of nucleotide base-pairing, coupled with the high temperatures of hydrothermal vent in the crater basin, short pieces of RNA replicated without the aid of any other molecules. Such complementary templating mechanisms lie at the heart of RNA replication, producing a large, more diverse population of RNA molecules (Figure 2).

Because RNA contains a sequence of bases that is analogous to the letters in a word, it can function as an information containing molecule. Moreover, RNA, being a single chain, is free to take any kind of shape; the structure that it can achieve by morphing its shape is wide-ranging, similar to protein. From this basic architecture of a single-stranded RNA molecule, different species of RNAs, such as ribozymes, tRNA, mRNA, and rRNA evolved inside protocells, with a supply of information, distinct in attribute and configuration in response to amino acids. There was a molecular choreography of different RNAs in the prebiotic world that led to the rudimentary translation. The advent and multifunction of different species of RNA molecules signal the transition from the age of *chemistry* to the age of *information*.

### 5.3. The Origin of Ribozyme

**Figure 3:**
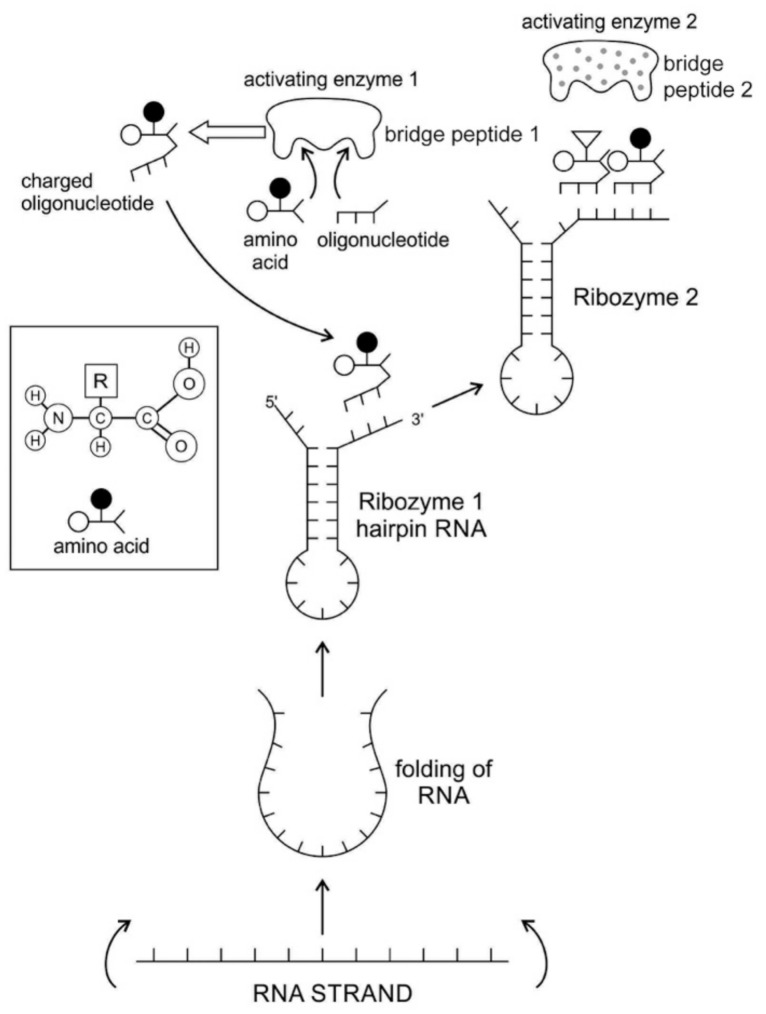
The origin of hairpin ribozyme and its chemical bonding with appropriate amino acid. A single-stranded RNA can develop secondary structure by infolding with double-stranded stem and single-stranded loop forming a hairpin ribozyme. The ribozyme acquired amino acid as cofactor to form a more efficient catalyst [46]. The amino acid is bound to an oligonucleotide (RNA molecule containing only three nucleotides) by an activation enzyme such as ‘bridge peptide’ [24], and the oligonucleotide is bound to the surface of the ribozyme by base pairing (ribozyme 1). The activating enzyme 2 would bind the next batch of amino acid and oligonucleotide is attached to ribozyme 2, forming the peptide bond.

The RNA molecule has a secondary structure. It can form a localized double-stranded RNA stem by base pairing and a terminal loop to form a hairpin structure. In the stem, adenine forms a bond with uracil and cytosine pairs with guanine to form double-stranded RNA. The resulting hairpin structure is a key building block of many RNA secondary structures, such as ribozyme and tRNA (Figure 3). As an important secondary structure of RNA, an RNA hairpin can direct RNA folding, determine interactions in a ribozyme, protect structural stability for mRNA, provide recognition sites for RNA binding proteins, and serve as a substrate for enzymatic reaction [71]. Structurally, RNA hairpins can occur in different positions within different types of RNAs; they differ in the length of the stem, the size of the loop, the number and size of the bulges, and in the actual nucleotide sequence.

Ribozymes are RNA molecules that are capable of catalyzing specific biochemical reaction, similar to the action of protein enzymes. There are different classes of ribozymes, but they all appear to be associated with metal ions, such as potassium or magnesium. Different ribozymes catalyze different reactions, but almost all ribozymes are involved in catalyzing the cleavage of RNA chains in the formation of bonds between the RNA strands.

Most likely, the chemical bonding of a particular amino acid to a small RNA hairpin structure led to the origin of ribozyme. We assume that different kinds of RNA, protein enzymes, nucleotides, oligonucleotides, and amino acids were available in the prebiotic soup. The single-stranded nature of RNA molecule can be bent back on itself, in a hairpin loop, where the stems of the loops are maintained by base pairing to form a three-dimensional structure, just like a protein molecule to act as an enzyme. In some stem-loop configurations, two ends of the stem might remain free, containing the 3’ and 5’ ends. This 3’ end might function as an acceptor stem to form a covalent attachment to a specific amino acid (Figure 3). This small hairpin RNA molecule with specific terminal base sequences acquired the corresponding amino acid as a ‘cofactor’ to improve the catalytic range and efficiency to become initial ribozymes [46]. Many enzymes act with the help of one or more cofactors. The binding of amino acids to a ribozyme resulted in an enhancement of catalytic activity.

Any specific binding between two molecules involves information, as if two molecules ‘recognize’ each other. An amino acid can be linked to an oligonucleotide with three bases by an activating enzyme; the charged oligonucleotide is then bound on the surface of a ribozyme by base pairing and delivers the appropriate amino acid (Figure 2). In this way, ribozymes are capable of producing short peptide chain. This de-novo peptide would play a role in stabilization, in order to become coded. Overtime, the original peptide forming ribozymes will specialize as amino acid specific adaptors. In the peptide/RNA world, different kinds of peptides were synthesized. Aminoacylation of ribozymes would be governed by the availability of amino acids. Most primordial amino acids in the prebiotic environment were alanine, glycine, valine, and aspartic acid. Initially, one kind of an amino acid and one kind of hairpin would be catalyzed by an activating enzyme, perhaps a precursor to the aminoacyl transfer tRNA synthetase, such as bridge peptide, which is a very short peptide that facilitated the emergence of self-sustained RNA-peptide complex supporting a primitive translation [24]. The BP was initially synthesized by chance as a result of the physical proximity of hybridized short, random aminoacylated ribozyme. Each BP is somewhat specific for an amino acid and for its corresponding ribozyme, but the specificity is low. Aminoacylated ribozyme would be involved in complex formation, bringing some of the aminoacylated ribozyme 3’-ends in close proximity. This would promote peptide bond formation between two adjacent amino acids (Figure 3). There was a feedback between ribozymes and bridge peptides. Later, a second amino acid, which is attached to a different hairpin by a different ribozyme, would be added, and so on to create a chain of polypeptide, supporting a primitive proto-translation. It is our contention that the interacting union of a hairpin ribozyme with a specific amino acid is cornerstone in the origin of information, transfer RNA, translation, genetic code, and protein synthesis. The ribozyme would give rise to tRNA and bridge peptide to pre-aaRS to aaRS. This is a combination of model between the one Koonin-developed model [72] describing a polymer transition out of the RNA world and the model of Carter [17] for the role of aaRS in the initial stages of code formation and translation.

A ribozyme has a well-defined tertiary structure that enables it to act like a protein enzyme in catalyzing biochemical and metabolic reactions. The relevance of ribozyme for the origin of tRNA is enormous. Ribozymes, being assembled in the prebiotic vent environment, could not only replicate themselves but would catalyze the formation of specific proteins. The adaptor ribozymes are the precursors of tRNA molecules and they play critical roles in the building of ribosomes. Ribosomal RNA functions as a peptidyl transferase in ribosomes to link the amino acids in protein synthesis, but the framework of transferase is provided by the ribosomal proteins.

### 5.4. The Origin of Transfer RNA

Any model for the development of protein synthesis must necessarily start with direct interactions between RNAs and amino acids. Chemical considerations suggested that direct interactions between the amino acids and the codons in mRNA were unlikely. The protein and mRNA languages seem to be unrelated. Amino acids do not read their codons. Some kind of an adaptor molecule must mediate the specification of amino acids by codons in mRNAs during protein synthesis [27]. The adaptor molecules were soon identified by other researchers as transfer RNAs (tRNAs), which serve as a reading device of mRNA through base pairing. The tRNA molecule binds to amino acids, associates with mRNA molecules, and also interacts with ribosomes to decipher and translate the code of mRNA.

It is generally believed that the first RNA gene, the *Ur-Gen*, was a precursor of modern tRNA [73]. tRNA is the ancestor of all RNAs. It is an ancient molecule that has evolved very little over time. The phylogeny of ribosomes suggests that tRNA is an ancient component of ribosomes that arose in the early prebiotic world [20].

A tRNA molecule is short, typically being 76 to 90 nucleotides in length, which serves as the physical link, a cipher, between the messenger RNA (mRNA) and the amino acid sequences of proteins [30]. Although the tRNA molecule is short, both its primary structure and its overall geometry are undoubtedly more complex than those of any other RNA species [74]. The translation of a message carried in mRNA into the amino acid language of proteins requires an interpreter. The amino acids themselves cannot recognize the codons in mRNA. The tRNA matches appropriate amino acids to the appropriate codons. To convert the three-letters words (codons) of nucleic acids to the one-letter, amino acids of proteins, tRNA molecules serves as the interpreters during translation. Each amino acid is joined to the correct tRNA by a special enzyme, aminoacyl-tRNA synthetase (aaRS).

tRNA participates in two clearly distinct steps in the translation process. The first step comprises the reactions that lead to the charging of the tRNA molecule with an amino acid. The second step comprises the complex reactions in which tRNA transfers its amino acids into a growing protein chain, in response to a specific codon. The chemical reaction catalyzed by the tRNA is simple—the joining of amino acids through peptide linkages. It performs the remarkable task of choosing the appropriate amino acid to be added to the growing protein chain by reading successive mRNA codons. The actual step of translation from mRNA into protein language occurs when amino acids and tRNAs are matched and joined. The translators that do this job are the aminoacyl-tRNA synthetases (aaRS). These enzymes are the only bilingual elements in the cell: they can recognize both the amino acid and the corresponding tRNAs. They are the key element of translation, being the links between the worlds of proteins and nucleic acids. The activation of tRNA occurs when a synthetase uses energy from ATP hydrolysis to attach an amino acid to a specific tRNA. There are twenty such synthetases, one for each amino acid. Together, they make up the complete dictionary for protein synthesis in a cryptic form that relies on tRNAs for decoding into the anticodon language. Each type of amino acid can be attached to only one type of tRNA, so each type of organism has many types of tRNA and more than 20 amino acids. There might be a coevolutionary process in which the anticodons and the corresponding amino acids were progressively mediated by natural selection. As ribosomes appear, tRNAs transport amino acids to ribosomes, where the amino acids are assembled into proteins.

**Figure 4:**
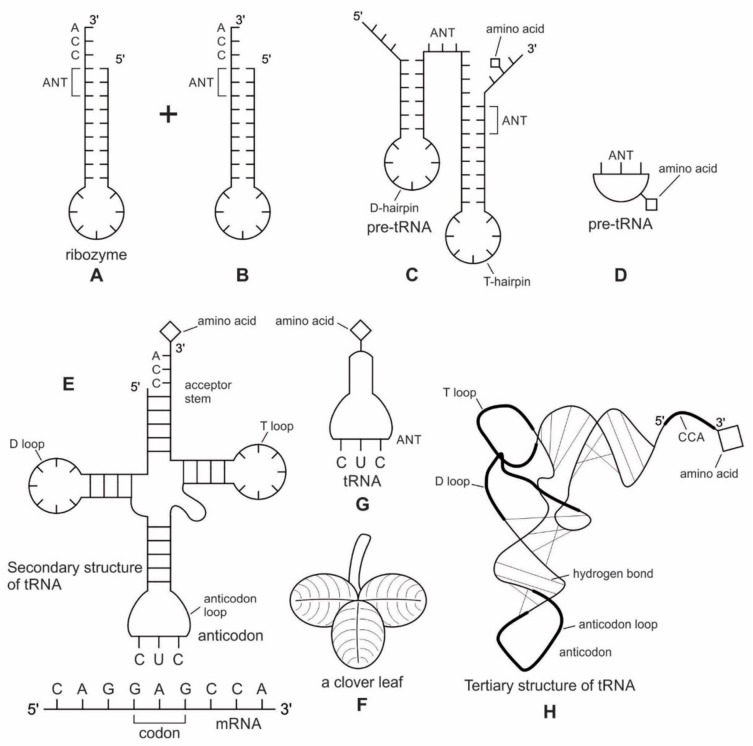
The double-hairpin model of the transfer RNAs (tRNA) formation, showing its evolutionary transitions [76,78,81]. (**A**,**B**), shows a secondary hairpin structure of two RNA molecules (such as ribozymes), each with a stem and a loop: the CCA sequence at the 3’ end of the stem offers a binding site for an amino acid, whereas the 5’ end offers a binding site for phosphorous; (**C**), the direct duplication or ligation of the hairpin structure may have generated a double hairpin structure, creating a D-hairpin and a T-hairpin. An anticodon (ANT) site forms between the two stems. In this newly conFigured pre-tRNA molecule, the acceptor site and anticodon site are now closer together, enabling it to decode a pre-messenger RNA (mRNA) molecule for protein synthesis (see Figure 20); (**D**), a schematic diagram showing the salient features of the pre-tRNA molecule, with the anticodon site; (**E**), the contemporary full-length tRNA molecule could have been formed by the ligation of two half-sized pre-tRNA structures. Its acceptor stem bases and anticodon stem/loop bases, at the tRNA 5’-half and the 3’-half, fit the double–hairpin folding. This suggests that the primordial double–hairpin RNA molecules could have evolved to modern tRNA. This new secondary structure of tRNA resembles a cloverleaf, its anticodon end forms a complementary base pair with the mRNA codon; (**F**), a cloverleaf from nature illustrates the structural similarity with the new tRNA molecule; (**G**), a schematic diagram showing the salient features of the tRNA molecule, emphasizing the anticodon. The tRNA serves a crucial role in matching an amino acid with a specific codon. When tRNA is bound to an amino acid it is called an aminoacyl tRNA. There is now a corresponding tRNA, with an appropriate anticodon, for each amino acid.; (**H**), the cloverleaf secondary structure of tRNA then folds to the L-shaped tertiary structure. At the CCA minihelix end, the aminoacylation site interacts with a large ribosomal unit for a peptide bond formation. The opposite end interacts with the small ribosomal subunit, to decode mRNA triplets through codon-anticodon interactions.

Because of its molecular complexity, the origin of tRNA is controversial. The modern tRNA structure, with its complex configuration and multiple functions, might have originated from a simpler form, such as pre-tRNA molecules to select specific abiotic amino acids in the vent environment (Figure 4A–D). The pre-tRNA molecules with hairpin structures (stem and loop) might have evolved in some evolutionary stages of protein synthesis, originating from a linear chain of RNA [75]. The tRNA has a secondary and tertiary structure. In solution, the secondary structure of tRNA resembles a cloverleaf with three hairpin loops (Figure 4E,F). One of these hairpin loops contains a sequence of three nucleotides, called the anticodon, which forms base pairs with the mRNA codon. The other two loops of the cloverleaf form a D-arm and a T-arm. The unlooped stem contains the free 3´ and 5´ ends of the chain. The CCA sequence at the 3´ end of the acceptor stem forms a covalent attachment to the amino acid that corresponds to the anticodon sequence. The CCA sequence of the acceptor stem offered a binding site for the amino acid. The 5´ terminal contains a phosphate group. Both the anticodon and the acceptor stem sequence correlate with the role of amino acids in folded proteins [76]. The secondary structure tRNA molecule may provide some clue as to its ancestral molecular configuration. The cloverleaf-configuration of tRNA can be derived from a folded ribozyme with a single loop and an attachment site for the amino acid at the end of a stem (Figure 4E).

The most plausible scenario of the origin of the tRNA molecule is based on ribozymes. The chemical bonding of particular amino acids to small RNA molecules with specific base sequences was the crucial step. Perhaps the precursor of tRNA started as a simple ribozyme with a hairpin structure (Figure 4A,B). This ribozyme acquired amino acids at its 3’ end as a ‘cofactor’ (Figure 3): that is, an amino acid was attached to a ribozyme and made it a more efficient catalyst [77]. By using cofactors, the range of specificity of catalytic activity could be increased. One way of attaching an amino acid to a particular point on the surface of the ribozyme is at the end of a single-stranded unlooped stem of the hairpin, which is charged and begins to bind amino acid, which enhances the catalytic function of the ribozyme. With the stabilization of the catalytic reactions, these ribozymes began to participate in the first catalytic cycles. This configuration of a ribozyme linking an amino acid at the end may be the starting point for the origin of tRNA, where the unlooped stems contain the free 3´ and 5´ ends of the chain. This amino acid attachment to ribozymes by a specific assignment enzyme first occurred to make cofactors more efficient catalysts [46].

Aminoacylation of tRNA is an essential event in the translation system. Although, in the modern system, protein enzymes play the sole role in tRNA aminoacylation; in the primitive translation system, ribozymes could have catalyzed aminoacylation to tRNA or ancestral tRNA-like molecules. What was the catalytic function of ribozyme? If it was attaching an amino acid to its own end, it would not be logical that the substrate amino acid is the cofactor at the same time. It has been suggested that this attachment first occurred to make cofactors and it was carried by ribozymes. The RNA world hypothesis implies that the ribozyme functioned as an assignment enzyme to attach a particular amino acid to an ancestral tRNA for aminoacylation before the emergence of aaRS [77]. In the peptide/RNA world, we suggest that the ribozyme was not an aminoacylation catalyst; another molecule, such as bridge peptide, performed this function for the ligation of amino acid with ancestral tRNA [24]. In the early stage of aminoacylation, pre-aaRS, originally a protein enzyme, emerged as an assignment enzyme for charging ancestral tRNA [17,18,19]. In that case, the ribozyme should have another activity that is so advantageous as to help the molecule to survive. In our view, the cofactor function of ribozyme was utilized to form peptide bonds between adjacent amino acids before the emergence of the ribosome. This enzymatic activity may be precursor to that of the Peptidyl Transferase Center of the ribosome that is responsible for peptide bond formation. Another phenomenon in which the intervention of a ribozyme could have been of critical importance is RNA replication [68].

Many studies have suggested that the modern cloverleaf structure of tRNA may have arisen from a single ancestral gene by the duplication of half-sized hairpin-like RNAs by passing through some intermediate structures [76,77,78,79,80,81,82,83]. The linkage of an amino acid with a ribozyme at the end with a hairpin loop might be the starting point for the origin of tRNA, which is a quarter size of the modern tRNA molecule [47]. The relevance of ribozymes in the origin of tRNA is enormous. The equivalent effect of gene duplication might be accomplished by a simple ligation of two identical hairpins of folded ribozymes to create double hairpins, a D-hairpin and a T-hairpin, with an anticodon at the stem bases [82]. RNA ligation is a powerful driving force for the emergence of tRNA, joining two hairpin loops of ribozyme (Figure 4C). During the evolutionary transitions of the pre-tRNA molecule, the double hairpin structure with the D-hairpin and the T-hairpin formed in the ancient prebiotic world, with both the anticodon and the terminal CCA sequence adjacent to the D-hairpin (Figure 4D) [80].

The function of tRNA molecules depends on their precise three-dimensional structure. The cloverleaf tRNA folds into a more compact L-shaped tertiary structure, but each has a distinct anticodon and an attached amino acid (Figure 4H). One arm of the L-shaped tRNA structure has a minihelix with a single-stranded CCA end that is used for attaching a single amino acid; the other arm forms an anticodon loop, with three unpaired bases that may bind with the complementary codon of mRNA. Each tRNA molecule can carry one of the 20 different amino acids at its CCA minihelix end. Each type of amino acid has its own type of tRNA, which binds it and carries it to the growing end of a protein chain during the decoding of mRNA. The CCA end of the minihelix interacts with the large ribosomal subunit to form a peptide bond and the loop end interacts with the small ribosomal subunit for decoding mRNA triplets through codon-anticodon interactions [76].

We suggest that this half-sized hairpin structure of the pre-tRNA molecule acquired some functional capacity for translation before the emergence of tRNA (Figure 4C,D). The pre-tRNA molecule is the evolutionary precursor of the tRNA molecule. Direct duplication or the ligation of half-sized, hairpin-like structures—the pre-tRNA molecule— could have formed the contemporary full-length tRNA molecules, (Figure 4E). The acceptor stem bases and the anticodon stem/loop bases in tRNA in tRNA 5´-half and 3´-half fit together with the double-hairpin folding; this suggests that the primordial double-hairpin RNA molecules could have evolved to the structure of modern tRNA by gene duplication, with subsequent mutations to form the familiar overleaf structure [76,80]. In other words, two pre-tRNA molecules somehow fused together to form a tRNA molecule.

The half-sized pre-tRNA molecule with two loops (D-hairpin and T-hairpin) on one side, and anticodon and acceptor stem region of CCA end on the other side, is structurally and functionally independent and is more ancient than the other-half of the tRNA molecule [81]. This short, self-structured strand of the pre-tRNA molecule possesses a template domain, which is chargeable through interaction with specific amino acids, is probably the predecessor of tRNA (Figure 4C). This pre-tRNA molecule binds, with high specificity, to the amino acid corresponding to its anticodon; this reaction is catalyzed by a specific pre-aminoacyl-tRNA synthetase (pre-aaRS). tRNA evolution is closely linked to aminoacylation. There is a separate tRNA for each amino acid that carries a triplet sequences of nucleotides for anticodon. Later, the anticodon of pre-tRNA will guide the codon formation of the pre-mRNA.

It should be apparent that tRNA molecules must contain a great deal of specificity, despite their small size. Not only do they (1) have the correct anticodon sequences, so as to respond to the right codons, but they must also (2) be recognized by the correct aaRS, to be activated by the correct amino acids, and (3) bind to the appropriate sites on the ribosomes to carry out their adaptor functions. 

An important aspect of the specificity between amino acids and pre-tRNA is that, once this specificity is established, a mechanism for ‘memorizing’ or encoding variations in the sequence of pre-tRNA molecules becomes possible [73]. These pre-selected biomolecules of amino acids emerged from the existing prebiotic soup of the crater vent environments. Among the many essential components of the translation process, assignment enzymes, such as pre-aaRS, evolved to bind a specific amino acid to a pre-tRNA molecule (Figure 4).

### 5.5. The Origin of Metabolism

A prebiotic origin of metabolism is not fully understood. The core structure of the metabolic pathway is very similar across all organisms, which suggests the early origin of protometabolism in the prebiotic world [37]. Catalysts may have played an important role in establishing the early metabolism that ultimately led to the biosynthesis of protein. An intriguing possibility is that modern metabolic pathways emerged through a stepwise process of recruitment of ever more-effective catalysts to catalyze steps in primordial chemical-reaction networks. Metal ions of Fe, Mn, Zn, and Cu were also available in the vent environment, which help to mediate catalysis [10,11,13]. The synthesis of small organic molecules from inorganic precursors, including mineral-mediated synthesis, is probably the stimulus for the origin of metabolism. Large Hadean impacts may have made the atmosphere transiently rich in CO, which may have played a role in the origin of life and in fueling early biological metabolism. CO was an important trace gas on the prebiotic Earth, because it has high free energy and catalyzed the key reactions of prebiotic synthesis and in fueling early biological metabolisms [16].

Crystalline surfaces of common rock-forming minerals, such as pyrite and montmorillonite, are likely to have played several important roles in protometabolism [21]. Mineral surfaces with well-known catalytic properties might have promoted the polymerization of monomers, such as amino acids and nucleic acids. Many of life’s essential macromolecules in the prebiotic world, including enzymes, carbohydrates, and RNA, form from water-soluble monomeric units—amino acids, sugars, and nucleic acid, respectively. Minerals surfaces provide a means to concentrate and assemble these bio-monomers. The polymerization of proteins from amino acids requires the dehydration and condensation mechanism that is precisely found in the fluctuating hydrothermal crater basins. It is well-known that amino acids concentrate and polymerize on clay minerals to form small, protein-like molecules [84]. Such reactions occur when a solution containing amino acids evaporate in the presence of clays. Subsequent studies have shown the adsorption and polymerization of amino acids on varied crystalline surfaces [23].

The problem of the origin of the evolution of metabolism has been recently advanced by the behavior of ZnS, which is capable of harvesting sunlight energy and converting this energy into the formation of chemical bonds of dicarboxylic acid from CO_2_, thus providing the core reactions of universal metabolism before the existence of enzymes [85]. This paper has related how prebiotic metabolites available from simple sunlight promoted reactions can catalyze the synthesis of clay minerals (i.e., a zinc clay called sauconite). The work presents an excellent example of reproductive power of clay minerals and the mechanism by which prebiotic metabolites catalyze their formation. Clay minerals that act as sponges can retain water and polar organic molecules, and they might have played a key role in concentrating and catalyzing the polymerization of key organic molecules such as RNA and protein.

Small molecules—such as amino acids, short peptides, and cofactors—may have catalyzed reactions that are required to produce more complicated organic compounds. Although their catalytic abilities are known to be limited in both acceleration and specificity as compared with later molecular RNA or protein catalysts, some small molecules are remarkably effective catalysts.

The second stage, or metabolism, defined as the first set of reactions that are catalyzed by protein enzymes (and perhaps, ribozymes) prefiguring present-day metabolism, and perhaps already including certain central systems, such as the glycolytic chain and the Krebs cycle [68]. Centrally located within this network are the sugar phosphate reactions of glycolysis and the pentose pathway. This stage of metabolism appeared in the peptide/RNA world and it was modified and refined continuously during the origin of the first cells. As more enzymes were added and started to build their own network, new pathways could have developed.

### 5.6. The Origin of Aminoacyl-tRNA Synthetase

Aminoacyl-tRNA synthetases (aaRSs) are a superfamily of enzymes that are responsible for creating the pool of correctly charged aminoacyl-tRNAs, which are necessary for the translation of the genetic information (mRNA) through the ribosome. aaRSs are very ancient enzymes that are present in all organisms, and are one of the pioneer molecules that are formed by the polymerization of amino acids in incremental steps. Each enzyme catalyzes the activation of a specific amino acid and recognizes a specific tRNA for binding.

The unavailability of activated amino acids was the most critical barrier of protein synthesis. Aminoacylated ribozyme was the pioneer molecule to use the amino acid as cofactor and employed bridge peptide for activation. Later, with the development of tRNA, the activation reaction is catalyzed by specific aaRS, a derived product of bridge peptide. The first step is the formation of an aminoacyl adenylate with an amino acid and an ATP. The next step is the transfer of the aminoacyl group to a particular tRNA molecule to form aminoacyl-tRNA, or a charged tRNA. The mechanism of aaRS formation is well-known [77]. It reveals insight into how and why the tRNA molecule creates its own bilingual enzyme aaRS that can then connect it with the appropriate amino acid. It enhances the selection and sorting of the appropriate amino acids from the prebiotic soup for protein synthesis. Each aaRS is highly specific for a given amino acid. It has a highly discriminating amino acid activation site. Both amino acids and ATP were available in the hydrothermal vent, facilitating a reaction with tRNA to form aminoacyl-tRNA synthetase. Moreover, the proofreading ability by aaRS increases the fidelity of protein synthesis.

How do aaRS choose their tRNA partners? The aaRS recognize, on the one hand, individual amino acids, which they activate via conjunction with ATP; or, aaRS activate amino acids to generate its conjugate with AMP [77]. The synthetase first binds ATP and the corresponding amino acid to form an aminoacyl-adenylate, releasing inorganic pyrophosphate (PP_1_). The next step is the transfer of the aminoacyl group of aminoacyl-AMP to a particular tRNA molecule to form aminoacyl-tRNA. The mechanism can be summarized in the following reaction series:

1. Amino acid + ATP → Aminoacyl-AMP + PP_1_
2. Aminoacyl-AMP + tRNA → Aminoacyl-tRNA + AMP

Thus, the equivalent of two molecules of ATP are consumed in the synthesis of each aminoacyl-tRNA. One of them is consumed in the formation of the ester linkage of aminoacyl-tRNA, whereas the other is consumed in driving the reaction forward. The activation and transfer steps for a particular amino acid are catalyzed by the same aminoacyl-tRNA synthetase. Indeed, the aminoacyl-AMP intermediate does not dissociate from the synthetase. Aminoacyl-AMP is normally a transient intermediate in the synthesis of aminoacyl-tRNA. Synthetases can recognize the anticodon loops and acceptor stems of tRNA molecules. Their precise recognition of tRNAs is as important for high-fidelity protein synthesis, as is the accurate selection of amino acids.

aaRSs come in twenty flavors, with each one being specific to an amino acid and tRNA. These twenty enzymes are widely different, each being optimized to function with its own particular amino acid and the set tRNA molecules that are appropriate to that amino acid. They can be divided into two classes, termed class I and class II. The two aaRS superfamilies evenly divide translation into ten amino acids each. The initial activating enzyme was a bridge peptide that facilitated the aminoacylation of ribozyme (Figure 3). From bridge peptide, protozymes and then urzymes, and finally pre-aaRS and aaRS probably evolved [17,18,19,24]. We speculate that the precursor of aaRS was pre-aaRS, a hypothetical primordial ancestor that gave rise to two classes of aaRS, which are both multidomain proteins. Each aaRS uses different mechanisms of aminoacylation. In our model, the original aminoacylation enzymes were pre-aaRS, a simpler version of aaRS, which must have featured a strong linkage to the anticodon of a pre-tRNA molecule. This linkage must have featured a codon-like, trinucleotide binding site for the adaptor’s anticodon, on the pre-aaRS. We propose that pre-aaRS is an enzyme, including an anticodon, plus a domain that is capable of binding and activating an amino acid and transferring to the pre-tRNA. Pre-aaRS is analogous to ‘protozymes’ and ‘urzymes’ [18,19], but is somewhat more advanced, because it would allow for tRNA/anticodon recognition. Protozymes retain about 40 percent of activity of the full-length of aaRS, even though they contain only about 10 percent as many amino acids. Next came ‘urzymes’, which retain about sixty percent of activity and have the same functional repertoire as the full-length enzymes. We speculate that pre-aaRS would be as long as the urzyme, but it has acquired additional anticodon binding function. The proposed evolutionary path from bridge peptide to protozyme to urzyme to pre-aaRS to aaRS documents increases the complexity of functions and would satisfy the rule of continuity [24].

### 5.7. The Origin of Messenger RNA and Translation

There are two haunting questions regarding the genesis of mRNA: (1) how mRNAs first appeared in the prebiotic environment, before the emergence of DNA and (2) how they evolved in the sequence of nucleotides, with the function of specifying amino acids as the fundamental components for the origin of the genetic code. The primordial mRNA was lost long ago in the *information* stage of biogenesis, leaving no trace of its origin. While existing evidence suggests that the genetic code was influenced by physico-chemical interactions between individual amino acids and strings of nucleic acids [69,86], researchers have yet to piece together the stepwise mechanisms by which it evolved over time.

In the prebiotic world, different species of RNA evolved through cooperation, each with a different function. Although random RNA strands grew during prebiotic synthesis by base pairing, in which some portions of the strand might show codon-like arrangement of nucleobases, they did not contain any genetic information (Figure 2). Moreover, the strings of nucleotide may be haphazardly interrupted by stop and start signals. A fundamental property of protein synthesis is that the amino acids are not added in a haphazard fashion. Their sequence is rigorously imposed by mRNA, which is itself is incrementally formed by tRNA. Each mRNA must be specially made allowing hybridization with tRNA, and specific to each protein.

Here, we propose a new model for the synthesis of custom-made mRNA by tRNA. The evolution of non-random coding mRNA served as the first medium for genetic information that coincided with the development of the genetic code and protein synthesis. As the tRNA molecules began to recognize and react with certain amino acids, they need a separate storage device for safe keeping the information of amino acid assignment. Because the selection of mRNA exclusively depends on codon-anticodon interaction, tRNA begins to make a specific strand of mRNA for the storage of amino acid information (otherwise, it is difficult to see how else mRNA molecules could have become involved with coding the strings of amino acids in a specific manner). We suggest the origin of a new generation of ancestral mRNAs—pre-mRNAs, were created by pre-tRNAs step-by-step. These newly synthesized pre-mRNAs have direct preferences for the amino acids that they tend to encode.

**Figure 5:**
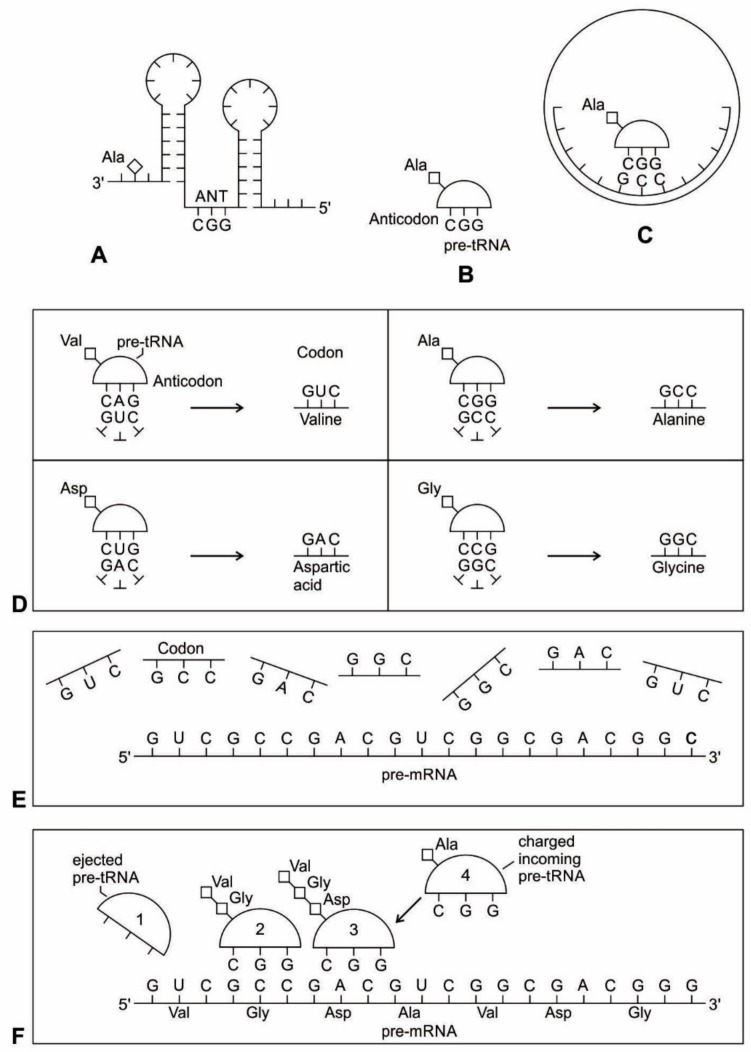
Primitive translation process began with interaction between pre-mRNA and pre-tRNA before the appearance of ribosomes. Pre-tRNA molecule serves as a crucial role in matching a prebiotic amino acid to a specific codon. (**A**), a pre-tRNA molecule with two hairpin loops of 3’ and 5’ terminals and an anticodon (ANT); the acceptor stem at the 3’ end forms a covalent attachment to a specific amino acid that corresponds to the anticodon sequence; (**B**), schematic representation of pre-tRNA emphasizing the 3’ end and corresponding anticodon; (**C**), encapsulated pre-tRNA and pre-mRNA molecule with codon-anticodon interaction; the inner cell membrane acts as a substrate to hold the pre-mRNA molecule in place. (**D**), the anticodon of a pre-tRNA molecule began to hybridize with corresponding nucleotide by base pairing; the triplet nucleotides were kinked to form a codon; In the abiotic stage, the primitive GNC code appeared, which codes four amino acids: valine, alanine, aspartic acid, and glycine [87,88]; (**E**), codons thus produced by pre-tRNAs, began to link in a strand to form a pre-mRNA with coding sequence; (**F**), pre-tRNA and pre-mRNA interactions to form rudimentary translation; the 3’ acceptor end of pre-tRNA gathers appropriate amino acid from the pool and binds it by activation enzyme; an aminoacetyl pre-tRNA with appropriate anticodon hybridizes with codon, ejecting the pre-tRNA; the next aminoacyl pre-tRNA then moves down another codon and repeats the process; amino acid released from the old pre-tRNA begins to join to form a protein chain.

In our model, pre-tRNA molecules begin to select codons via base pairing with their anticodons; these short codon segments are linked to create a longer strand of pre-mRNA step-by-step for storing genetic information. In the pre-tRNA molecule, the site of attachment of the appropriate amino acid is proximate to the anticodon, making the communication between two active sites easier (Figure 5A,B). The physical proximity of the anticodon and the acceptor stem in ancestral pre-tRNA molecules is relevant to a long-sought goal-deriving amino acid/codon pairing rules from an ancestral nucleotide-based receptor-ligand recognition system [66]. A crucial aspect of the origin of pre-mRNA is that codon units are not just randomly added. Instead, the anticodon of pre-tRNA acts as a template to select the matching codon of a pre-mRNA strand. Using the base pairing mechanism, each anticodon of a charged pre-tRNA molecule begins to attract corresponding nucleotides from the prebiotic pool by base pairing (Figure 5D). After hybridization with anticodons, these triplet nucleotides begin to cluster and link together to form small chains of oligonucleotide with codon bases. Several small oligonucleotide chains begin to link to form a longer strand of a pre-mRNA molecule that becomes a database for storing the information of several amino acids (Figure 5E). This coded pre-mRNA became the binding partners for pre-tRNA, enhancing mutual stability and instant cognition. This is a turning point in the origin of translation when a pre-mRNA molecule becomes a digital strip for the storage of genetic information in a separate device in the nucleotide language. Translation is easier to evolve, logically as well as chemically, if there is already a triplet-amino acid assignment that is present. Eventually, several strands of pre-mRNA are joined to form a longer strand of pre-mRNA. These pre-mRNA genes are very short, no longer than 30 to 80 nucleotides. The main feature of pre-mRNA is its heterogeneity for information content. A triplet code sequence with a random codon assignment has very high information content in protein synthesis. With different combinations of codons and varied lengths of pre-mRNA strand, a wide range of amino acid information could be stored for the synthesis of longer protein chain (Figure 5F).

With the emergence of pre-mRNA, the information of anticodon assignment of large pre-tRNA populations can be transferred and stored in a codon message, along the strand of a pre-mRNA molecule. Along the linear strand of a pre-mRNA molecule, digital information for coding amino acids symbiotically emerged with the help of the anticodon of pre-tRNA molecules. Biological information was not only concentrated, but also specified along the strand of a pre-mRNA molecule. Charged pre-tRNA becomes the carrier of a specific amino acid that attached to the matching codon of pre-mRNA.

During the interaction of charged pre-tRNA with pre-mRNA, each aminoacyl pre-tRNA (aa-pre-tRNA) molecule transported and selected specific amino acids for protein synthesis. This is how information enters into the codon of the pre-mRNA molecule in a storage format for a specific amino acid via the anticodon. The information is laid down in the sequences of pre-mRNA, whose quantity is expressed by the lengths of those sequences. These base-pairing attachments between charged pre-tRNA and pre-mRNA provided the structural basis for translation.

The aa-pre-tRNA brings this specific amino acid to this pre-mRNA site during translation, where its anticodon binds to the complementary codon. Initially, four short oligonucleotides, each with a specific codon, were formed and joined in different combinations, specifying four amino acids, such as valine, alanine, aspartic acid, and glycine [88]. This is the first stage of the origin of translation along with the genetic code, involving four amino acids, in which a small number of amino acids were coded by a small number of triplets (Figure 5D). These four amino acids were readily available from the prebiotic vent environment. These oligonucleotides with codons are linked together by random combinations to form a pre-mRNA strand with a coded message (Figure 5E). Once the base sequence of pre-mRNA is stored for a number of amino acids, a rudimentary translation begins to initiate between pre-tRNA and pre-mRNA to synthesize the protein products that provide some modest catalytic, structural, and binding features in the peptide/RNA world. Most likely, the code assignments and the translation mechanism evolved together [75]. Pre-mRNA molecules, which were customized by pre-tRNA, multiplied in the vent environment and linked into longer strands of pre-mRNA to become a genetic reservoir, a digital recipe for proteins synthesis. However, at this stage, pre-mRNA can contain limited genetic information for four amino acids or their multiplied combinations.

During the initial translation process, each pre-tRNA carries its corresponding amino acid on its end (Figure 5F). When a charged pre-tRNA recognizes and binds to its corresponding codon of pre-mRNA, then the growing amino acid chain transfers to the single amino acid of the pre-tRNA. The pre-tRNA molecule begins to translate the codon of the pre-mRNA molecule in the 5´ to 3´ direction. The codon for the first amino acid in the chain (the amino end of the protein) is always at the 5´-end of the pre-mRNA. Likewise, the codon for the last amino acid in the chain is at the 3´-end of the pre-mRNA.

As the translation began along the strand of pre-mRNA, the triplet GUC coded for the amino acid valine. An aminoacyl pre-tRNA entered the site, where it then hybridized the codon. Here, a ribozyme, the precursor to peptidyl transferase of ribosome, performed two critical functions. First, it detached the valine from its pre-tRNA, which was ready to make a growing amino acid chain and released the pre-tRNA. Second, it catalyzed the formation of a peptide bond between that amino acid and the one that was attached to the next codon site. The first pre-tRNA, carrying the amino acid glycine, paired with the codon GCC. With the arrival of the second pre-tRNA, carrying valine, the first pre-tRNA, like a runner in a relay race, passed its glycine to the next, linking with valine and it was ejected. The third pre-tRNA with anticodon CUC hybridized with the next codon, GAC, bearing the aspartic acid, and picked up the link of glycine and valine. The next step repeats when a new aminoacyl pre-tRNA prepares to attach to the next codon site CGG for alanine. Here, it would receive the newly formed polypeptide link of valine-glycine-aspartic acids. To this link, alanine would be added. This is the way that a string of bases of pre-mRNA is translated into a sequence of amino acids. The released amino acids chain of valine, glycine, aspartic acid, and alanine are joined together by a peptide bond to form a newly synthesized protein (Figure 5F). Ribozymes functioned as a catalyst to break the acyl bond holding the growing amino acid chain on the pre-tRNA, and link the new incoming amino acid to the protein chain by a peptide bond. Those ribozymes that were involved in the protein synthesis were the precursors for the peptidyl transferase of the larger unit of the ribosomes.

The association between amino acids and codons—for example, between GUC and valine—is called the code. In this way, the genetic code begins to translate in a rudimentary form, as the short chain of proteins is built according to the instruction from the linear order of codons on the pre-mRNA. The process continues until the pre-tRNA molecule reaches the last codon in the pre-mRNA strand. It stops because there are no more codons to match. The ribozyme is clipped off by the completed protein chain. Once the complete protein is made, the pre-tRNA was discarded, and the pre-mRNA was broken down and its nucleotides recycled. The newly synthesized proteins functioned as enzymes for specific catalysis.

This initial code-programming and storage operation of the pre-mRNA by the pre-tRNA must have occurred within the protective environment of the protocells (Figure 5C). By pairing with the anticodons of the pre-tRNAs, the codons of the pre-mRNA not only selected the appropriate amino acids, but they also help to immobilize the pre-tRNAs. To initiate primitive translation, the pre-mRNA strand needed a substrate where pre-tRNA molecules would sequentially bind one codon after another.

How the primitive translation machinery maintains its proper reading frame is a question of primary importance. In the absence of the ribosomes, the inner surface of the protocell membrane would have served as a substrate for holding the pre-mRNA in position for pairing with the anticodon (Figure 5C). The spherical curved surface of the membrane probably facilitates the movement of pre-tRNA in downstream from 5´ to the 3´ ends of pre-mRNA during translation. This may be the beginning of the origin of reading frame, which is crucial for the reproducibility of translation; the codons of pre-mRNA should be read in a fixed direction with no gap between them.

The availability of several groups of new enzymes enlarged both the structural and the functional capabilities of the pre-mRNA and pre-tRNA molecules, evolving into the more efficient mRNA and tRNA. This evolutionary transformation was characterized by a progressive refinement of the translation system and an increase of the genetic code. As more and more pre-tRNA guided pre-mRNA molecules began to emerge, they were continuously replicated, increasing their population in the prebiotic pool, linking together in various combinations to form longer strands of mRNA molecules. tRNA and mRNA outnumber their precursors pre-tRNA and pre-mRNA through base pairing and replication. These longer mRNA genes arose as replication increased in accuracy. Each mRNA contained about 100 to 200 nucleotides (Figure 5E).

### 5.8. The Origin of Ribosomes

Translation needs one more piece of the molecular machine to continuously make protein in an assembly line—the ribosome. Ribosomes link amino acids together in the order that is specified by mRNA molecules. They provide the environment for controlling the interaction between codons of mRNA and anticodons of aminoacyl-tRNA in the creation of proteins. The translation of encoded information of mRNA and the linking of amino acids that were selected by tRNAs are at the heart of the protein production process. Ribosomes can link amino acids together at a rate of 200/min. Therefore, small proteins can be made fairly quickly. Once a new protein chain is manufactured, the ribosome is released from protein synthesis to enter a pool of free ribosomes that are in equilibrium with separate small and large subunits [77].

**Figure 6:**
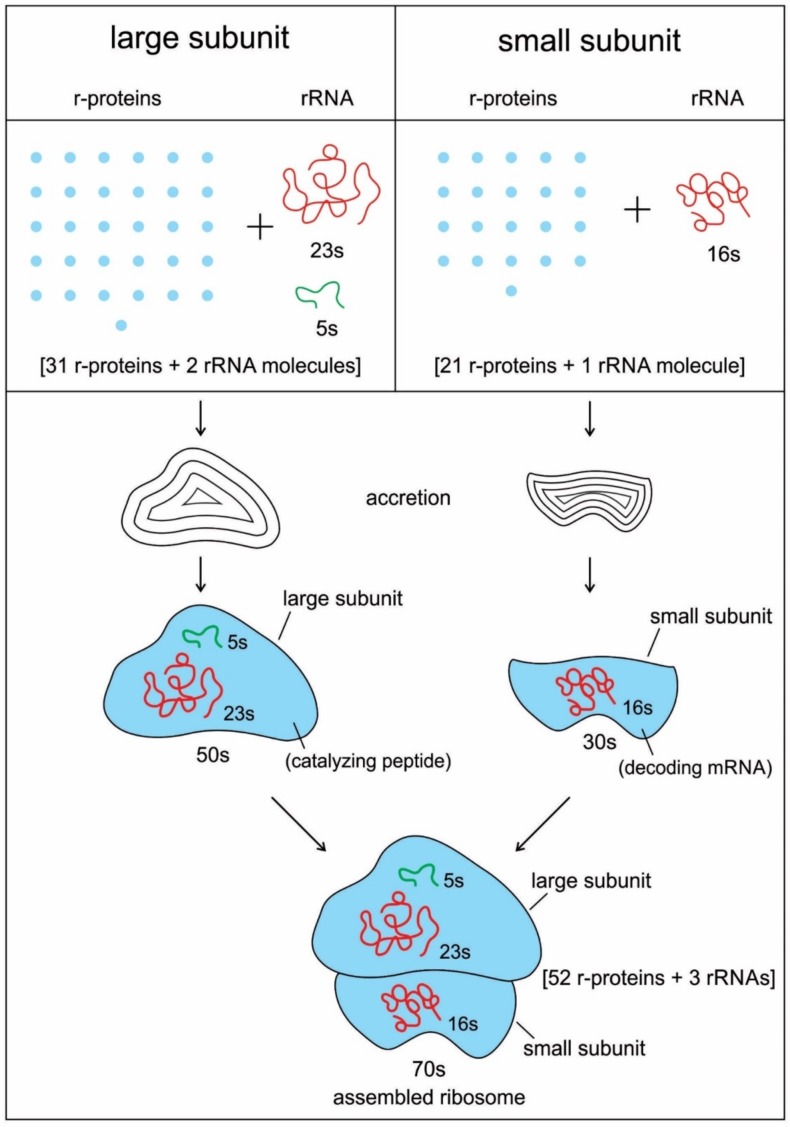
The origin of the ribosome. The ribosome consists of two subunits each with specific roles in protein synthesis. The basic form of the ribosome has been conserved in evolution. Perhaps, the early ribosome was similar to that of modern prokaryotes, which is a large ribonucleoprotein complex of three rRNAs and 52 r-protein molecules. Although ribosomal proteins greatly outnumber ribosomal RNAs, the rRNAs pervade both subunits. There is now evidence that rRNA interacts with mRNA or tRNA at each stage of translation, and that ribosomal proteins are necessary to maintain rRNA in a structure in which it can perform the catalytic functions. Most likely, the symbiotic interactions of ribosomal RNAs and ribosomal proteins gave rise to ribosomes, which grew by accretion. However, there is some controversy whether the small or large subunit appeared first. In our view, both units coevolved by the accretion of ribosomal RNAs and ribosomal proteins.

The ribosome is composed of two-thirds of RNA and one-third protein. It is made of about 50 ribosomal proteins (r-protein) that are wrapped up with four ribosomal RNAs (rRNA) and it is therefore a ribonucleoprotein (Figure 6). Although ribosomal proteins greatly outnumber ribosomal RNA, the rRNAs account for more than half the mass of the ribosome. A bacterial cell may contain as many as 20,000 ribosome complexes, which enable the continuous production of several thousand different proteins, both to replace degraded proteins and to make new ones for daughter cells during cell division. A ribosome physically moves along an mRNA strand, reads the codon sequences of mRNA, and catalyzes the assembly of amino acids into protein chains using the genetic code. It uses tRNAs to mediate the process of translation from the nucleotide language of mRNA into the amino acid language of proteins with the help of various accessory molecules. Each ribosome can bind one mRNA and up to three tRNAs. Central to the development of ribosomes are RNAs that spawn the tRNAs, and a symmetrical region that is deep within the large ribosomal RNA, where the peptidyl transferase reaction occurs [77,89,90].

Recent bacterial ribosomes shed light on the origin, evolution, morphology, and composition of primitive ribosome that emerged in the peptide/RNA world. The bacteria have smaller ribosomes, termed 70S ribosomes, which are composed of two major subunits of unequal size, which are called the large (50S) and the small (30S) subunits; each consists of one or two RNA chains and scores of proteins (Figure 6). The small subunit (SSU) is where mRNA and tRNA molecules interact to read the genetic code, and the large subunit (LSU) is where the growing protein chain is synthesized from the amino acids that are attached to tRNAs. Thus, the small subunit is mainly decoding mRNA, but the large subunit mainly has a catalytic function. In the large subunit, rRNA performs the function of an enzyme and it is termed as a ribozyme. In prokaryotic ribosomes, the small subunit, 30S, is made of one ribosomal RNA and 21 ribosomal proteins, while the large subunit, 50S, is made of two ribosomal RNAs and 31 ribosomal proteins. The two subunits fit snugly in a slot, through which a strand of the mRNA molecule runs between them, after the fashion of a tape through a cassette player. The ribosome glides through the mRNA tape, which then carries out its instructions bit by bit, linking the amino acids together, one by one in a specified sequence, until an entire protein has been synthesized. The ribosomal RNAs are programmed to recognize the codon as it appears on mRNA. When the production of a specific protein is finished, the two subunits of ribosome drift apart [89,90]. Ribosomes only have a temporary existence. The large and small subunits of the ribosome undergo a cycle of association and dissociation during each round of translation. Similarly, once the protein is made, mRNA is broken down and the nucleotides are recycled.

The ribosome evolved prior to the emergence of DNA and the cellular life in the peptide/RNA world. Ribosome evolution is intricately linked to the prior evolution of mRNA, tRNA, and primitive form of the genetic code and translation. The origins and evolution of ribosomes remain printed in the biochemistry of extant life and in the structure of the ribosome. Most theories propose that the ribosome was a functional takeover of a primitive RNA-based translation system in a coordinated series of chemical reactions. RNA is thought to be responsible for the bulk of the ribosome’s work. Recent structures of ribosomes have unambiguously shown that the essential functions of the ribosome, such as decoding, peptidyl transfer, and translocation, all appear to be mediated by RNA [91]. Phylogeny of ribosome suggests that the origin of rRNA is linked to accretionary tRNA building blocks that gave rise to functional rRNA [20]. The decoding center where mRNA is located in the small subunit and it is primarily formed from 16S rRNA. The rRNAs are folded into highly compact and precise three-dimensional structures that form the core of the ribosome. The rRNAs give the ribosome its overall shape. Thus, the widely popular concept of ‘the ribosome is a ribozyme’ was born; the ribozymes must have preceded coded protein synthesis [39].

In recent times, the role of proteins in the origin of ribosomes is gaining currency, implying that the ribosome may have first originated in a peptide/RNA world, where both amino acids and a variety of enzymes were available [9,13,14,15,16,17,18,19,20,21,22,92]. Ribosomal proteins are not passive contributors to ribosome function. They are generally located on the surface, where they fill the gaps and crevices of the folded rRNA. The main role of the ribosomal proteins seems to fold and stabilize the rRNA core, while permitting the changes in rRNA conformation that are necessary for this RNA to catalyze efficient protein synthesis. The ribosomal proteins provide the structural framework for the 23S rRNA, which actually carries out the peptidyl transferase reaction. In the absence of ribosomal proteins, 23S rRNA is unable to serve as a peptidyl transferase activity. The assembly of large and small subunits that are dependent upon ribosomal proteins [17,92]. Several ribosomal proteins assist in the assembly of the large subunit by providing unstructured, highly positively charged protein sequences that bind amino RNA segments together and extend to the center of the subunit [92]. These extensions cooperatively fold with ribosomal proteins to produce the small subunit.

Why would an RNA structure evolve to make proteins if the protein did not already exist that would confer a selective advantage on the ribosomes capable of synthesizing them? The availability of even simple proteins could have significantly enlarged the otherwise limited catalytic function of RNA. Many prebiotic protein enzymes carried out several key functions in the primitive translation system. Moreover, the production of simple proteins had already commenced through the interactions of mRNA/tRNA/aaRS, before the origin of ribosome (Figure 5). Perhaps ribosomal proteins were synthesized during the primitive translation system, which were then recruited to build the ribosome step-by-step. RNAs and proteins developed a symbiotic relationship to create ribosomes in the peptide/RNA world [17,18,19]. These r-proteins took an active part in stabilizing the evolving ribosomes and in interacting with many rRNA sequences. Because the number of proteins greatly exceeded the number of RNA domains, it can hardly come as a surprise that every rRNA domain interacted with multiple proteins in ribosomes [91]. Ribosomes are not entirely ribozymes, but are more accurately ribonucleoprotein (RNP), a complex that can have as many as 62 r-proteins, with only three rRNA molecules (Figure 6). Virtually all r-proteins are in contact with the rRNA. Accordingly, it makes sense that this assemblage is a result of a long and complicated process of gradual coevolution of rRNAs and r-proteins. Both the assembly and synthesis of the ribosomal components must occur in a highly coordinated fashion [20]. Their phylogenetic analysis reveals that the ribosomal protein/rRNA coevolution manifested throughout the prebiotic synthesis process, but the oldest protein (S12, S17, S9, L3) appeared together with the oldest rRNA substructures that were responsible for both the decoding and ribosomal dynamics 3.3-3.4 Ga. Although protein synthesis is largely carried out by different kinds of RNA molecules within the ribosome, such as mRNA, tRNA, rRNA, and peptidyl transferase, aminoacyl synthetase (aaRS) played a crucial role as a protein enzyme that attached the appropriate amino acid onto its tRNA during protein synthesis. The synthetase, in terms of importance, is equal to the tRNAs in the decoding process, because it is the combined action of synthetases and tRNAs that allows each codon in the mRNA molecule to associate with its proper amino acid. Similarly, both rRNA and the 50S subunit proteins are necessary for the peptidyl transferase activity during peptide bond formation, but the actual act of catalysis is a property of the ribosomal RNA of the larger subunit (Figure 6). The cumulative conclusion that seems to be most in accord with biochemical evidence is that the peptide/RNA world preceded ribosome.

The accretion model describes the origin and evolution of ribosomes [20]. Given that the ribosome is quite ancient, it is likely that rRNAs and r-proteins coevolved to build this complex nanomachine. Ribosomes, like the rings of a tree, contain the record of their history, spanning four billion years. Like rings in the trunk of a tree, the ribosome contains components that functioned on in its early history. It accreted to grow bigger and bigger over time. However, the older parts froze after they accreted, like the rings of a tree (Figure 6). Recent phylogenetic work on ribosomal history suggests that both RNAs and proteins contributed to the formation of the ribosome core through accretion, recursively adding expanding segments [20,21]. Ribosomes contains life’s most ancient and abundant polymers, the oldest fragments of RNA and protein molecules. It most likely a molecular relic of the peptide/RNA world [9].

Both ribosomal subunits have separate functions. Peptide bond formation occurs at the peptidyl transferase center (PTC) of the large subunit, whereas the mRNA sequences are decoded on the small subunit. mRNA decoding contributes to the specificity of protein synthesis on the ribosome. In isolation, both of the subunits can perform their respective functions (Figure 6). By itself, the large subunit will catalyze the formation of peptide bonds between aminoacyl-tRNA-like substrates. By itself, the small subunit binds mRNA, and when mRNA is bound, it will bind tRNAs in a codon-specific manner. In an RNA world scenario, the ribosome originated in the peptidyl transferase center of the large ribosomal subunit [93,94]. There are no r-proteins that are close to the reaction site for protein synthesis. This suggests that the protein components of the ribosome do not directly participate in the peptide bond formation catalysis, but rather the proteins act as a scaffold that may enhance the ability of rRNA to synthesize protein. Ribosomes themselves, although being fundamentally ribozymes in nature, still require r-proteins to fold their rRNAs into biologically active conformations and to optimize the speed and accuracy of their functions [85]. The ribosomal surface is an integrated patchwork of rRNAs and r-proteins.

Currently, there is a debate regarding the origin of the ribosomal subunits: which unit came first, the small or the large subunit? It is likely that the PTC of large ribosomal subunit evolved from pre-tRNA molecules by duplication of the minihelix [81]. In this view, the simple function of peptide bond formation at the PTC site came first, and the specifications that were based on the codon sequence came later. In other words, the large subunit of the ribosome came first, followed by the addition of the small unit. However, these proposals do not link the protein synthesis to RNA recognition and do not use a phylogenetic comparative framework to study ribosomal evolution.

Other authors who favor the small unit of ribosome as the first, deduced from the phylogeny of ribosome, offer a contrasting view of the origin of ribosomal subunits [20]. The study suggests that the components of the small ribosomal subunit evolved earlier than the catalytic peptidyl transferase center of the large ribosomal subunit. In this view, the ribosomal RNA and proteins coevolved tightly, starting with the oldest proteins (S12 and S17) and the oldest rRNA helix in the small subunit (the ribosomal ratchet responsible for ribosomal dynamics), ending with the modern multi-subunit ribosome. A major transition in the evolution of ribosomes at around 4 Ga brought independently evolving subunits together by infolding the inter-subunit contacts and interaction with full cloverleaf tRNA structures.

In our view, both the small subunit and the large subunit of the ribosome simultaneously appeared and worked together, because the decoding of mRNA and the peptide bond formation were both essential components during protein synthesis. These two subunits might have coevolved to join during translation and separate after protein synthesis. The rRNAs are folded into highly compact, precise three-dimensional structures to form the core of the ribosome, whereas the r-proteins are generally located on the surface, where they fill the gaps and crevices of the folded RNA and act to fold and stabilize the core [95]. As these two subunits expanded through accretion, eventually arriving at the size of the bacterial ribosome, the accretion stopped, they then bound together during protein synthesis, and finally spilt apart when the ribosome finished reading its mRNA molecule (Figure 6).

If the fundamental functions of the ribosome are based on rRNA, then why are there so many ribosomal proteins, some of which are highly conserved? One explanation is the rRNA does not fold into its functional state in the absence of r-proteins. Another reason for the presence of proteins in ribosomes is that they improve the efficiency and accuracy of the translation [93]. Both rRNAs and r-proteins work cooperatively in ribosomes to perform the multitask procedure of protein synthesis. Harish and Caetano-Anolles suggested that functionally important and conserved regions of the ribosome were recruited and could be relics of an ancient peptide/RNA world [20]. The corollary is that a fully functional biosynthetic mechanism that is responsible for primordial peptides and ancient r-proteins must have existed that in time was superseded by the ribosome.

According to this accretionary model, very early in ribosomal evolution, rRNA helices interacted with r-proteins to progressively form a core that mediated nucleotide interactions, which later served as the center for the coordinated and balanced RNP (ribonucleoprotein) accretion that evolved into our modern ribosomal function [20]. The early existence of smaller functional units of ribosome, which are capable of carrying out different translational steps, such as peptidyl transferase, decoding, and aminoacylation, along with the development of A, P, and E sites for the positioning of tRNA molecules, can be inferred from the phylogeny. These small functional RNA/protein units were incrementally accreted and then refined by the incorporation of additional rRNA and r-protein molecules. Similarly, the first atomic resolution of the larger of the two subunits of the ribosome suggests that the RNA components of the large subunit accomplish the key peptidyl transferase reaction [96]. Thus, rRNA does not exist as the framework to organize catalytic proteins. Instead, the proteins are the structural units and they help to organize the key ribozyme. A ‘pure’ RNA world is incompatible with the existence of the coevolutionary pattern that is proposed for ribosomal molecules.

Perhaps rRNA, such as noncoding ribozymes, acquired amino acids as cofactors, making them more efficient catalysts. By using cofactors, the range and specificity of catalytic activity can be increased. Ribozymes would have been in greater need of cofactors than protein enzymes, because, without them, the range of reactions that they can catalyze is much smaller [46].

In our endosymbiotic model, rRNAs and r-proteins were brought into close proximity within the plasma membrane to form the building block of the primordial ribosome. The origin of the ribosome precursor through fusion and the accretion of the key components of these ribosomal RNA and protein molecules is the likely scenario. The rRNA and r-protein molecules began to fuse because of a chiral preference and then formed the rudimentary ribosomes. Once the core of the ribosome formed, the mRNA and tRNA molecules were recruited to help in translation through a trial and error method. Once a true mRNA and the core small subunit of ribosome were in place, the ribosome would become increasingly complex by adding early conserved rRNA and r-proteins. Ribosomal proteins played an important role in supporting the ribosome structure and in promoting translation. With the onset of operational coding, tRNA began to assemble amino acids into long chains of proteins. Here, we suggest that a ribosome-like entity was one of the key intermediates between prebiotic and cellular evolution, which formed by endosymbiosis and the fusion of rRNA and r-protein molecules. Once ribosomes were installed inside the protocell membranes, the translation system was greatly improved.

In vitro constructions of ribosomes can shed new light on the mechanism of protein synthesis and provide deeper insights into the way that nature has assembled this complex machine. Working with *E. coli* cells, natural ribosomal proteins were combined with synthetically made rRNA, which self-assembled in vitro to create semi-synthetic, functional ribosomes [96,97,98]. Comprising 57 parts—three strands of rRNAs and 54 proteins—an artificial ribosome (termed Ribo-T), in which two subunits are tethered together by a short length of RNA, is able to carry out normal translation and pump out custom-made proteins. The ability to make ribosomes in vitro is a process that mimics nature and opens up new avenues for the study of ribosome synthesis, suggesting the coevolution of ribosomal RNAs and proteins.

### 5.9. Protein Synthesis

We have now reviewed the emergence of all major components of the translation machinery for protein synthesis. Translation of the mRNA template converts nucleotide-based genetic information into the ‘language’ of amino acids to create a protein product. Translation requires the input of an mRNA template, tRNAs, aminoacyl-tRNA synthetases, ribosomes, and various enzymatic factors. The tRNAs function as the adaptor molecules that transport amino acids to ribosomes in response to codons in mRNAs, where peptidyl transferase catalyzes the addition of amino acid residues to the growing protein chain in protein synthesis by means of peptide bonds. The ribosomes serve as the sites for protein synthesis and they link amino acids together in the order specified by mRNA. They always translate the mRNA from the 5’ to the 3’ direction, like a sliding machine.

Proteins have a modular chemical structure that allows for the construction of widely different molecular machines using the same basic set of amino acids, each with a different size and chemical character. Protein synthesis requires the concerted effort of dozens of different enzymes. 20 tRNA molecules, each with their own dedicated synthetase enzyme, are built for 20 amino acids. Modern protein synthesis proceeds with the participation of 20 amino acids, tRNA, mRNA, ribosomes, various enzymes, including aminoacyl-tRNA synthetase, ribozymes, peptidyl transferase, and a considerable number of proteinous factors, ATP, GTP, etc. More than 120 species of RNAs and proteins are involved in the process of protein synthesis [65]. These biomolecules were related, encapsulated, and interacted with each other in complex ways, like an autopoietic machine. Yet, the whole series of molecules in the translation process functioned with astounding precision, in a kind of molecular choreography, which gave birth to the universal genetic code.

**Figure 7:**
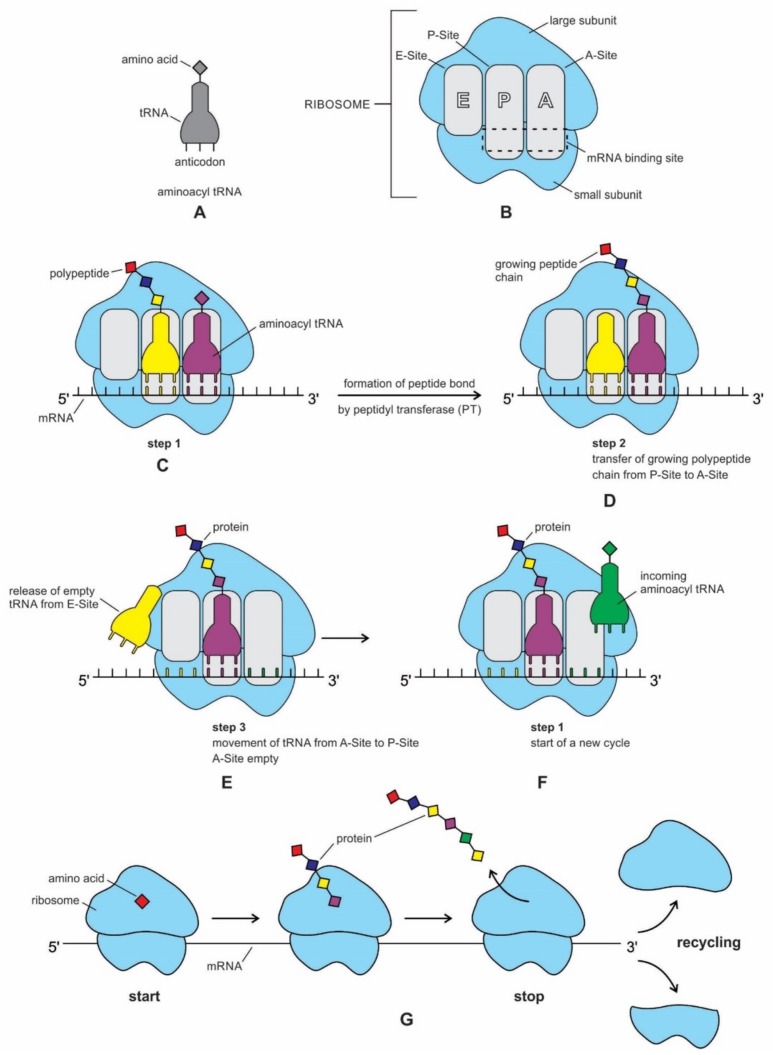
The translation machinery of the ribosome, where the mRNA message is decoded. The ribosome provides the substrate for controlling the interaction between mRNA and aminoacyl-tRNA. (**A**), an aminoacyl tRNA, with appropriate anticodon. (**B**), each ribosome has a binding site for mRNA, and three binding sites for tRNA. The tRNA binding sites are designated E-, P-, and A-sites (for exit, peptidyl-tRNA, aminoacyl-tRNA, respectively). The small subunit contains the binding site for mRNA. Translation takes place in a four-step cycle (**C**–**F**) that is repeated over and over during the synthesis of protein. (**C**), in step 1, an aminoacyl-tRNA, with appropriate anticodon, enters the vacant A-site on the ribosome where it hybridizes with a codon. (**D**), in step 2, the carboxyl end of the protein chain is uncoupled from the tRNA at the P-site, then joined by a peptide bond to the free amino group of the amino acid linked to the tRNA at the A-site. This reaction is catalyzed by an enzymatic site in the large subunit, called peptidyl transferase (PT). (**E**), in step 3, a shift in the large subunit (shown by arrow) relative to the small subunit in the 3’ direction, moves the two tRNAs into the E- and P-sites of the large unit, and then ejects the empty tRNA from E-site. (**F**), in step 4, the small subunit moves exactly three nucleotides along the mRNA molecule, bringing it back to its original position relative to the large subunit. This movement resets the ribosome with an empty A-site so that the next aminoacyl-tRNA molecule can bind. The cycle repeats when the incoming aminoacyl-tRNA binds to the codon of the A-site; (**G**), summarizes the life cycle of the ribosome during its translation [77,99].

The structure and function of the modern ribosome during translation are well-known in the literature and they will not be repeated here [77,99]. In the ribosome, there are three stages and three operational sites that are involved in the protein production line and all work in harmony. During the initiation stage, a small ribosome subunit links onto the ‘start end’ of an mRNA strand. Aminoacyl-tRNA also enters site A of the ribosome. The production of the protein has now been initiated. The second stage, elongation, consists of joining amino acids to the growing protein chain, according to the sequence that was specified by the message. The incorporation of each amino acid occurs by the same mechanism. In the termination stage, the ribosome reaches the end of the mRNA strand, a terminal, or ‘end of the protein code’ message. This registers the end of production for the particular protein that was coded by this strand of mRNA (Figure 7).

Translation is not the end of the protein synthesis process. Once released from the ribosome, the long chain of amino acids will spontaneously fold in intricate contortions into a unique three-dimensional configuration and proper characteristic shape: some parts form sheets, while others stack, curl, and twist into spirals. The sequence of amino acids determines the shape and conformation of a protein and, thereby, all of its physical and chemical properties. A protein molecule spontaneously folds during or after biosynthesis, but the folding process depends on the solvent, the concentration of salts, the temperature, the possible presence of cofactors, and the molecular chaperons [99]. Proteins must fold in specific ways to function properly.

## 6. The Origin and Evolution of the Genetic Code

A code is a set of rules that establish a correspondence between the objects of two independent entities. The genetic code is a correspondence between codons and amino acids. It is the universal language of life. It defines the rules by which information that is stored in mRNA sequences is translated into the corresponding amino acids sequences to proteins. The genetic code is universal; it is the same for all organisms, from simple bacteria to eukaryotes to animals to humans. The genetic code maps the three-letter words, in a four-letter alphabet, of the mRNA language (4^3^ = 64 codons) to the protein language alphabet of 20 amino acids. Before focusing on the origin of the code, let us consider its most important properties. The universal genetic code consists of 64 codons that specify 20 amino acids, and the start and stop sites. The large number of codons is due to redundancy in the code; that is, several codons may specify the same amino acids. All but two of the amino acids (methionine and tryptophan) have more than one codon, many have two, one has three, several have four, and two of them have six codons (Table 1). The amino acids that are more often used in proteins are specified by a greater number of different codons. No codon goes unused. The genetic code has redundancy, but no ambiguity. For example, although codons GAU and GAC both specify aspartic acid (redundancy), neither of them specifies any other amino acid (ambiguity). The genetic code is nonoverlapping, meaning that the ‘words’ follow each other without gaps or overlaps. Each codon in mRNA specifies one amino acid in the protein product. The code is also comma-free. There are no commas or other forms of punctuation within the coding regions of mRNA molecules. During the translation, the codons are consecutively read. The code is ordered. Multiple codons for a given amino acid and codons for amino acids with similar chemical properties are closely related, which usually differ by a single nucleotide [65,66]. The arrangement of the genetic code is distinctly non-random and is such that neighboring codons are assigned to amino acids, with similar physical properties. Hence, the effects of translation error are minimized with respect to reshuffled codes. The digital information in the linear sequence of nucleotides in mRNA is translated into analog sequences of amino acids in proteins according to the genetic code [61]. The vast majority of living organisms follow the same universal genetic code. The most important exceptions to the universality of the code occur in the mitochondria of mammals, yeast, and several other species.

The table of universal (or standard) genetic code, showing the association of each three-letter code to its respective amino acid, is a little dictionary, a Rosetta stone, just as Morse code relates the language of dots and dashes to the twenty-six letters of the alphabet (Table 1). The very existence of two languages (with the code being a translational intermediary) implies a directional course of evolution. The table is not a random accident, but rather it is the result of very specific selection. The mRNA is a linear polymer of four different nucleotides and it is consecutively read in groups of three nucleotides (codons) to form the ‘words’ of the message without any comma. This is known as an ‘open reading frame’. Every sequence in mRNA can be read in its 5’ → 3’ direction in three reading frames. Each three-base codon stands for a single amino acid, so there are 64 possible combinations of three nucleotides. The arrangement of the codons in the universal code is highly nonrandom. The code has been confirmed by several experimental methods [77,99].

The origin of the genetic code remains elusive, even though the full codon catalog was deciphered over 50 years ago [100]. It is still not clear why the genetic code might have originated in the prebiotic world leading to the *information* age. Perhaps some critical biomolecules in the prebiotic environment were cooperative and they began to attract each other. A stereochemical relation between some amino acids and cognate anticodons/codons is likely to have been an important factor in the origin of the genetic code. The biosynthetic relationships between amino acids and RNAs are closely linked to the organization of the genetic code. Different species of RNAs and proteins were manufactured by molecular machines during this stage, and all of the manufacturing processes require not only physical quantities, but also additional entities, like sequences and coding rules [101]. 

The accuracy of the genetic code translated depends on two-steps in protein synthesis: precise decoding of mRNAs and accurate synthesis of aminoacyl-tRNAs. aa-tRNAs are made by aaRSs, which match specific amino acids with the corresponding tRNAs, as defined by the genetic code. Thus, the crucial feature of the genetic code is the attachment of particular amino acids to tRNA molecules, a step that is carried out by assignment enzymes, such as aminoacyl-tRNA synthetase. We are suggesting this attachment first occurred between pre-tRNA and specific amino acid and it was carried out by pre-aaRS enzyme. Since various enzymes were available in the Peptide/RNA world, we propose that the functional enzyme for binding an amino acid to its corresponding tRNA was pre-aaRS from the beginning, not ribozyme, as previously suggested by other workers [46]. 

Although we know which codon encodes which amino acid, we do not know why the specific codon assignments take their actual form. Why are there exactly four nucleobases in mRNA? Why does life use 20 amino acids for making proteins, when 70 amino acids were available in the prebiotic environment from the cosmic source [4]? If the code evolved at a very early stage in the history of biosynthesis, perhaps during its prebiotic phase, the four nucleotides in mRNA and 20 amino acids in proteins may have been the most promising case for optimization by natural selection for chemical reactions that are relevant at that stage. Perhaps it is simply a ‘frozen accident’, a random choice that just locked itself in, and remained, mostly, unchanged once the optimal design was reached [27]. Any change of codon reassignment may be lethal, because it would trigger mutation, which would be dispersed throughout all proteins in the cell. This accounts for the fact that the code is universal in all organisms from bacteria to humans. To account for the uniform code in all organisms, one must assume that all life evolved from the last universal common ancestor (LUCA). Since then, the universal code remains unchanged for the last four billion years. 

### 6.1. The Origin of the Genetic Code

Although multiple hypotheses have been proposed to explain why codons are selectively assigned to specific amino acids, empirical data is extremely rare and difficult to obtain, leaving many theories in the realm of conjecture. Three main concepts on the origin and evolution of the genetic code are:

(1) stereochemical theory, according to which codon assignments are dictated by physico-chemical affinity between amino acids and the cognate anticodons or codons; perhaps, tRNA molecules matched their corresponding amino acids by their stereochemical affinity [72,73,100,102,103,104]. Simply put, the hypothesis proposes that symbols in the genetic code (anticodons or codons) may directly bind to the objects (amino acids) that they stand for.
(2) Coevolutionary theory, which suggests that the code structure coevolved with the amino acid biosynthesis pathways [68,86,105]. This theory suggests that the genetic code is primarily an imprint of the biosynthetic pathways forming amino acids. There are two generations of amino acids: the ten primary or primitive amino acids were formed under prebiotic conditions; they serve as a starting point for the synthesis of the remaining ten amino acids that derived from the first set. What happened afterwards, is that some primitive systems evolved the ability to manufacture the secondary amino acids, and also eventually the primary amino acids.
(3) Adaptive theory, which postulates that the structure of the code was shaped under selective forces that made the code maximally robust, usually some kind of error minimization [76].

Many other models have emerged as addendums to one of the main models or as some form of hybrid. We believe that these three theories are not mutually exclusive and are compatible with the peptide/RNA world, because aaRS play a crucial role in translation. Without aaRS, however, tRNA molecules could not be matched with their corresponding amino acids.

It has long been conjectured that the universal genetic code (Table 1) evolved from a simpler primordial form that encoded fewer amino acids [27]. The earliest peptides started with 10 amino acids, which have been produced in prebiotic chemistry experiments [69,84]. Any model for the evolution of an early code and translation apparatus in the pre-DNA stage will have to provide conditions that allow for tRNA and mRNA of various enzyme factors not only to coexist, but also to coherently grow and to evolve optimal function. We have suggested that the first amino acid was incorporated as a cofactor by aminoacylated ribozyme via bridge peptide, thus initiating proto-translation and the genetic code. The ribozyme would give rise to tRNA and bridge peptide to aaRS [24]. Our proposed biochemical pathways for the origin of the translation favor three distinct phases for the origin of the genetic code. The early stage of coding might have been initiated in a peptide/RNA world by stereochemical interactions between pre-tRNA and amino acids, leading to the birth of pre-mRNA molecules for storing genetic information. The activating enzymes were pre-aaRS, precursors to modern aminoacyl-tRNA synthetases, which bound specific amino acid to corresponding pre-tRNA molecules. Subsequently, the code expanded with the involvement of more amino acid-tRNA ligations by aaRS and the progressive elongation of the mRNA strand for accommodating increasing genetic information. Finally, the code further expanded for redundancy and was optimized through codon reassignment with the emergence of ribosomes, which bound mRNA and tRNA to synthesize proteins (Table 1).

The stereochemical hypothesis postulates that a physico-chemical affinity between amino acids and cognate anticodons or codons is determined by the structure of the code [75,102,103,104,105,106]. The close linkage between the physical properties of amino acids and tRNA molecules was likely an essential step for the origin of code. A stereochemical relation between some amino acids and cognate anticodons/codons is likely to have been a significant influence in the earliest assignments [107]. It is possible that the chiral d-sugars in RNA attracted the chiral L-amino acids as a stereo pair. An exhaustive analysis of the stereochemical concept suggested that the genetic code originated before translation [108]. The stereochemical theory is supported by RNA aptamer experiments, in which RNA molecules evolved to bind specific amino acids [96]. Such experiments have provided critical empirical data, demonstrating the association of codon triplets with amino acids.

It is generally believed that specificities for some amino acids come from stereochemical interactions. However, the stereochemical hypothesis possesses its own set of problems. First of all, it is not preserved as a way of recognition today. If it was a factor for codon/amino acid recognition at the beginning, then it would be extremely important to be replaced later by protein type of recognition. Second, as demonstrated in experiments [104], for aptamer-amino acid interactions, five out of eight amino acids have close to random specificities, with just one exception—arginine—showing a significant specificity to recognize its own codon. However, arginine is not even in the list of initial codon formation (Table 1). As discussed earlier, bridge peptide is capable of stimulating interaction between specific RNAs and specific amino acids [24]. Bridge peptide seems to be an alternative to stereochemical way of recognition in the beginning.

Other experiments suggest that anticodons are selectively enriched near their respective amino acids in the ribosomal structure and such enrichment is correlated with the universal genetic code [109]. Ribosomal anticodon-amino acid enrichment reveals that specific codons were reassigned during code evolution. These authors concluded that anticodon-amino acid interactions shaped the evolution of the genetic code.

tRNA serves as the physical link between the mRNA and the amino acid, because mRNA could not make direct bond with an amino acid. It is a decoding device that reads the triplet genetic code of mRNA and it causes the insertion of codon specific amino acids in a growing protein chain during the process of translation. The specific coding between codon and amino acids takes place in a two-step process via tRNA. For each amino acid, there is a corresponding tRNA molecule for which it has the intrinsic affinity. tRNA molecules function as adaptors by mediating the incorporation of proper amino acids into proteins in response to specific nucleotide sequences in mRNA. The amino acids are attached to the correct tRNA molecules by a set of activating enzymes, aminoacyl-tRNA synthetases [110].

Since all tRNAs have similar structures, the identification must take place on a sequence level in combination with subtle structural variations. In most known cases, the anticodon bases are part of this set of identity elements. In *E. coli*, the tRNA species for 17 out of 20 amino acids are recognized by their anticodons [111]. Its matching codon is also recognized as data, the information of a specific amino acid. tRNA-codon recognition has always been assumed to be result of base-pairing. Anticodon-codon pairing might have initiated the first primitive translation.

It is generally believed that the linking of amino acids to tRNAs played a crucial role in the origin of coding and translation. The original amino acid-binding motifs could have been the actual anticodons of tRNAs. Several authors have proposed that abiotic tRNA molecules could have bound some abiotic amino acids to either improve stability or expand their functional capabilities, or both [89,107,112]. Without this initial amino acid binding site, it is difficult to see how else the tRNA molecules could have become involved with coding specific amino acids. tRNA-amino acid pairing interactions were a prelude to the code. Thus, our first clue to the origin of the code is to decipher how primordial tRNAs and amino acids were related by molecular recognition and chemical principles [113].

**Figure 8:**
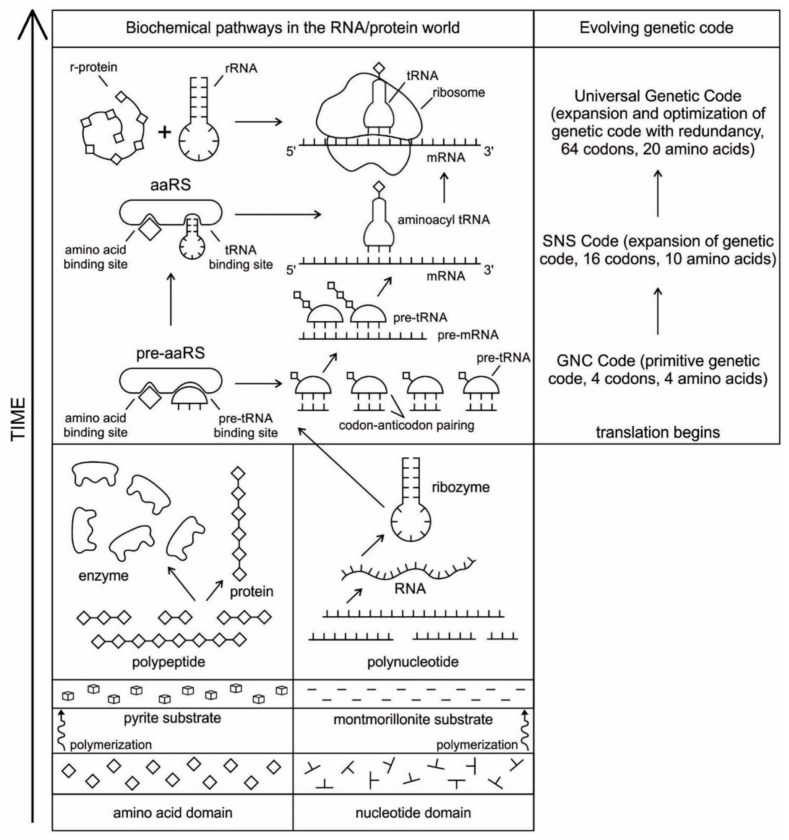
The inferred biochemical pathways for the origin of translation and the genetic code in the RNA/peptide world. The hydrothermal crater vent was crowded with several monomers such as amino acids and nucleotides, which were polymerized on the mineral substrate to form various peptides and RNAs. As ribozymes evolved into pre-tRNAs, each pre-tRNA molecule captures specific amino acid, assisted by pre-aaRS enzyme. Eventually, anticodons of pre-tRNAs created custom-made pre-mRNAs for the storage of genetic information. The interaction between pre-tRNA and pre-mRNA generated small protein chain by rudimentary translation and GNC primitive code with four codon and four amino acids. With the refinement of translation, pre-tRNA evolved in tRNA and pre-mRNA to mRNA with the expansion SNS code with 16 codons, and 10 amino acids. Finally, as ribosome appeared by fusion of ribosomal proteins and RNA, it facilitates high-fidelity translation, leading to universal genetic code with 64 codons and 20 amino acids.

In contrast to stereochemical hypothesis, the coevolution theory suggests that the original genetic code specified a small number of abiotic simple amino acids, and that, as more complex amino acids were synthesized from these precursors, some codons that encoded a precursor were ceded to its more complex products. Wong [69,86,105,114] championed the coevolution theory, and further expanded by Di Giulio [87]. It proposes that primordial proteins consisted only of those amino acids that were readily obtainable from the prebiotic environment, representing about half of the twenty amino acids of today, and the missing amino acids entered the system as the code expanded along the pathways of amino acid biosynthesis. The coevolution theory postulates that prebiotic synthesis could not produce 20 modern amino acids, so a subset of the amino acids had to be produced through biosynthetic pathways before they could be opted for expanded genetic code and translation [75,87]. There are two types of amino acids, depending on whether they were supplied by the prebiotic environment (Phase 1) or were biosynthetically produced (Phase 2) [68,105]. The first phase of amino acids consists of glycine, alanine, serine, aspartic acid, glutamic acid, valine, leucine, isoleucine, proline, and threonine. Phase 2 amino acids include phenylalanine, tyrosine, arginine, histidine, tryptophan, asparagine, glutamine, lysine, cysteine, and methionine. The first phase of amino acids naturally emerged through prebiotic synthesis in the vent environment, before the emergence of ribosomes. They have been identified in meteorites. The ranks of amino acids in this list strongly correlate with the free energy that is available in the vent environment for their syntheses: the most thermodynamically efficient are on the top of the list. These 10 amino acids are considered as old and they were represented in the first stage of protein synthesis [69]. They would play important roles in the primitive GNC-SNS code (Figure 8). Phase 2, the amino acids entered the code by means of biosynthesis from the Phase 1 amino acids with the emergence of tRNA molecules, aminoacyl transferase enzyme, and ribosomes [68,82,105,108].

### 6.2. Early Stage of Code Evolution: GNC Code

The early phase of the evolution of the genetic code is characterized by low fidelities of replication and translation, as well as by an initially low abundance of efficiently replicating units [115]. Hypercyclic organization offers multiple advantages over any other kind of structural organization. This hypercycle model can be built to provide realistic precursors, such as pre-tRNA and pre-mRNA. The interaction between pre-mRNA and pre-tRNA molecules is the beginning of the first stage of the biosynthesis of the templated protein chain, encoded in pre-mRNA. These new generations of amino acids are not only template directed but also sequence-directed [116]. Here, we propose that the interaction between pre-tRNA and amino acids led to the development of the pre-mRNA strand and the primitive GNC genetic code [88,116].

A primordial code must have a certain frame structure, a grammar of rules, otherwise message cannot be consistently read. The GNC hypothesis refers to the origin of genes. It suggests that the universal genetic code originated from a primitive four-amino acid system encoding primitive amino acids (glycine, alanine, valine, and aspartic acid) to create sequence-specific peptide chain [85,88]. The GNC codons include four codons (GGC, GCC, GAC, GUC), which code four primitive amino acids Each letter of GNC represents the following nucleotides: G = G; N = A, U, C, G; and, C = C. The GNC code defines the very earliest phases of the genetic code origin, reflecting the biosynthetic relationships between four amino acids and four codons (Table 1). Perhaps GNC code was promoted by the pre-tRNA/pre-mRNA interaction and coevolution [116,117] (Figure 5A). As amino acids overtook more and more catalytic duties, the genetic information that has been established so far had to be rewritten, a translation into the language of amino acids by specific interaction was inevitable. The translation required a mini dictionary of nucleotide-to-amino acid equivalence; hence, this was the inevitable moment for the genetic code to emerge. To perform protein translation an elaborate machinery of specialized enzymes is necessary. This machinery must be produced step-by-step before translation can take place at all (Figure 5D). It seems reasonable to start this process in simplified form while only using a restricted set of amino acids, such as glycine, alanine, aspartic acid, and valine, which were of prebiotic origin [71].

### 6.3. Transitional Stage of Code Evolution: SNS Code

GNC code evolved into the second generation of the genetic code, called an SNS type, where N arbitrarily denotes any four RNA bases and S denotes guanine (G) and cytosine (S) [80,108]. SNS is composed of 10 amino acids (glycine, alanine, aspartic acid, valine, glutamic acid, leucine, proline, histidine, glutamine, and arginine) and 16 codons (GGC, GGG, GCC, GCG, GAC, GAG, GUC, GUG, CUC, GUG, CCC, CGC, CAC, CAG, CGC, and CGG) [87,88]. The SNS type code shares similarity with the Phase 1 amino acids that were generated in prebiotic synthesis [64]. The remaining ten amino acids are derivatives of the first ten primitive amino acids. Support for the GNC-SNS primitive genetic code hypothesis comes from the following six indices: hydropathy, -helix, ß-sheet and ß-turn formabilities, acidic amino acid content, and basic amino acid content (Table 1). This early genetic code continued to evolve, maximizing its efficiency, until it arrived at its current state, the universal code. This universal code had the edge over the GNC-SNS primitive code, reliability wise, so natural selection would favor it and, through the process of successive refinement, an optimal code would be reached. The universal code is the optimization of functional efficiency to minimize error during translation. 

### 6.4. Final Stage of Code Evolution: Universal Genetic Code

The present genetic code is most probably the outcome of a long selective process, in which many different codes were tested against each other. As more and more biotic amino acids were synthesized and are available in the vent environments, more complex molecules, such as tRNA, mRNA, and ribosomes emerged and produced Phase 2 amino acids. At this stage, the universal code began to appear (Table 1). A direct correlation has been found between the hydrophobicity ranking of most amino acids and their anticodons. In this stage, ribosomes emerged to facilitate high-fidelity translation. tRNA assigned more codons to mRNA and this led to the emergence of the universal genetic code with 64 codons specifying 20 amino acids [100]. The driving force during this process is not only to minimize translation error, but also positive selection for the increased diversity and functionality of proteins, which are made with a larger amino acid alphabet. With 64 codons, the strand of mRNA became longer, forming a continuous sequence with the start and stop sites for protein synthesis. In the ‘codon capture theory’, the number of encoded amino acids is kept constant and is equal to 20, and the coding codons change in the evolution, a key role that is played by the anticodon [104,113].

It has been suggested that the universal genetic code with 64 codons originated from the SNS code, which allowed redundancy [85]. Four codon assignments, corresponding to tyrosine, tryptophan, serine, and isoleucine, were newcomers from the SNS code, suggesting that these amino acids are later additions to the code [84]. This idea is consistent with the view that these four amino acids are later additions of code. Undoubtedly, there were many experiments with a variety of coding methods before adopting the current system, in which 61 codons specify 20 amino acids and three additional codons for the start and stop sites.

The code is obviously not the result of a random assignment of codons to amino acids. It has a structure. Synonyms are grouped. The large number of codons is due to redundancy in the code; that is, several codons may specify the same amino acids (Table 1). Some generalizations can be made regarding the redundancy of the code. For example, similar codons specify the same amino acids to reduce the harmful effects of mutation. For example, GUU, GUC, GUA, and GUG all specify valine. Similarly, amino acids that are used more often in proteins are specified by a greater number of different codons. For example, the most common amino acid, leucine, is coded by six codons (UUA, UUG, CUU, CUC, CUA, and CUG), and the relatively rare tryptophan by one codon (UGG) [46] (Table 1). The expanded genetic code is so universal that there is strong evidence that all life on Earth had a single origin in the universal code before the last universal common ancestor (LUCA) evolved. 

Between the codon and anticodon, there is a paradox in the expanded genetic code. The 20 amino acids that were found in proteins are specified by 61 different mRNA codons. Instead of containing 61 different tRNAs with 61 different codons, though, most bacterial cells contain some 30-40 different tRNAs. Consequently, many amino acids have more than one tRNA to which they can attach; in addition, many tRNAs can pair with more than one codon [75]. 

Although some features of the expanded code may reflect the early version, there are others that appear to be adaptive. The genetic code has certain regularities and structures [115]. There is a strong correlation between the first bases of codons and the biosynthetic pathways of the amino acid that they encode. The first letter of the codon is allied to the precursor of the amino acid. The second letter signifies whether an amino acid is soluble or insoluble in water, its hydrophobicity. Amino acids that have U at the second position of the codon are hydrophobic, whereas those that have A at the second position are hydrophilic. Codons for the same amino acid typically only vary at the third position. The third letter is where redundancy lies with eight amino acids with a fourfold degeneracy, where all four bases are interchangeable. In all cases, U and C are interchangeable in the third position. In other words, the third position of the codon is information-free with much flexibility. Many amino acids are specified by more than one codon. Codons for the same amino acid tend to have same nucleotides at the first and second positions, but a different nucleotide in the third position. The relative lack of criticality is related to the fact that the pairing between the anticodons and codons often enjoy a certain flexibility, so those same anticodons can pair with more than one codon, a phenomenon that is known as wobble [118]. This is why the number of tRNAs and, therefore, of anticodons is smaller than the number of 64 codons, usually ranging between 35 to 45 [76]. Once the code was born, the need to minimize errors might have refined it. The code has been optimized over the eons and is not simply the product of chance, but of natural selection.

A key role of the universal genetic code is to maintain integrity and verify the specificity of each mRNA codon to a particular amino acid. There must be an accuracy strategy of cross checking that could reveal that the mRNA codons and amino acids will directly interact. Various authors have suggested that the original amino acid-binding motifs could have been the actual codons rather than anticodons [117,119]. However, contrawise, we believe the codon-amino acid pairing system might have evolved for code verification at a later stage of code evolution. Initially, anticodons developed between the interactions of pre-tRNA and pre-aaRS [111]. The anticodons selected the codons of pre-mRNA by base-pairing (Figure 5). As the genetic code was refined and optimized, verification on the strings of codon on the mRNA strand began, through quality control, to ensure that each tRNA successfully interprets the amino acid information for protein synthesis with a low error rate. Most likely, the amino acid-codon interaction, which is mediated by aptamers, evolved later for keeping the code error free.

## 7. Coevolution of Translation Machines and the Genetic Code

The contemporary genetic code of protein biosynthesis most likely evolved from a simpler code and process. It has been suggested that the present code is a random accident that is forever frozen in time [27,28], while others have argued that the code, like all other features of organisms, was shaped by natural selection. Both the stereochemical and the coevolutionary hypotheses provide possible mechanisms for the selection of amino acids by RNAs from a large pool of prebiotic soup, which are recruited for protein synthesis. During these processes of selection and recruitment of amino acids, the translation machine and the genetic code evolved. Natural selection has led to codon assignments of the genetic code that minimize the effects of translation errors and mutations during the evolution of the code. The adaptive hypothesis posits that the genetic code continued to evolve after its initial creation, so that the current code maximizes some of the functions.

We accept all three well-known hypotheses—the stereochemical, the coevolutionary, and the adaptive—for the origin and evolution of the genetic code at different stages. We concur with previous researchers that multi-generations of amino acids were sequentially produced [75,88,104,113] as the code expanded, along with the pathways of amino acid biosynthesis. The biosynthetic relationships between different generations of amino acids are closely linked to the evolution of the genetic code. We contend that information evolved along with the translation machines, and it played a vital role in the perfection of the translation process and genetic code. We concur with the view that the genetic code and the translation mechanism evolved together in the prebiotic world [64]. Here, we elaborate this concept of information-based coevolution of the translation machine and the genetic code that may provide a new window into the origins of translation and the genetic code.

The translation machines are an extremely complicated hierarchy of complex macromolecules that are symbiotically related to one another. Yet, the whole functions with remarkable precision. Once the translation machinery complex for protein synthesis is installed step-by-step, information enters into the system via symbiotic interactions of mRNA, tRNA, aaRS, and ribosome. This machinery implements the genetic code. Here, we summarize how such a complex translation machinery would evolve step-by-step into today’s protein-synthesizing machinery, starting from the cosmic building blocks in hydrothermal crater-lake environment (Figure 8). 

The origin of biomolecular machinery likely centered around the tRNA-amino acid alliance, both being ancient molecules in the prebiotic environment [74,117]. Various ‘spare parts’ of biomolecules for building translation machinery were available in the prebiotic soup during the *chemical* stage, from which some few were selected, based upon the chemical affinity between macromolecules. tRNA is the oldest and most central nucleic acid molecule. Its coevolutionary interactions with aaRSs define the specificities of the genetic code. The biochemical pathway that is outlined here for the emergence of the genetic code is the simplest and the most straightforward account of the development of RNA-dependent protein synthesis.

The *information* age emerged from a reciprocal partnership between small ancestral oligopeptides and oligonucleotides. They initially contributed to rudimentary information coding as well as catalytic rate accelerations. It begins with the molecular recognition, attraction, and communication between pre-tRNAs and amino acids, mediated by pre-aaRS. The role of tRNA synthetases in the origin of the genetic code is pivotal. It helps the anticodon of tRNA to pair with the right amino acid. It is the matchmaker between tRNA and its corresponding amino acid. Coevolution, the coordinated succession of structural changes that are mutually induced by the increasingly interacting and growing protein and nucleic acid molecules, played an important role during the origin of translation machinery and genetic code. aaRS coevolved with tRNA and tRNA coevolved with mRNA during the rise of the genetic code specificities. A novel mechanism of how tRNAs are recognized by certain aaRS has been suggested [19]. In this view, tRNAs carry two codes: the well-known anticodon and a second one in the acceptor stem (Figure 4E). These two codes are not arbitrary: the nucleic acid sequence of the acceptor stem and the anticodon code for distinct physical properties of amino acids. In other words, the codon/amino acid pairing reflects the different physical roles the different amino acids play in the structure of full, folded proteins. The genetic coding of three-dimensional (3D) protein structures evolved in distinct stages, initially based on the size of the amino acid and later on its compatibility with globular folding in water.

The genetic code and the translation mechanism evolved together in the prebiotic world [64]. Here, we discuss a simple but effective biological information system that works as a translation system. In Figure 8, we show the proposed biochemical pathways and coevolution of translation machinery and the genetic code in three stages. We outline how early RNAs and protein catalysts developed into the universal coding system that we have today. Our outline is necessarily speculative, but it suggests a series of transitional stages of symbiotic relationships between tRNAs and proteins that may have led to the origin and evolution of the genetic code. Since molecular evolution did not leave any fossil record, some of the transitional stages of the translation machinery are now erased by evolution as the final stage appeared. Thus, no record of code evolution has been detected so far.

Among different species of RNAs, tRNA has a very ancient history and it is more closely associated with protein during synthesis. Pre-tRNA, the tRNA’s ancestor, likely played a central role of primitive translation early on. A stereochemical relation between some amino acids and cognate anticodons of pre-tRNA must have played an important informational role in the earliest assignments [67,104]. Bridge peptides also help in the aminoacylation of ribozymes with specific amino acids [24].

**Figure 9:**
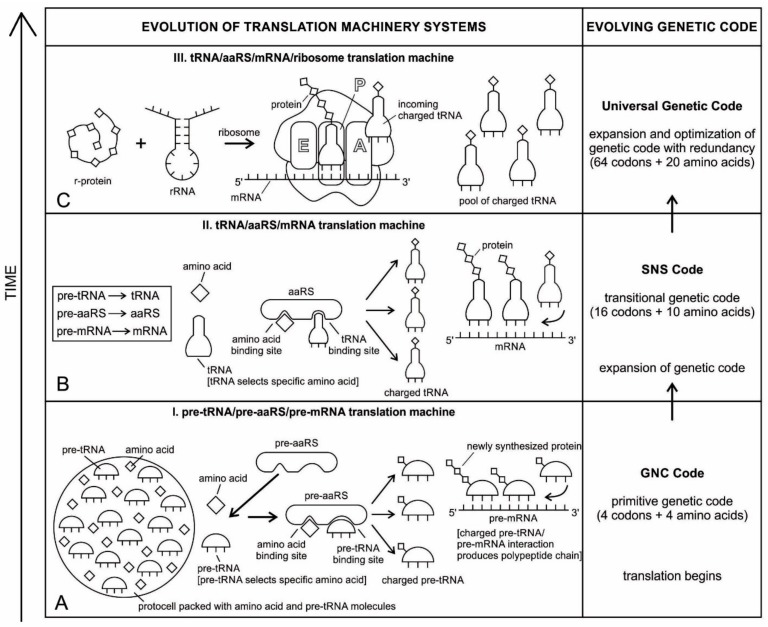
The inferred temporal order of evolution of translation machinery systems showing coevolution of translation machines and the genetic code. In our model, there are three stages of translation machinery systems: (**A**) pre-tRNA/pre-aaRS/pre-mRNA stage when GNC code evolved with the beginning of translation system; (**B**) tRNA/aaRS/mRNA stage when SNS code appeared; and finally, (**C**) tRNA/aaRS/mRNA/ ribosome stage when universal code evolved.

The origin of the code follows closely the biosynthetic pathways of refining the translation machinery complex in three successive stages in the peptide/RNA world (Figure 9):

- pre-tRNA/ pre-aaRS/pre-mRNA machine;
- tRNA/ aaRS/mRNA machine; and finally,
- tRNA/aaRS/mRNA/ribosome machine.

**Figure 10:**
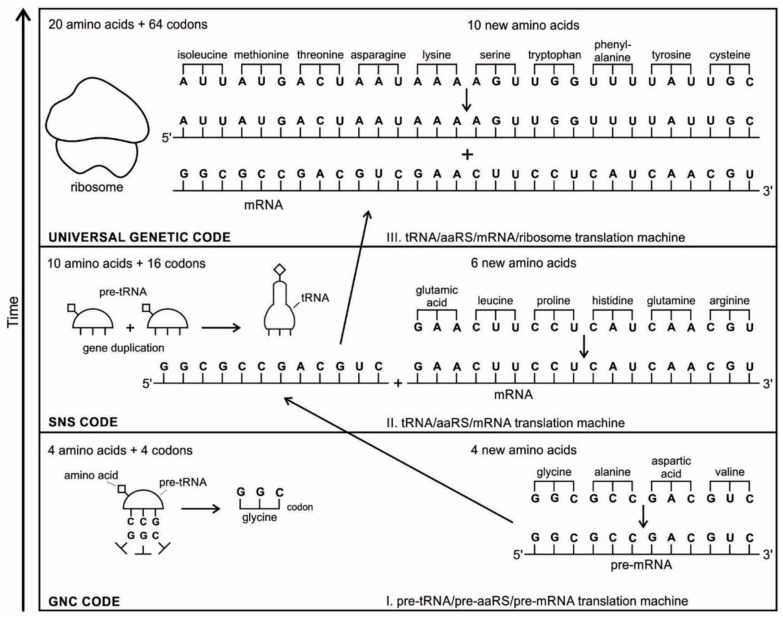
Three stages of the evolution of the genetic code corresponding to the evolution of the translation machines and the progressive addition of amino acids. Pre-tRNA molecule creates its custom-made pre-mRNA for storage of limited amino acid information in the beginning. Primitive translation process began with interaction between pre-mRNA and pre-tRNA. Pre-tRNA molecule in collaboration with pre-aaRS enzyme serve as crucial role in selecting and matching prebiotic amino acids from the prebiotic soup. At this stage, translation machine is simple, consisting of pre-tRNA/pre-aaRS/pre-mRNA. In the abiotic stage, the primitive GNC code appeared, which code four amino acids: valine, alanine, aspartic acid, and glycine [87,88]]. In the next stage, translation machine becomes modified and efficient with the evolution of the tRNA/aaRS/mRNA translation machine, when six new amino acids–glutamic acid, leucine, proline, and histidine were created. mRNA strand becomes more elongated and containing 16 codons and combination thereof with assignments of 10 amino acids with the emergence of the SNS code [87,88]. These 10 amino acids were readily available from the prebiotic environment [93]. Here, we see the beginning of degeneracy, where some the amino acids have more than one codon assignment. With the appearance of ribosome, the SNS code is modified to universal genetic code with 64 codons and 20 amino acids. The translation machine containing tRNA/aaRS/mRNA/ribosome becomes more robust with extensive degeneracy minimizing translation errors and mutation. Furthermore, amino acids with similar chemical properties seem to share similar codons. Ten more new amino acids were recruited at this stage from SNS stage: isoleucine, methionine, threonine, asparagine, lysine, serine, tryptophan, phenylalanine, tyrosine, and cysteine, totaling 20 amino acids. These new amino acids are derivatives of the first set of 10 primitive amino acids [86]. mRNA becomes independent storage device, and it can create its own strand by replication without the assistance of tRNA. mRNA strand becomes more elongated, containing information of 20 amino acids using 64 codons or combination thereof.

Here, we hypothesize that the genetic code evolved as pathways for synthesis of new amino acids became available with the progressive refinement of the translation machine. In the primitive translation machine, a symbiotic relationship is established among three components: pre-tRNA, pre-aaRS, and pre-mRNA, to create a short chain of amino acids, which form the biosynthetic protein. The protein chain grew through the addition of further residues of amino acids in the same manner. The result was a synthesis of the first protein, through the linking of the amino acids that were carried by the pre-tRNAs. At this stage of the GNC code, the translation machine began to form (Table 1, Figure 9). There are four codons in the GNC code that are assigned to four amino acids: valine, alanine, aspartic acid, and glycine, from which the first simple protein chain was created (Figure 9A, Figure 10).

**Figure 11:**
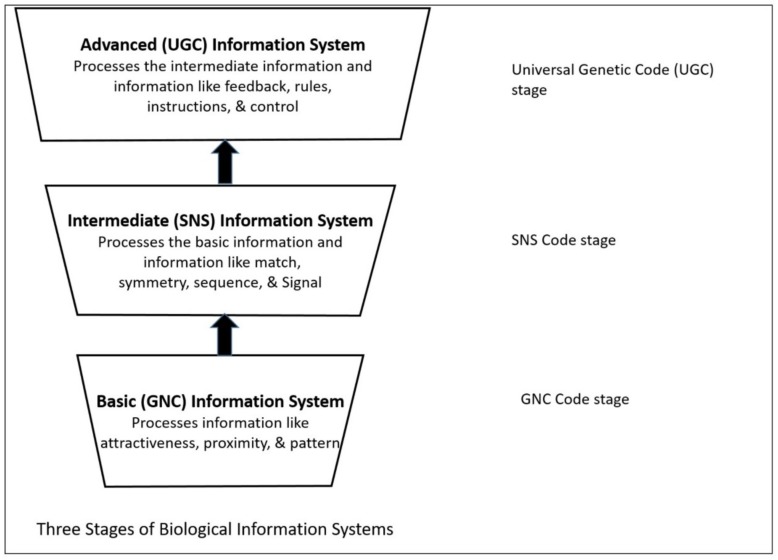
Evolution of Biological Information Systems. The basic biological system during the inception of the GNC code mainly processes the stereochemical properties of tRNA anticodons and primitive amino acids (alanine, aspartic acid, glycine, valine). The intermediate biological system during the origin of SNS code is able to process matching signals and signals etc. The advanced biological system during the origin of the universal genetic code is able to process rules, feedback, and instructions.

In the next stage of translation, pre-tRNA evolved into tRNA through gene duplication. Pre-mRNA evolved into mRNA by linking several strands of pre-mRNA to increase the storage capacity. Pre-aaRS became aaRS through ligation to specific tRNA. These three modifications gave rise to the SNS code (Figure 9B). The superior information bearing qualities of mRNA, the superior catalytic potential of aaRS, and the better adaptor capacities of tRNA emerged from such complexes with gradual expansion of the genetic code. At this stage, tRNAs selected and recruited six more amino acids (glutamic acid, leucine, proline, histidine, glutamine, and arginine), in addition to primordial amino acids (valine, alanine, aspartic acid, and glycine) (Figure 11). These charged tRNAs then create 12 additional codons through base pairing and linking pre-mRNA strands, so that the newly synthesized mRNA strands were more information-rich for storage. mRNAs now possessed at least 16 (4 +12) codons, or combinations of these codons. The mechanisms of creating new strands of pre-mRNA are similar, as shown in Figure 5. Now, two sets of pre-RNA molecules are joined to form a new generation of mRNA. At this stage, the mRNA strands became longer, containing the digital information of 16 codons representing 10 amino acids, or combination thereof allowing for redundancy. The expanded SNS code was refined through the symbiotic interactions of the tRNA/mRNA/aaRS complex. The translation system was considerably improved from the GNC to the SNS stage, but the code remains only moderately robust and susceptible to errors because of the limitation of redundancy. The primitive GNC code expanded to an SNS code composed of 16 codons (GGC, GGG, GCC, GCG, GAC, GAG, GUC, GUG, CUC, GUG, CCC, CGC, CAC, CAG, CGC, and CGG) and 10 amino acids (glycine, alanine, aspartic acid, valine, glutamic acid, leucine, proline, histidine, glutamine, and arginine) [66,80]. The first 10 amino acids, found in the prebiotic environment, have been identified in carbonaceous chondrites [4]. The SNS genetic code is an imprint of the biosynthetic relationships between amino acids (Table 1). As the code expanded, aaRS began to evolve from the earlier pre-aaRS enzyme, and then displaced their less efficient precursors. Primordial class I and class II syntheses evolved from ancestral pre-aaRS. At this point, encoded proteins are longer and they possess enough amino acid diversity to take on some of the general features of contemporary proteins. The mRNA template provides the specifications for the amino acid sequences of the protein gene products. The recipe for the biogenic protein synthesis was inscribed in the codon sequences of mRNA (Figure 9B).

The final component of the translation machine, ribosome, is enormous, a hybrid of rRNAs and r-proteins. With the participation of the ribosome, the translation machinery became more elaborate with tRNA/aaRS/mRNA/ribosome complexes; this addition enabled higher specificity in the genetic coding. The ribosome was created through the symbiosis of the rRNA and r-protein, which increased the efficiency of translation, leading to the universal genetic code with its 20 amino acids and 64 codons. (Figure 9C) (Table 1). At this stage, tRNAs selected 10 additional amino acids (isoleucine, methionine, threonine, asparagine, lysine, serine, phenylalanine, tyrosine, cysteine, and tryptophan) (Figure 11). A variety of charged tRNAs then created the corresponding codons through base pairing, forming longer strands of mRNAs. Each mRNA at this stage has the potential of accommodating 64 codons or any combination thereof. The expanded universal code was stabilized with the symbiotic interactions of the tRNA/mRNA/aaRS complex. Once the ribosome appears in the scene, the translation is considerably refined to more efficiently facilitate protein synthesis. The key chemical step of protein synthesis on ribosomes is peptidyl transfer, in which the growing nascent peptide is transferred from one tRNA molecule to the amino acid and then bound to another tRNA. Amino acids are incorporated into the growing protein on the ribosome according to the sequence of the codons of the mRNA. When a ribosome finishes reading an mRNA molecule, the two subunits split apart. The structure of the universal code is highly robust against mutational and translational errors, because of its large allowance of redundancy. Although many deviations from the universal code exist, they are limited in scope and obviously secondary, and they would be introduced later in the evolutionary process. Viruses, bacteria, fungi, plants, animals, primates, and humans all use the same code.

### 7.1. Origin of the Prebiotic Information System

The prebiotic information system evolved along with the translational machines and the genetic code. The embedded prebiotic information system became more elaborated and advanced as the translation machines became increasingly complex. The information system evolved to process different kinds of information as it coped with the changing environment. The evolution of prebiotic information system can be broadly categorized as GNC (basic), SNS (intermediate), and Universal Genetic Code (advanced) levels of information. A GNC level of biological information has more of a physical nature and it includes things like attractiveness, proximity, and pattern. An SNS level of biological information includes match, symmetry, sequence, and signal, in addition to the information at the basic level. A Universal Genetic Code level of biological information adds rules, instruction, feedback, and algorithm to its repertoire (Figure 11). As implied above, these levels of biological information are cumulative. In other words, an advanced level of biological information also includes the basic and the intermediate levels of biological information. As protocells evolved their patterns (structures), by way of environmental necessities, the structure changed to handle specialized functions. Their structural components differentiated and elaborated to handle specific roles and functions. Protocells started to have a more modular structure, where each module played a specialized role(s). Several authors have found evidence of modular structures in organelles and cells [120,121]. A module is composed of one or many types of molecules. A modular structure requires a noise-free communication among its modules, in addition to the communication within a module. This scenario uses more information than a simple non-modular structure. The information system that is used by a protocell has to coevolve to handle a greater information demand as the modular structure of the protocell becomes increasingly elaborate and specialized.

The prebiotic information systems became increasingly sophisticated in order to process more and more advanced levels of biological information. Figure 11 shows the proposed co-evolution of the biological information systems in three stages. The GNC biological information system mainly dealt with physical, structural, and spatial type of information, whereas the UGC biological information system was a sophisticated system that was capable of handling rules, feedback, and instructions, etc. in order to support the various functions of a translation process.

It is instructive to view information systems at three levels—basic (GNC), intermediate (SNS), and advanced (UGC) level, as shown in Figure 11. The basic biological information system can be compared with early man-made information systems, such as the Turing machine and the computer systems of early 1950s. The intermediate biological information system can be compared to a system that has more elaborate parts, such as memory, data storage, processor, and logic. The computer systems of the 60s and 70’s can be used as an illustration of the intermediate biological information systems. The advanced biological information system is very modular, distributed, and has a sophisticated memory structure and communication mechanism seen today in our man-made information systems that are based on embedded and distributed architectures. The pre-tRNA/pre-aaRS/pre-mRNA stage used a basic information system to process basic types of information. The tRNA/aaRS/mRNA stage used an intermediate information system to process intermediate levels information. The aaRS/tRNA/mRNA/ribosome stage used a more advanced information system that was able to process advanced types of information.

All of the signal processing devices, both analog and digital, have traits that make them susceptible to noise. Noise reduction is a goal of all communication systems. Biological information systems are of no exception. Biological processes, such as protein synthesis, undergo random fluctuations—‘noise’ or errors that are often detrimental to reliable information transfer. With the evolution of the code, denoising methods were implemented through the redundancy of codons. A practical consequence of redundancy is that errors in the third positions of the triplet codon only caused silent mutations or an error that would not affect the protein, because the hydrophilicity or hydrophobicity was maintained by the equivalent substitution of amino acids [69]. The biological information system model includes the process of translating the genetic code into corresponding amino acids as an error-prone information channel [63]. In this scenario, evolution drives the emergence of a genetic code as amino acids map that minimizes the impact of error. The codon to amino acid assignment is treated as a noisy information channel, when the mapping of codons to amino acids becomes nonrandom. The inherent noise (i.e., error in translation) in the channel poses a problem: how can a genetic code be constructed to withstand noises while accurately and efficiently translating information? The answer is redundancy: several codons can specify a single amino acid. This redundancy either implies that there is more than one tRNA for many of the amino acids or that some tRNA molecules can base pair with more than one codon. In fact, both situations occur. Redundancy explains why so many alternative codons for an amino acid differ only in their third nucleotide (Table 2). In an RNA genome, the genes and messenger are one and the same molecule, usually present in numbers of copies [68]. In such a system, innumerable mutations may take place without lethal effects. If one gene molecule and its translation product are disabled, many other unharmed molecules remain to carry on the function involved. Redundancy has been considerably increased from GNC code to SNS code and it has become extreme in the universal code for optimization, perhaps to minimize the noise or translation errors. We show a plausible correlation between stepwise modifications in the translation machinery and the evolution of the genetic code.

### 7.2. The Beginnings of the Darwinian Evolution

A key question for the origin of life is how the Darwinian evolution comes to be in the peptide/RNA world. Several mechanisms establishing the correspondence between codons/anticodons and their cognate amino acids have been suggested, either directly or via tRNAs. All of the hypotheses for the origin of life incorporate peptides as stabilizing factor, including the differences in the role that they played in code formation. In the peptide/RNA world, the peptides should have been part of the very first steps in the establishment of the genetic code [19,21,23]. Recently, Kunnev and Gospodinov [24] proposed that hybridization-induced proximity of short aminoacylated RNAs (ribozymes) led to the synthesis of peptides of random sequence. Among these, emerged a type of peptide (named bridge peptide) that was capable of interaction between specific RNAs and specific amino acids. Most likely, the ribozyme-amino acid complex would improve/stabilize a particular pair of specific mRNAs. 

Here, we accept the concept of ‘bridge peptide’ [24] in the context of the evolution of the translation machine and propose a hypothesis that could have led to the RNA-encoded protein synthesis. We suggest that aminoacylated ribozymes use amino acid as cofactor in the process of selection after stereochemical interactions between amino acids and their cognate codons/anticodons. A three-nucleotide long RNAs could be charged with a proper amino acid by an enzyme (bridge peptide) and this RNA will interact with the selected ribozyme by Watson–Crick base pairing and subsequently participate in the formation of short peptides. The selection would favor one amino acid in combination with one ribozyme and one bridge peptide that would become specific to each other. Most likely, a feedback loop from the information source (ribozyme interacting with three bases of RNA) and the stabilizing factor (bridge peptide) was established, perhaps by chance. As a result, interplay between information and its supporting structures/functions emerged. These events led to the coevolution of translation and the genetic code and also triggered the Darwinian evolution. The ribozyme would give rise to tRNA, and bridge peptide to aaRS. This transformation would result the emergence of the translation machine.

The three major events of the code evolution by translation machine—GNC, SNS, and UGC—appeared to be sequential, happening one after the other. This was the beginning of Darwinian evolution, when gradual complexity occurred and more advanced structures/functions emerged. The selection would give rise from pre-mRNA to mRNA, pre-tRNA to tRNA, pre-aaRS to aaRS, and finally the emergence of ribosome. In this evolutionary scenario, the genome size proportionally increased, covering the information for all proteins and ribozymes.

## 8. Design of Translation Machines and the Genetic Code

Life is characterized and sustained by a number of information rich biological processes that govern cellular functions and greatly contribute to its overall complexity. A biological process may involve the use of one or more modules within a cell. This involves the communication of different types of information, such as signals and connectivity etc., between and within a module [122].

### 8.1. Simulation of Translation Machines and Cells

The interest of computer scientists regarding the question of origin of life dates back to the origins of computer science. Several attempts have been made to simulate the functions of a molecular translation machine. Von Neumann pioneered the field of bio-inspired digital software/hardware [32]. His self-reproducing automata is now regarded as one of the greatest theoretical achievements in the early stages of artificial life research. He found striking parallel between artificial automata (such as a computer) and natural automata, such as various nanobots in the cells. He introduced the concept of Universal Constructor (UC), a self-constructing machine, which is capable of building any other machine, provided that it can access its description or information tape. This approach was maintained in the design of his cellular automata, which is much more than a self-replicating machine. The UC is more like a Turing machine with a tape control that could store and execute instructions. There are three components of von Neumann’s UC machine:

- a memory tape, containing the description (a one-dimensional string of elements;
- the constructor itself, a machine that is capable of reading the memory tape and interpreting its contents; and,
- a constructing arm, directed by the constructor used to build the offspring (the machine described in the memory tape).

A universal constructor with its own description could build a machine like itself. To complete the task, the universal constructor needs to copy its description and insert the copy into an offspring machine. Von Neumann noted that, if the copying machine made errors, these mutations would provide inheritable changes in the property, like the evolutionary process. He realized that the biological machine is much more sophisticated than his UC. Unlike mindless automata, which must be told exactly what to do in order to build the correct objects, a biological machine plays a dual role: it contains instructions—an algorithm—to make a certain kind of translation machine and related enzymes (e.g., mRNA, tRNA, ribosome, aaRS, and other enzymes), but additionally it can be blindly copied as a merely physical structure without reference to the instructions. Another major difference between UC and evolving natural organisms is the lack of feedback in the fitness channel of the former. Cellular automata have been useful artificial models for exploring how relatively simple rules, when combined with spatial memory, can give rise to complex emergent patterns; it may be relevant for understanding new questions regarding the cell division and its relation to information. Subsequently, Von Neumann’s UC has been modified by several workers to create Artificial life. However, von Neumann’s UC has the information system outside the machine. Accordingly, when UC reproduces its offspring, it lacks the information tape. It has to be added each time during reproduction. It is analogous to vesicle division—a mechanical division of an empty protocell, devoid of instruction.

### 8.2. Genetic Code Vs. Binary Code

Genetic code is often compared with the binary code that is used by the computers. Significant similarities and differences exist between the two types of the code. Each system has its advantages and limitations. The primary or source alphabet that is used in computers and electronic communication is the binary digit (0, 1), or a bit, a contraction for ‘binary digit’. [56]. The bit is the smallest unit of information on a computer. Binary information is grouped into sets of eight bits, which are called bytes; each byte thus has one of 2^8^ or 256 possible configurations of zeroes and ones. A byte is just eight bits and it is the smallest unit of memory that can be addressed in many computer systems.

A binary source alphabet could be extended by forming ordered pairs, ordered triplets, ordered quadruplets, and so forth to form receiving alphabets that are larger than two [56]. In molecular biology, these extensions are called codons. The simplest unit of mRNA, on the other hand, is the nucleotide, which can have one of four bases—A, U, C, and G, the quaternary ‘bit’ [123]. However, we think that the use of the word bit in a quaternary system of mRNA is a misnomer. Here, we choose a new name in the genetic code, called qit, or quaternary digit instead of bit. The quits are A, U, C, and G. This increased variation means that each nucleotide of mRNA can hold twice as much information as each digit of a binary program. The qit creates more algorithmic randomness than bit and it is more information rich. Shannon’s great insight in information theory is entropy. Entropy measures the degree of uncertainty or randomness in a system. Entropy is the opposite of information. It destroys information. We can reduce the entropy to the point where the stored information becomes maximal and transmission is highly reliable. Genetic information is low in entropy and high in information content. Entropy measures the degree of randomness that is introduced by errors. This is why the genetic codes is evolved in three stages to incrementally minimize the errors during the translation.

In mRNA, the genetic information comes in triplets of nucleotides or codons, which represent different amino acids, meaning that each codon in mRNA has only 4^3^, or 64, possibilities. Each codon is thus an extension, or ‘byte’, and it has exactly as much information as a six-bit byte, or in computer terminology a code word, since 2^6^ is 64 possible sequences for codons. Here, we use a new terminology for representing genetic information, called ‘qyte’ instead of ‘byte’. In our terminology, each qyte is three-qit long, giving 4^3^ possibilities.

Both binary and genetic codes contain signals that indicate where to begin and end the reading of their messages. Computers use start and stop bits for this purpose, while the genetic code contains one start codon and three stop codons. In a binary code, a single inaccurate bit causes its byte to have a different value, which can cause significant errors. However, mRNA exhibits greater flexibility and it is more resilient in comparison, as many nucleotide changes do not result in changes to the value of the amino acids that are coded by a codon.

However, information that is contained in life exists in two forms, digital (genetic) and analog (metabolism), and both appeared concurrently in the peptide/RNA world [48]. Digital information is encoded in linear polymers, such as DNA and RNA in discrete codons, analog information is manifest in the differing concentrations of biomolecules, especially proteins that get passed from generation to generation. Analog information systems dominate in the early prebiotic stages, but digital information systems dominate the information age [6]. Recognizing that there are two sequential events, first the origin of an analog chemical system that is capable of adaptive evolution and then a digital revolution, the origin of life problem becomes much more tractable [124]. Acceptance of this dichotomy and this progression helps to resolve the question of dual roles of RNA and peptides in generating information systems.

### 8.3. Conversion of Three Letter Codons into Numerical Codons

The genetic code is obviously not the result of a random assignment of codons to amino acids. It has a structure; the synonyms are grouped. The language-based terminology of the genetic code reflects the fact that both genes and proteins are essentially one-dimensional arrays of chemical letters. The nucleic acid alphabet comprises of four chemical letters, A, U, C, and G, whereas proteins are built from twenty different amino acids, represented by 20 abbreviated letters. In order to better visualize the codon distribution in the universal genetic code table, we substitute nucleobase alphabets of mRNA with numbers, as follows: 1 for U, 2 for C, 3 for A, and 4 for G. [In case of DNA codons, 1 represents thymine (T).] We have now created a universal numerical codon matrix in a structural format consisting of 64 numerical codons that specify 20 amino acids, and the start and stop codons (Table 2).

In Table 3, the abbreviation of the universal genetic code table is shown in numerical codons with redundancy. Each matrix cell displays information in numerical codon and its corresponding amino acid. Because of numerical distribution of codons in rows and columns, one can easily visualize the distribution of codons and their redundancy in the matrix cells; it was less obvious in standard genetic code using combinations of four letters. Looking at Table 3, we can say that codons beginning with 4 formed first, followed by codons with 2. Codons with prefix 1 and 3 were added last at the genetic code table.

In Table 4, we have shown a one-letter abbreviation of 20 amino acids, and its corresponding numerical codons. We have used three additional letters, J, X, and Z (shown in bold font) to signify three stop codons, namely opal, ochre, and amber, respectively.

Using these three tables as guides, we have developed software to simulate the translation of the numerical codon sequence of mRNAs to produce its corresponding amino acid sequence. We name this software ‘**C**odon-**A**mino Acid-**T**ranslator-**I**mitator’ or (CATI) that mimics the process of reading a sequence of codons and translating it into a sequence of the corresponding amino acids and vice versa. The CATI software can also handle the reverse process, where a sequence of amino acids is translated into a sequence of corresponding codons.

Table 5 shows some sample outputs of CATI. Table 5 is made up of several sections. In the first section, column one shows a given set of numerical codon sequences (read from a spreadsheet). Column 2 shows the corresponding amino acid sequences. Table 5 also shows the translation of randomly generated numerical codons. The second section of Table 5 shows the translation of randomly generated numerical codons. Table 5 also shows the output of the reverse process—translating a given sequence of amino acids into the corresponding sequence of numerical codons. The third, fourth, and fifth sections of Table 5 show the translation of given amino sequences into the corresponding numerical codon sequences. Since several possible codon sequences can form a given amino-sequence, we show the count of all possible codon sequences and just a few actual codon sequences in the table. The last section in Table 5 shows the conversion of DNA codon sequences into the corresponding numerical codon sequences. While using the distribution of numerical codons, we can visualize at least some of the steps by which nature might have invented the code.

CATI accepts inputs two ways—from an excel spreadsheet and from a set of randomly generated sequences. A user can create a set of numerical codon sequences while using a spreadsheet. CATI can also generate a random sequence of numerical codons of an arbitrary length for translation.

Application potential carries great potential for numerical codons in bioinformatics. For example, it can be used in translating codon sequences in DNA sequencing in numerical forms, which is the process of determining the precise order of nucleotide bases within a DNA molecule. The simultaneous quantification of mRNA and protein in a single translation process highlights the increasing importance of numerical codons in various analysis tools. During protein synthesis, we do not have to translate nucleotide language to amino acid languages. Both nucleotides and amino acids can be expressed in numerical formats. The advent of rapid DNA sequencing methods has greatly accelerated biological and medical research and discovery. The use of numbers rather than letters may expedite similarity searches between two strands of DNA. Thus, numerical codons can be used for the DNA barcoding of a species, or DNA profiling of a person as a parallel system. The Genome Sequence Data Base (GSBD), operated by the National Center for Genome Resources (NCGR), is a national database of publicly available nucleotide sequences and associated biological and bibliographic annotation. As a pilot study, the data of a small gene can be converted to numerical codon sequences by our CATI software, for a feasibility study to see whether it affords better DNA mining, alignment of two DNA sequences, and for searching methods, storage, and data retrieval systems in the future.

CATI uses numerical codes to represent and manipulate codon sequences. This enables CATI to provide better performance in terms of speed and memory when compared to most of the other software when it comes to processing large sequences of codons and amino acids. This allows for us to undertake faster translation and sequence alignments. The randomly generated numerical codons can also be subjected to some constraints during the sequence generation in order to have a desirable amino acid content.

CATI, when fully developed and implemented, can help to perform various types of analysis of codon and amino acid sequences. It can also help to identify similarity between two or more sequences of DNA. We envision CATI as an effective tool in analyzing and synthesizing non-coding as well as coding mRNAs under different constraints and conditions and performing various types of sequence alignments. In the computer, manipulation numbers have advantages over the manipulation of letters. For example, with an appropriate internal representation of numbers, bit operations can be performed giving a higher speed of computation. We believe that CATI is advantageous over many multiple DNA-protein or RNA-protein translation tools that are available online, which are based on the manipulation of letters. DNA has the potential to provide large-capacity information storage. CATI may provide new insight for developing the storage and retrieval of large data sets in DNA in the future. We have not developed this idea in this paper, and but in a subsequent paper, we want to explore the application of CATI in various translation algorithms.

**Figure 12:**
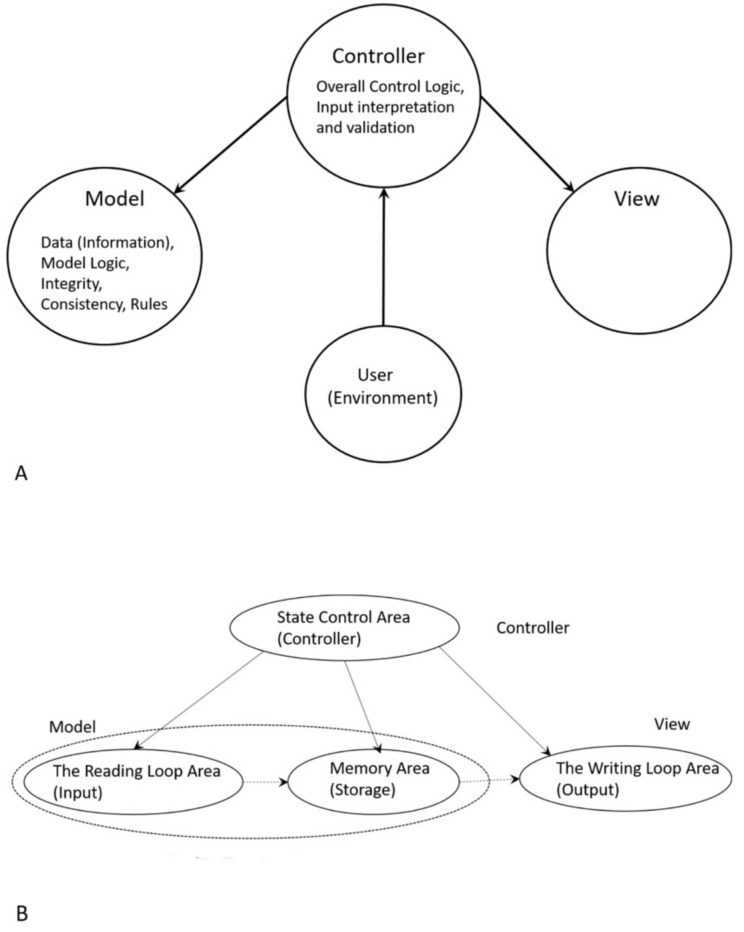
(**A**), a Model-View-Controller (MVC) Architecture pattern, and (**B**), an implementation Architecture of von Neumann’s Universal Constructor (UC). The solid arrows in the Figure show the flow of control among the components. For example, the solid arrow between the controller and model implies that the controller directs the actions of the model. A dotted arrow indicates a flow of data (information).

The CATI software is based on the Model View Controller (MVC) design pattern [33,125]. The MVC facilitates the design idea by segregating functional/task responsibilities and assigning them to different components/modules of software. This leads to an architecture with components that are relatively independent of each other. The three major components are (Figure 12A):

- The Model is the central component of the pattern. It manages the data (information), its associated logic, and the rules of application.
- The View is a (visual) representation of the model, the user interface. It relates to the logic (code) that produces the output. A view can be a form of any output representation of information.
- The Controller accepts input and acts a monitor to mediate (i.e., coordinate) between the tasks of view and model. It handles events that are generated by the user and communicates those changes to the Model, which updates its state accordingly and communicates any changes back to the Controller. The Controller then updates the view to reflect those changes.

These three architectural components of the system enable us to imitate the biological information process in a flexible and modular way. It is important to point out that the MVC design pattern is very similar to the implementation architecture of John von Neumann’s Universal Constructor of the self-reproducing automata. Given a description, the Universal Constructor theoretically produces any automata from available parts. The universal constructor has not yet been manufactured physically. However, several attempts have been made to computationally implement the universal constructor. A very good implementation of John von Neumann’s self-reproducing UC machine has been recently developed [126]. The overall architecture of this implementation is shown in Figure 12B. It has four components—the State Control Area, the Reading Loop Area, the Memory Area, and the Writing Loop Area. The State Control Area is the overall coordinator of all the other components. The Reading Loop Area reads the tape information and temporarily stores them in the memory area. The information in the memory area is used by the Writing Loop Area to produce the output. We suggest that there is a very close similarity between the Universal Constructor’s architecture, as shown in Figure 12B and the MVC Design Pattern. The Controller of the MVC design pattern corresponds to the State Control Area, the Model corresponds to the combination of the Reading Loop Area and the Memory Area, and the View corresponds to the Wring Loop Area. In fact, the MVC design pattern can be interpreted as a more modern operationalization of John von Neumann’s Universal Constructor.

We have found that the MVC design pattern is very good at abstracting the natural protein translation machine into an architecture that is modular and neatly separates the various aspects of information processing into three roles—model, view, and controller.

### 8.4. Algorithmic Design of CATI

**Figure 13:**
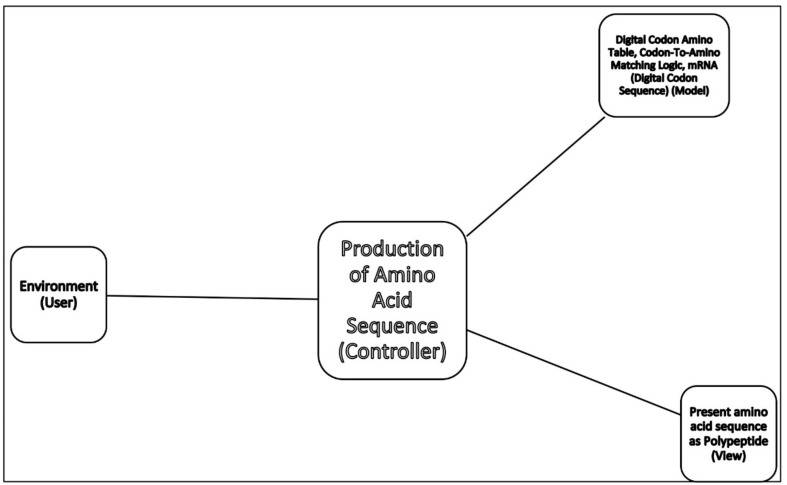
An overall architecture of CATI based on the MVC pattern. It shows the controller, model, and view aspects of the logic.

We now present the algorithm that is used by CATI. For simplicity, the algorithm shows the overall logic without dividing it into the MVC components that are shown in Figure 13. CATI uses a codon chart in the form of a codon-amino mapping shown in Table 5. CATI is given a sequence of numerical codons. CATI can also generate a random sequence numerical codon on its own. This sequence of numerical codons is then translated into the corresponding sequence of amino acids. 

**Figure 14:**
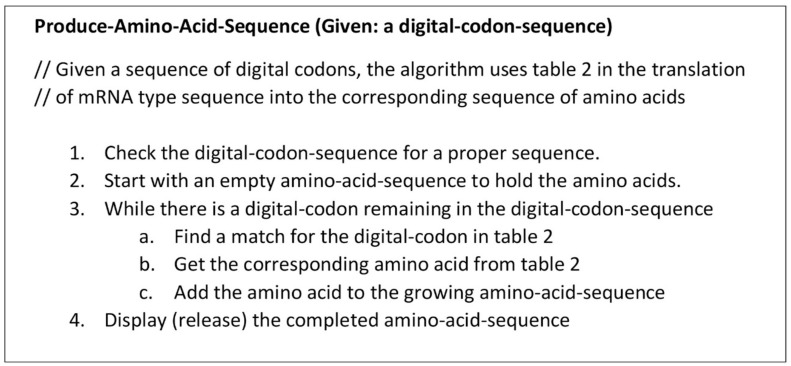
The overall Logic (Algorithm) of CATI. The logic combines the role of ribosomes, aaRS, tRNA, and mRNA into a single overall process.

Figure 14 shows the algorithm. The main step of the algorithm in Figure 14 is the third step of CATI. It uses the ideas of a pattern recognizer (step 3.a), an adapter (step 3.b), and a sequence builder (step 3.c). CATI, in essence, plays the combined role of ribosome, aaRS, and tRNA taken together.

## 9. Simulation and Visualization of the Translation Pathways

Computer simulations are useful in evolutionary biology for hypothesis testing, for verifying analytical methods, for analyzing interactions among evolutionary processes, and they are widely used in different disciplines. In general, computer simulations allow for the study of complex systems, including those analytically intractable [127]. Here, we use forward simulation models from primitive machines to advance translation machines, mimicking the biosynthetic processes for the origin of the genetic code, and for testing our hypothesis of the coevolution of the translation machines and the genetic code.

In this section, we visualize the early stages of translation machine evolution in three stages. We use a simulation and modeling software, called AnyLogic, which is commercially available (www.anylogic.com), to simulate and visualize the translation machines. We simulate translation machines at three levels of evolution:

- pre-tRNA/pre-aaRS/pre-mRNA
- tRNA/aaRS/mRNA
- tRNA/aaRS/mRNA/ribosome

Although the molecular organization of the genetic code is now known in detail, how the code came into being has not been satisfactorily addressed. We have already discussed the coevolution of translation machines and the genetic code in chapter 7; this offers a simple relation between the codon reading efficiency and the accuracy of the codon translation machine. Here, we highlight some of the features of this coevolution for visualization. The rapid and accurate translation of genetic code into proteins is the hallmark of the *information* stage; it evolved in three distinct stages through the availability of amino acids and the improvement of the translation machine. It is now widely accepted that the earliest genetic code did not encode all 20 amino acids that were found in the universal genetic code, as some amino acids have complex biochemical pathways and were probably not available in the prebiotic environment. Therefore, the genetic code evolved as pathways for the synthesis of new amino acids became available [73,92,102]. In our view, the code evolved in step with the amino acid biochemistry and the refinement of the translation machine (Figure 11). 

Currently, the simulation simply visualizes the translation process without any provision for parameter changes. In the future, we plan to parameterize the simulation of the translation processes and its visualization.

### 9.1. Stage I. Visualization—pre-aaRS-pre-tRNA-pre-mRNA Machinery

**Figure 15:**
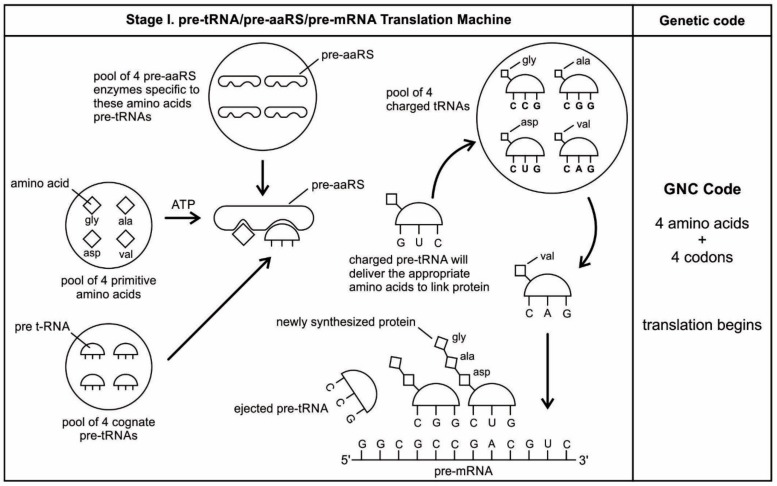
The pre-aaRS/pre-tRNA/pre-mRNA translation machine. Pre-aaRS is the matchmaker between pre-tRNA and amino acid. Four primitive amino acids and their cognate four pre-tRNAs and pre-aaRS molecules were selected from the prebiotic soup. Each amino acid with its specific pre-tRNA molecules was catalyzed by pre-aaRS enzyme in the presence of ATP to create a charged pre-tRNA molecule. In a similar way, four charged molecules were available to decode the short string mRNA one at a time. During the hybridization of anticodon of pre-tRNA with codon of pre-mRNA, each pre-tRNA delivers the appropriate amino acid, which is linked to form a chain of biosynthetic protein for the first time, containing four amino acids. This is the first stage of translation, when primitive GNC code evolves.

The first information system emerged in the prebiotic world as a primordial version of the translation machine and the genetic code. The most primitive translation machine consists of pre-aaRS/pre-tRNA/Pre-mRNA molecules. Four primordial amino acids were specific to four pre-tRNAs, and four pre-aaRS enzymes began to translate the genetic information from pre-mRNA, and to synthesize short polymer chains of protein (Figure 15). The code that evolved at this stage was the primitive GNC code [88,115], involving four codons (GGC, GCC, GAC, and GUC), which created four primordial amino acids (glycine, alanine, aspartic acid, and valine). Since there was no redundancy at this stage, the translation errors are high. 

**Figure 16:**
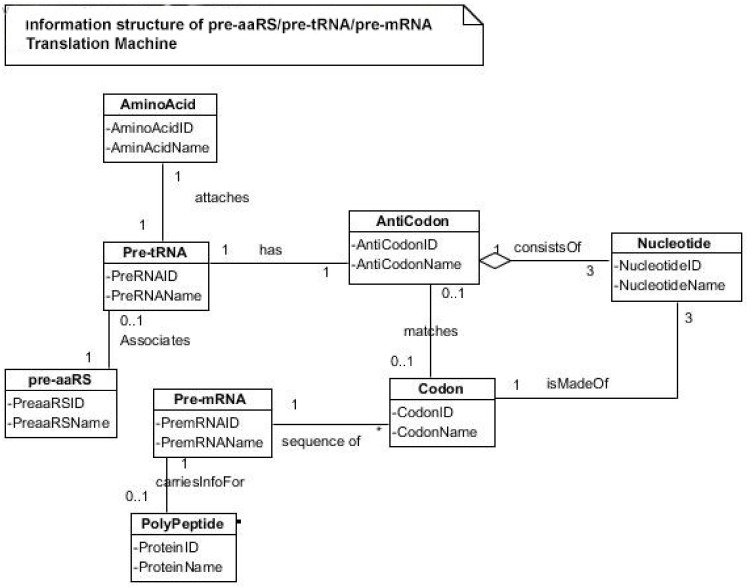
A class diagram showing an information structure during the first stage of translation system. This diagram shows relationships among various parts of the primitive translation machine. Pre-tRNA attaches to a specific amino acid with help of pre-aaRS molecules. The charged pre-tRNA molecule has an anticodon that hybridizes with the corresponding codon of pre-mRNA. As pre-tRNA begins to decode pre-mRNA molecules, short chain of protein is synthesized for the first time in a prebiotic environment. The linkage of an amino acid to a pre-tRNA established the primitive GNC genetic code.

The informational associations among these biomolecules are shown in the form of an information structure. This information structure showing macromolecules and their association with each other can be captured in the form of a class diagram (Figure 16). In a class diagram, a rectangular shape represents an informational object. The solid lines connecting these objects reflect an associative relationship between the objects. It is important to note that only a few attributes of each object are shown in the class diagrams. This is for illustration purposes only. Here, our main focus is on the interaction among the objects and not on providing a comprehensive list of attributes for each object. Figure 16 shows the information structure during the first stage of the genetic code, the GNC code. Pre-tRNA, pre-AARS, pre-mRNA, amino acid, codon, anticodon, as well as protein, and nucleotide are shown as objects in this Figure. The relationships that are shown in a class diagram are static, structural, and associative. A class diagram does not show any dynamic or temporal relationship.

**Figure 17:**
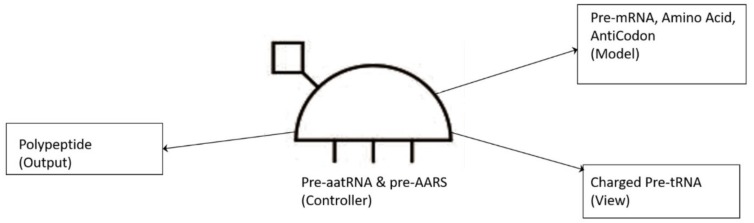
An MVC architecture of a pre-aaRS/pre-tRNA/pre-mRNA machine. Pre-aaRS and pre-tRNA direct charged pre-tRNA that will decode pre-mRNA to a growing protein.

A pre-AARS attaches the appropriate amino acid to its pre-RNA with the correct anticodon. A pre-mRNA has a sequence of codons. These are generally short-length sequences that deal with the GNC genetic codes. An amino acid carrying pre-RNA was able to base pair with codons in a pre-mRNA and helped to produce a protein as per the information in the pre-mRNA. At this stage, the types of information used are generally in the form of attractiveness, proximity, and pattern. The right combination of a catalyst, information, and the material acts as a translation machine to produce a new biological artifact. A pre-aaRS/pre-tRNA/pre-mRNA machine’s MVC architecture shows a collaboration among these three machine parts that control the formation of a biosynthetic protein chain (Figure 17). The controller uses pre-mRNA, amino acid, anticodon, and other parts of the translation machine as information to translate (convert) a codon into the corresponding amino acid with the help of a charged pre-tRNA that acts as an adaptor. The charged pre-tRNA is shown as a view. It produces the amino acid, as an output, based upon its anticodon matching with the codon in pre-mRNA. These amino acids become part of a sequence in the form of a protein chain.

A visualization of the stage I translation machine has been created using Anylogic software. Appendix A provides instructions on how to run the visualization model in the AnyLogic cloud. The visualization model shows the overall translation process regarding how various molecules dynamically interact to produce a protein.

### 9.2. Stage II. Visualization—aaRS-tRNA-mRNA Machinery

**Figure 18:**
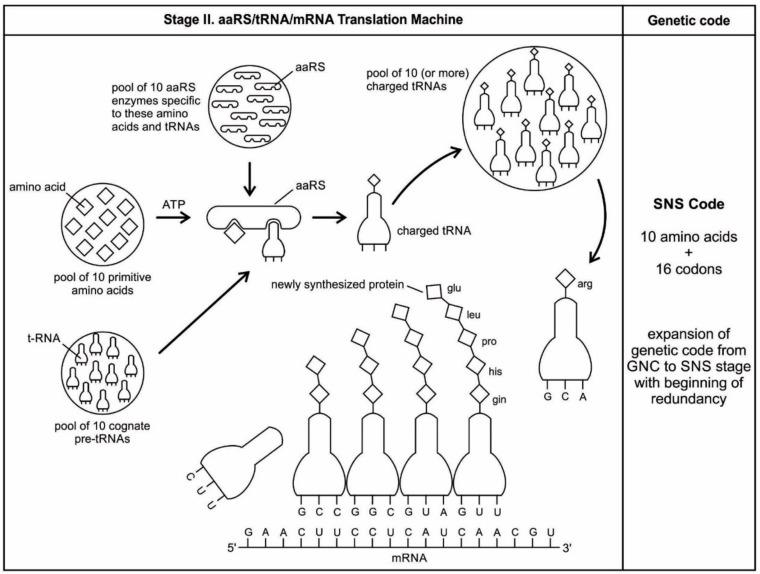
The aaRS/tRNA/mRNA translation machine. Ten primitive amino acids joined with specific tRNA molecules by aaRS enzymes to form a pool of 10 charged tRNA molecules. These charged tRNA molecules begin to decode mRNA, creating a chain of longer, biosynthesized protein molecule. At this stage, SNC code appears with 10 amino acids for 16 codons. The translation is moderately efficient with the appearance of redundancy to minimize the translation errors.

The translation machine is refined to the second stage (Figure 18) with the development of the aaRS/tRNA/mRNA machine, which increases the efficiency and decreases translation errors. At this stage, the GNC code evolved into transitional SNS code with 16 codons (GGC, GGG, GCC, GCG, GAC, GAG, GUC, GUG, CUC, GUG, CCC, CGC, CAC, CAG, CGC, and CGG), which code 10 amino acids (glycine, alanine, aspartic acid, valine, glutamic acid, leucine, proline, histidine, glutamine, and arginine) [88,115]. Because of the redundancy of codons, the translation error is minimized. 

**Figure 19:**
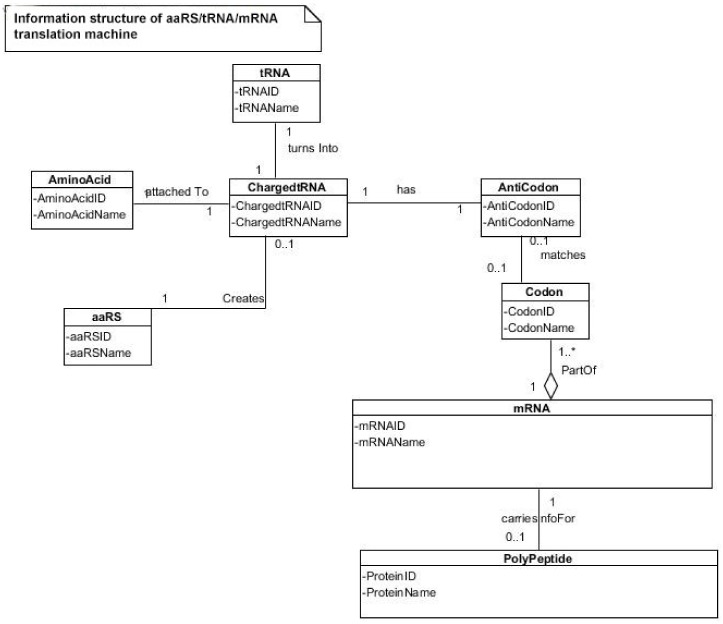
A class diagram showing the interactions of aaRS/tRNA/mRNA translation machine showing the information structure during the origin of the SNS code. At this stage, 10 primitive amino acids are available to create 10 or more charged tRNAs for decoding mRNA. An amino acid in the charged tRNA will be incorporated into a growing protein chain, at a position that is dictated by the anticodon of the tRNA.

Figure 19 shows the information structure of the aaRS/tRNA/mRNA translation machine during the second stage of the universal genetic code. At this stage, additional information in the form of match, symmetry, and sequence are also available. A tRNA is transformed into a charged tRNA by the aaRS, as shown in Figure 19. The anticodon of the charged tRNA matches with the corresponding codon in mRNA. A protein chain is formed by the decoding of mRNA by tRNA. 

**Figure 20:**
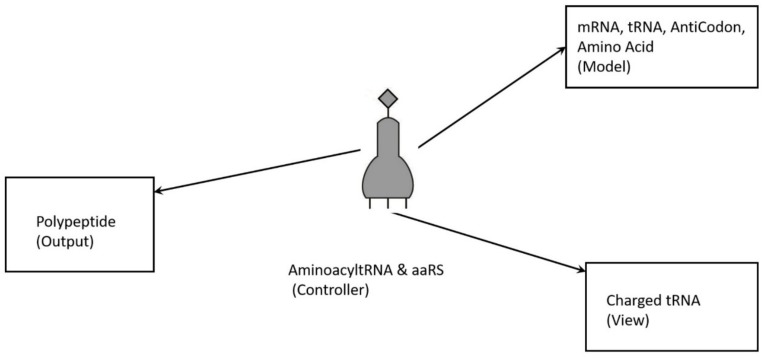
An MVC architecture of aaRS/tRNA/mRNA translation machine. aaRS and tRNA facilitate the interaction between a charged tRNA and a mRNA. A charged tRNA is an adaptor that acts as a view and helps to release the amino acid to form a chain of protein.

Using the MVC framework, we suggest that, in the case of an aaRS machine, proteins represent the ‘output’, the corresponding charged tRNAs and amino acid ligation (aa-tRNA) as a ‘view’, and a combination of aminoacyl tRNA and aaRS as a ‘controller’, and mRNA as a ‘model’ that holds codons as information (Figure 20). The directional arrows represent the control and ‘communication’ between the various parts of the machine. For example, an aaRS coordinates and facilitates the activities of mRNA, tRNA, and amino acid ligation in the formation of protein chain. The aaRS acts as a facilitator and helps to produce (select) the amino acid that matches with the codon in mRNA. Briefly, the overall logic of the second stage machine is as follows: specific tRNA binds with a particular amino acid, tRNA, and then incorporates the amino acid into a growing protein at a position is that determined by the anticodon, the anticodon matches with a codon in mRNA, and the codon acts as an information carrier that matches with the specific tRNA. The final result is the release of the linked amino acids, which are the protein chain. 

In the Appendix A, we show the instructions on how to run the visualization model for the second stage of translation machine. 

### 9.3. Stage III. Visualization—aaRS-tRNA-mRNA-Ribosome Machine Complex

**Figure 21:**
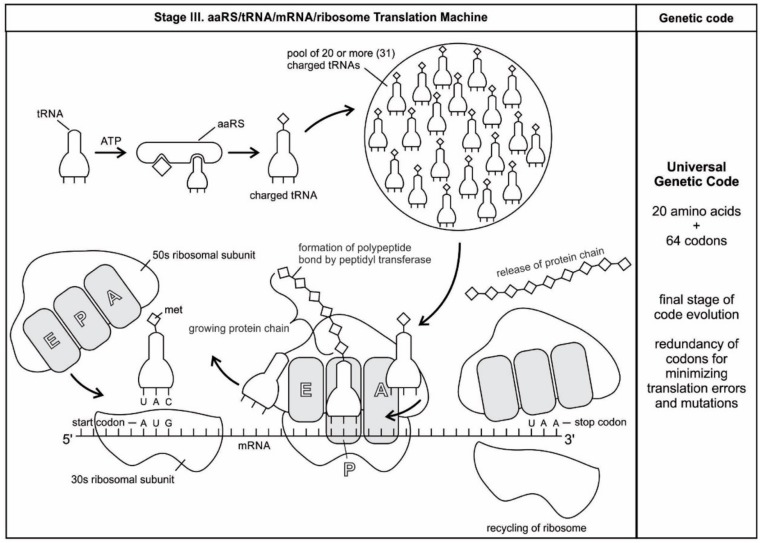
The aaRS/tRNA/mRNA/ribosome translation machine. tRNA delivers amino acid to ribosome that serves as the site of protein synthesis. Each ribosome has a large 50S subunit and a small 30S subunit that join together at the beginning of decoding of mRNA to synthesize a protein chain from amino acids carried by a tRNA. The correct tRNA enters the A site of the ribosome and appropriate amino acid is incorporated into the growing peptide chain, which transfers from tRNA in the P site to the tRNA of A site. As the ribosome moves, both tRNAs and mRNA then shit to the E site. Each newly translated amino acid is then added to a growing protein chain until ribosome completes the protein synthesis. At this stage, universal genetic code is optimized with 20 amino acids for 64 codons, including start and stop codons. The translation is highly efficient with start and stop codons; redundancy minimizes the translation errors and mutations.

By the third stage, the translation machine has fully evolved, now consisting of the aaRS/tRNA/mRNA/ribosome machine that brings forth the universal genetic code (Figure 21). The translation of the universal genetic code into protein by ribosomes requires precise mRNA decoding by tRNA. At this stage, ribosomes emerged to facilitate high-fidelity translation. About 31 tRNAs and 20 aaRS enzymes assigned 64 codons specifying 20 amino acids. Of these 64 codons, 61 represent amino acids and three are start and stop signals. Although each codon is specific to only one amino acid, the code is degenerate, because a single amino acid may be coded for more than one codon. The redundancy of the universal genetic code optimized translation errors and mutations [108]. Codons for the same amino acids tended to bundle together. Perhaps the organization of the amino acids with particular sequences of the code minimized the errors that crept into the proteins. Among the 20 amino acids in the universal code, approximately half came from the prebiotic soup; as we see in the SNS code, the remaining half of amino acids were derivatives of the first set of 10 primitive amino acids by biosynthesis [78].

**Figure 22:**
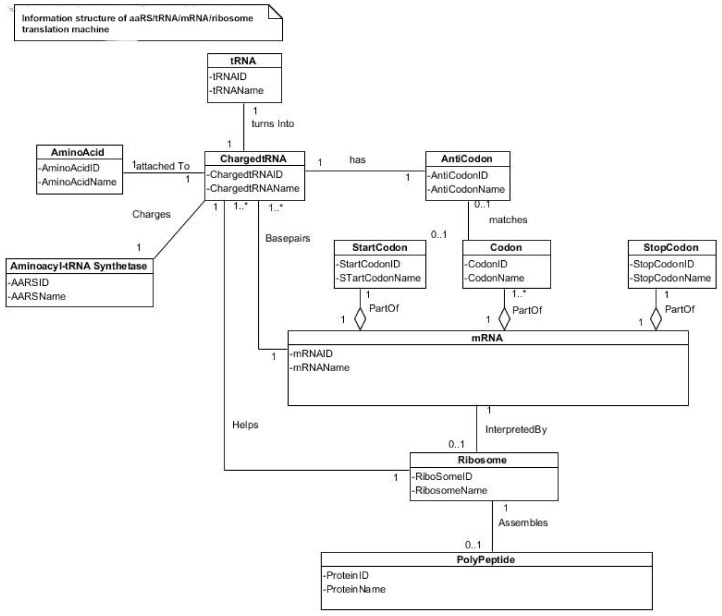
Class diagram showing the interactions of aaRS/tRNA/mRNA/ribosome translation machine. The diagram is similar to that of Figure 22. In addition, it shows the introduction of a ribosome, which decodes mRNA with the help of charged tRNA.

Figure 22 shows the information structure that was available during the third stage of the genetic code. During this stage, a ribosome uses rules, and feedback types of information, in addition to the other types of information during translation. A ribosome acts like a biological assembly machine in the translation of mRNA into protein. A ribosome performs the protein synthesis with the assistance of two other kinds of molecules—mRNA and tRNA.

**Figure 23:**
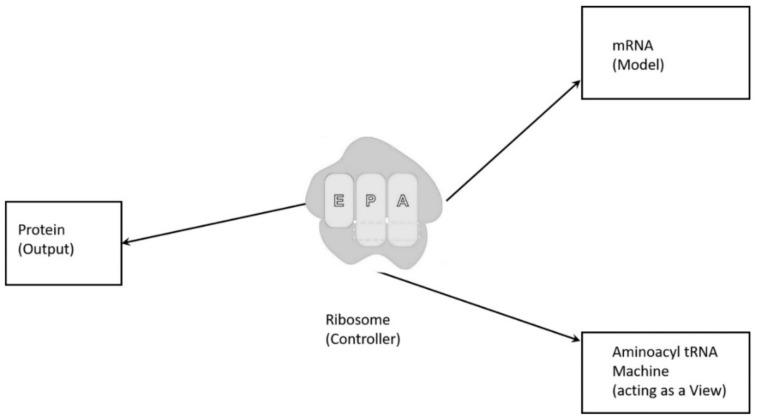
An MVC structure of aaRS/tRNA/mRNA/ribosome translation machine. A ribosome is a part of a bigger machine that uses the aaRS machine to decode the mRNA information into a corresponding sequence of amino acids to link a protein chain.

Figure 23 shows an MVC model of the ribosome machine. A ribosome machine is sometimes equated with a factory with several machines. It uses other machines, such as an aaRS machine, to complete the translation process. A ribosome plays the role of the controller. mRNA is a model containing the information in the form of a sequence of codons. An aa-tRNA machine plays the role of a view that supplies an amino acid. Note that this machine is depicted in Figure 21. Here, the ribosome machine uses the aa-tRNA machine as its submachine (sub part), signifying a functional hierarchy among macromolecules. The ribosome produces the peptide chain (protein) by establishing the proper match (fit) between an aa-tRNA and the corresponding codon in the mRNA.

In the translation process of the third stage, the translation machine, with the assistance of a ribosome, is visualized using an AnyLogic model. We show the instructions in the Appendix A how to run the third stage of the visualization model.

## 10. Discussion and Conclusions

Although the origin of the prebiotic information is not fully understood, the manufacturing processes of different species of RNAs and proteins by molecular machines in the peptide/RNA world require not only physical quantities, but also additional entities, like sequences and coding rules. The demand for a wide range of specific enzymes to catalyze complex prebiotic chemistry was the prime selective pressure for the origin of the information systems for creating programmed protein synthesis. These coded proteins are specific and quite different from the random peptides that are generated by linking amino acids in the vent environment. There is a great potential in the application of numerical codons in bioinformatics, such as barcoding, DNA mining, or DNA fingerprinting. 

We have reviewed the bottom-up pathways of prebiotic synthesis that address several hallmarks in living systems, such as the encapsulation and protocell division, peptide/RNA world, origin of mRNA, origin of aaRS, information processing, energy transduction, and adaptability. The scenarios for the origin of the translation machinery and the genetic code that are outlined here are both sketchy and speculative, but follow those biosynthetic pathways. It is the informational role of RNAs, aided by a series of enzymes, which is key to transforming nonliving chemistry into translation machines and the genetic code. 

There are several novel ideas in the origin of prebiotic information that are presented in this paper:

1. The peptide/RNA world was more parsimonious in the vent environment than the popular RNA world hypothesis. It is easier to make proteins than RNAs in the vent environment. The duality of replication and metabolism is the intrinsic property of life and it must have appeared simultaneously before the origin of the first cells. Both RNAs and proteins worked in tandem to jumpstart the life assembly.
2. The *Information* stage is a crucial step in the origin of life prior to the origin of DNA and the first cell. We emphasize that reproduction is not possible without information. Life is information stored in a symbiotic genetic language. Information is an emergent property in the peptide/RNA world. The molecular attraction between tRNA and amino acid led to the translation machinery and the genetic code. The demand for specific protein enzymes over peptides in the peptide/RNA world was the selective agent for the emergence of the *information* age.
3. Both mRNAs and proteins were invariably manufactured by molecular machines that required sequences and coding rules. The crucial step was the ligation of a specific amino acid to its corresponding pre-tRNA molecule that created a repertoire of complex machinery parts for translation. tRNA is an ancient molecule that created custom-made mRNA for the storage of amino acid assignment. During this stage, the translation and the genetic code coevolved.
4. The molecular basis of the genetic code manifests itself in the interaction of aaRSs and their cognate tRNAs. Aminoacylated ribozymes used amino acids as cofactor with the help of bridge peptides as a process for selection between amino acids and their cognate codons/anticodons; this self-sustained RNA-peptide complex may trigger proto-translation. As bridge peptide evolved to pre-aaRS and ribozyme to pre-tRNA, many of their structures were modified, but their functions were continued and elaborated. Eventually, the interaction of aaRS and tRNA was established.
5. The piecemeal buildup of translation machines consisting of tRNAs, mRNAs, aaRS, and ribosomes are proposed.
6. The existing theories on the origin and evolution of the genetic code are compatible with our coevolution model of translation machines and the genetic code. We suggest that there were three stages in the evolution of the genetic code—GNC, SNS, and finally the universal genetic code [88,115]. The code evolved through the progressive refinery of translation machines, from the pre-tRNA/pre-aaRS machine, to the tRNA/aaRS/mRNA machine, and finally the tRNA/aaRS/mRNA/ ribosome machine. This was the beginning of the Darwinian evolution that exhibited an interplay between information and its supporting structure. The evolution of the translation machine reflects the incremental enrichment of information content in the genetic bank of mRNA. An evolutionary path from bridge peptide to protozymes to urzymes to pre-aaRS to aaRS suggests increasing the complexity of functions and satisfying the rule of continuity.
7. Using a computer simulation, and a visualization model of the possible biosynthetic pathways that led to the origin of the information system, we show the step-by-step evolution of the translation machines and the genetic code.

An mRNA strand with strings of codons is a relatively small and simple molecule possessing limited storage capacity. It can store small amounts of genetic information; the capability of a ribozyme as an enzyme is severely limited. However, this catalytic deficiency is compensated by a variety of enzymes that are available in the hydrothermal vent environment. The short life of mRNA makes the protocell very responsive to changing conditions in the environment. Later, more durable DNA would emerge to become the molecule of choice for the large storage of genetic information regarding protein synthesis, replacing mRNA from its main function. The new generation of mRNA is created by the transcription of DNA. mRNA becomes a daughter of DNA to carry out its specific instruction of translation and protein synthesis.

The *information* age, with the origin of translation and the genetic code, was a watershed event in biogenesis triggering the origin of DNA and the first cells. The information age is quite distinct and more derived than the prebiotic chemical stage, and it is a necessary prelude to the biological age. However, it lacks the one crucial attribute of life: cell division. In the prebiotic information stage, each mRNA became a gene that contained the recipe for a specific protein. However, the information system would be fully developed with the appearance of DNA that contained a permanent storage for both hereditary information and the transcription capability. With the emergence of DNA, the central dogma is established; information flows from DNA to mRNA to proteins. 

The new information paradigm suggests that life is organic chemistry, plus information, plus code, plus cell division, where replication, sequencing, coding, transcription, and reproduction become important attributes. The advent of cell division defines the emergence of the first cells from their protocell precursors. Life began when a cell was capable of dividing into two identical daughter cells. A protocell in the prebiotic information age did not acquire the capability of identical cell division.

## Figures and Tables

**Table 1:**
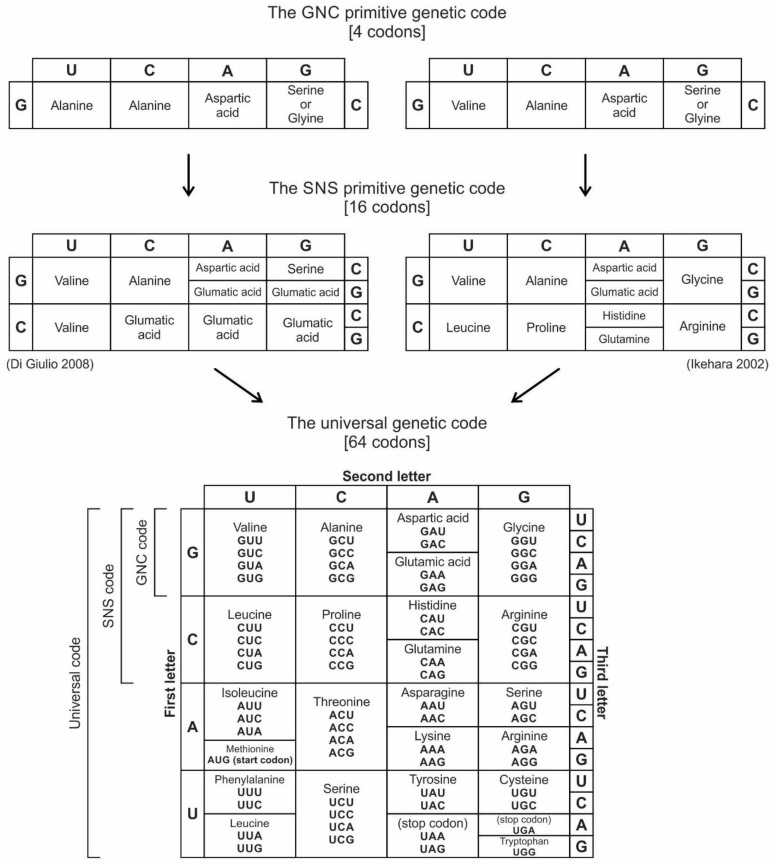
The evolution of the Universal Genetic Code is seen in three distinct stages. The nucleotide sequence of an mRNA (in a digital format) is translated into the amino acid sequence of a protein (in an analog format) via the genetic code. Genetic information is encoded in mRNA using codons, comprised of four bases: uracil (U), cytosine (C), adenine (A), and guanine (G). The Figure shows the evolutionary pathway going from a GNC code (four codons) through a SNS code (16 codons), to the Universal Genetic Code. To decode a codon, the first letter is matched in the left column, the second letter on the top row, and the third letter on the right column. The 64 codons, along with the amino acid or stop signal that they specify, are shown in the boxes. All but two of the amino acids (methionine and tryptophan) have more than one codon. Note that in the mRNA, uracil replaces the thymine found in DNA. A (after [87]), B (after [88]); C, the Universal Genetic Code. Instead of a conventional representation, the modern genetic code is shown reflecting the order of codon occurrence from GNC, to SNS, to a modern code (columns G and U inverted).**Table 2.** Universal Genetic code showing numerical codons. **Table 3.** Universal Genetic code showing numerical codons **Table 4.** 20 Primary Amino Acids in the Genetic Code and their corresponding numerical codons.

**Table 2:**
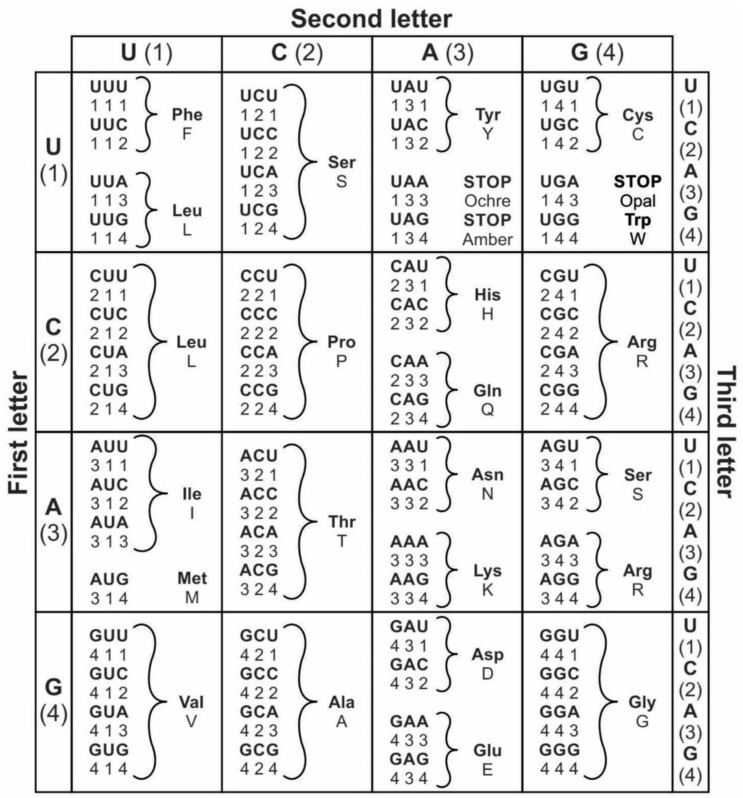
Universal Genetic code showing numerical codons.

**Table 3:**
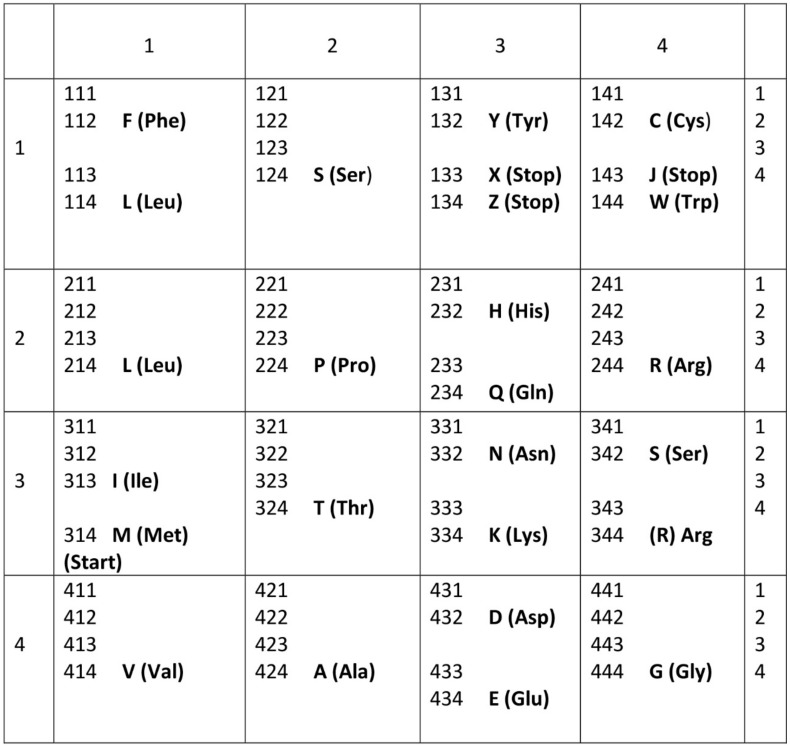
Universal Genetic code showing numerical codons.

**Table 4:** 20 Primary Amino Acids in the Genetic Code and their corresponding numerical codons.

1-Letter Abbreviation	3-Letter Abbreviation	Amino Acid	Numerical Codons
A	Ala	Alanine	421, 422, 423, 424
B	—	—	—
C	Cys	Cysteine	141, 142
D	Asp	Aspartic acid	431, 432
E	Glu	Glutamic acid	433, 434
F	Phe	Phenylalanine	111,112
G	Gly	Glycine	441, 442, 443, 444
H	His	Histidine	231, 232
I	Ile	Isoleucine	311, 312, 313
J	**Stop**	**Opal**	143
K	Lys	Lysine	333, 334
L	Leu	Leucine	113, 114, 211, 212, 213, 214
M	**Met (Start)**	**Methionine**	314
N	Asn	Asparagine	331, 332
O	—	—	—
P	Pro	Proline	221, 222, 223, 224
Q	Gln	Glutamine	233, 234
R	Arg	Arginine	241, 242, 243, 244, 343, 344
S	Ser	Serine	121, 122, 123, 124, 341, 342
T	Thr	Threonine	321, 322, 323, 324
U	—	—	—
V	Val	Valine	411, 412, 413, 414
W	Trp	Tryotophan	144
X	**Stop**	**Ochre**	133
Y	Tyr	Tyrosine	131, 132
Z	**Stop**	**Amber**	134

**Table 5:** Conversion of numerical codons into corresponding amino acids and vice versa using **C**odon-**A**mino Acid-**T**ranslator-**I**mitator (CATI) software.

**Numerical Codon Sequences**	**Corresponding Amino Sequences**
**142**343**311**141**334**	**C**R**I**C**K**
**431**424**243**144**312**332	**D**A**R**W**I**N
**433**312**332**122**324**434**313**331	**E**I**N**S**T**E**I**N
**221**131**244**422**314**313**431**	**P**Y**R**A**M**I**D**
**144**423**321**434**242**	**W**A**T**E**R**
**433**423**241**323**231**	**E**A**R**T**H**
**424**314**433**244**313**141**423**331	**A**M**E**R**I**C**A**N
**314**424**313**432**433**331**231**423**312**344	**M**A**I**D**E**N**H**A**I**R
**141**244**423**131**111**312**124**231	**C**R**A**Y**F**I**S**H
**144**423**321**434**244**321**421**331**442**433**243**313**332**433**111**244**131**	**W**A**T**E**R**T**A**N**G**E**R**I**N**E**F**R**Y**
**The Randomly Generated Numerical Codon Sequences of Up to Length: 99**	**Corresponding Amino Acid Sequences**
**314**234**241**112**241**122**214**421**123**412**142**423**124**342**442**441**142**112**423**424**411**132**124**423**224**124**334**433**133**	**M**Q**R**F**R**S**L**A**S**V**C**A**S**S**G**G**C**F**A**A**V**Y**S**A**P**S**K**E**X**
**314**121**222**221**421**224**234**214**434**212**411**221**324**323**124**114**241**234**144**132**121**412**242**334**312**113**221**222**432**111**132**243**134**	**M**S**P**P**A**P**Q**L**E**L**V**P**T**T**S**L**R**Q**W**Y**S**V**R**K**I**L**P**P**D**F**Y**R**Z**
**314**111**121**444**212**434**421**144**342**322**142**444**232**132**433**122**114**412**222**423**343**444**212**243**132**244**221**413**143**	**M**F**S**G**L**E**A**W**S**T**C**G**H**Y**E**S**L**V**P**A**R**G**L**R**Y**R**P**V**J**
**Amino Acid Sequences**	**Corresponding Numerical Codon Sequences**
**The Count of all Possible Codon Sequences for the following Amino Sequence: 221,184**	**Only the First 6 Codon Sequences Generated**
**S**D**S**Y**D**P**C**T**G**L	**342**432**342**132**432**223**142**323**443**213
**S**D**S**Y**D**P**C**T**G**L	**341**432**342**132**432**223**142**323**443**213
**S**D**S**Y**D**P**C**T**G**L	**123**432**342**132**432**223**142**323**443**213
**S**D**S**Y**D**P**C**T**G**L	**122**432**342**132**432**223**142**323**443**213
**S**D**S**Y**D**P**C**T**G**L	**124**432**342**132**432**223**142**323**443**213
**S**D**S**Y**D**P**C**T**G**L	**121**432**342**132**432**223**142**323**443**213
**The Count of all Possible Codon Sequences for the following Amino Sequence: 1.5912087619658678 × 10^41^**	**Only the First 6 Codon Sequences Generated**
**S**D**S**Y**D**P**C**T**G**L**L**Q**K**S**P**Q**C**C**N**T**D**I**L**G**V**A**N**L**D**C**H**G**P**P**S**V**P**T**S**P**S**Q**F**Q**A**S**C**V**A**D**G**G**R**S**A**R**C**C**T**L**S**L**L**G**L**A**L**V**C**T**D**P**V**G**I**	**342**432**342**132**432**223**142**323**443**213**213**233**333**342**223**233**142**142**332**323**432**313**213**443**413**423**332**213**432**142**232**443**223**223**342**413**223**323**342**223**342**233**112**233**423**342**142**413**423**432**443**443**343**342**423**343**142**142**323**213**342**213**213**443**213**423**213**413**142**323**432**223**413**443**313**
**S**D**S**Y**D**P**C**T**G**L**L**Q**K**S**P**Q**C**C**N**T**D**I**L**G**V**A**N**L**D**C**H**G**P**P**S**V**P**T**S**P**S**Q**F**Q**A**S**C**V**A**D**G**G**R**S**A**R**C**C**T**L**S**L**L**G**L**A**L**V**C**T**D**P**V**G**I**	**341432**342**132**432**223**142**323**443**213**213**233**333**342**223**233**142**142**332**323**432**313**213**443**413**423**332**213**432**142**232**443**223**223**342**413**223**323**342**223**342**233**112**233**423**342**142**413**423**432**443**443**343**342**423**343**142**142**323**213**342**213**213**443**213**423**213**413**142**323**432**223**413**443**313
**S**D**S**Y**D**P**C**T**G**L**L**Q**K**S**P**Q**C**C**N**T**D**I**L**G**V**A**N**L**D**C**H**G**P**P**S**V**P**T**S**P**S**Q**F**Q**A**S**C**V**A**D**G**G**R**S**A**R**C**C**T**L**S**L**L**G**L**A**L**V**C**T**D**P**V**G**I**	**123**432**342**132**432**223**142**323**443**213**213**233**333**342**223**233**142**142**332**323**432**313**213**443**413**423**332**213**432**142**232**443**223**223**342**413**223**323**342**223**342**233**112**233**423**342**142**413**423**432**443**443**343**342**423**343**142**142**323**213**342**213**213**443**213**423**213**413**142**323**432**223**413**443**313**
**S**D**S**Y**D**P**C**T**G**L**L**Q**K**S**P**Q**C**C**N**T**D**I**L**G**V**A**N**L**D**C**H**G**P**P**S**V**P**T**S**P**S**Q**F**Q**A**S**C**V**A**D**G**G**R**S**A**R**C**C**T**L**S**L**L**G**L**A**L**V**C**T**D**P**V**G**I**	**122**432**342**132**432**223**142**323**443**213**213**233**333**342**223**233**142**142**332**323**432**313**213**443**413**423**332**213**432**142**232**443**223**223**342**413**223**323**342**223**342**233**112**233**423**342**142**413**423**432**443**443**343**342**423**343**142**142**323**213**342**213**213**443**213**423**213**413**142**323**432**223**413**443**313**
**S**D**S**Y**D**P**C**T**G**L**L**Q**K**S**P**Q**C**C**N**T**D**I**L**G**V**A**N**L**D**C**H**G**P**P**S**V**P**T**S**P**S**Q**F**Q**A**S**C**V**A**D**G**G**R**S**A**R**C**C**T**L**S**L**L**G**L**A**L**V**C**T**D**P**V**G**I**	**124**432**342**132**432**223**142**323**443**213**213**233**333**342**223**233**142**142**332**323**432**313**213**443**413**423**332**213**432**142**232**443**223**223**342**413**223**323**342**223**342**233**112**233**423**342**142**413**423**432**443**443**343**342**423**343**142**142**323**213**342**213**213**443**213**423**213**413**142**323**432**223**413**443**313**
**S**D**S**Y**D**P**C**T**G**L**L**Q**K**S**P**Q**C**C**N**T**D**I**L**G**V**A**N**L**D**C**H**G**P**P**S**V**P**T**S**P**S**Q**F**Q**A**S**C**V**A**D**G**G**R**S**A**R**C**C**T**L**S**L**L**G**L**A**L**V**C**T**D**P**V**G**I**	**121**432**342**132**432**223**142**323**443**213**213**233**333**342**223**233**142**142**332**323**432**313**213**443**413**423**332**213**432**142**232**443**223**223**342**413**223**323**342**223**342**233**112**233**423**342**142**413**423**432**443**443**343**342**423**343**142**142**323**213**342**213**213**443**213**423**213**413**142**323**432**223**413**443**313**
**The Count of all Possible Codon Sequences for the following Amino Sequence: 3,538,944**	Only the **First 4 Codon Sequences Generated**
**D**P**C**T**G**L**L**G**L**A**V**	**432**223**142**323**443**213**213**443**213**423**413**
**D**P**C**T**G**L**L**G**L**A**V**	**431**223**142**323**443**213**213**443**213**423**413**
**D**P**C**T**G**L**L**G**L**A**V**	**432**222**142**323**443**213**213**443**213**423**413**
**D**P**C**T**G**L**L**G**L**A**V**	**431**222**142**323**443**213**213**443**213**423**413**
**DNA Codon Sequences**	**The Corresponding Numerical Codon Sequences**
**T**G**C**A**G**A**A**T**T**T**G**T**A**A**G**	**1**4**2**3**4**3**3**1**1**1**4**1**3**3**4**
**G**A**T**G**C**G**C**G**A**T**G**G**A**T**C**A**A**C	**4**3**1**4**2**4**2**4**3**1**4**4**3**1**2**3**3**2
**G**A**A**A**T**C**A**A**C**T**C**C**A**C**G**G**A**G**A**T**A**A**A**T	**4**3**3**3**1**2**3**3**2**1**2**2**3**2**4**4**3**4**3**1**3**3**3**1
**C**C**T**T**A**T**C**G**G**G**C**C**A**T**G**A**T**A**G**A**T**	**2**2**1**1**3**1**2**4**4**4**2**2**3**1**4**3**1**3**4**3**1 **
**T**G**G**G**C**A**A**C**T**G**A**G**C**G**C**	**1**4**4**4**2**3**3**2**1**4**3**4**2**4**2**
**G**A**A**G**C**A**C**G**T**A**C**A**C**A**T**	**4**3**3**4**2**3**2**4**1**3**2**3**2**3**1**
**G**C**G**A**T**G**G**A**A**C**G**G**A**T**A**T**G**T**G**C**A**A**A**T	**4**2**4**3**1**4**4**3**3**2**4**4**3**1**3**1**4**1**4**2**3**3**3**1
**A**T**G**G**C**G**A**T**A**G**A**C**G**A**A**A**A**T**C**A**T**G**C**A**A**T**C**A**G**G	**3**1**4**4**2**4**3**1**3**4**3**2**4**3**3**3**3**1**2**3**1**4**2**3**3**1**2**3**4**4
**T**G**T**C**G**G**G**C**A**T**A**T**T**T**T**A**T**C**T**C**G**C**A**T	**1**4**1**2**4**4**4**2**3**1**3**1**1**1**1**3**1**2**1**2**4**2**3**1
**T**G**G**G**C**A**A**C**T**G**A**G**C**G**G**A**C**T**G**C**T**A**A**T**G**G**C**G**A**A**C**G**A**A**T**A**A**A**C**G**A**A**T**T**T**C**G**G**T**A**T**	**1**4**4**4**2**3**3**2**1**4**3**4**2**4**4**3**2**1**4**2**1**3**3**1**4**4**2**4**3**3**2**4**3**3**1**3**3**3**2**4**3**3**1**1**1**2**4**4**1**3**1**

**Figure A1:**
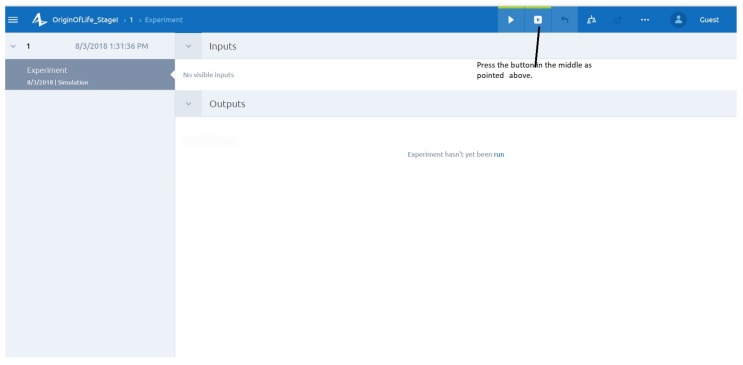
A screen shot of the web page showing the button to be pushed.

## Appendix A

This appendix is a user guide for the visualization model developed in the AnyLogic software. It describes as to how to run the visualization model for each stage of the translation machines. The visualization models are hosted on the AnyLogic cloud, a service that allows simulation models to be available online on Internet. These hosted models can be run under a browser such as Chrome, Internet Explorer, and Fire Fox, etc. In the following sections, it is assumed that an Internet browser is up and running either on a laptop or on a PC.

### Instructions for Stage I Visualization

This section shows the steps to run the stage I model. Please follow these steps:

Step 1. Paste the following URL link in your browser’s address line and go to that link: https://cloud.anylogic.com/model/5a297e73-af36-4af5-a92c-a416021a9bda?mode=DASHBOARD&experiment=82e7de3a-b6df-4ab2-9304-eeba402cc447

Step 2. You should see a web page that looks like the one shown in Figure A1.

Step 3. Press the button as indicated in Figure A1. The visualization model should start running in a few seconds.

Step 4. The model can be stopped any time by closing the webpage.

### Instructions for Stage II Visualization

This section shows the steps to run the stage II model. Please follow these steps:

Step 1. Paste the following URL link in your browser’s address line and go to that link: https://cloud.anylogic.com/model/c19bfa43-d338-4a1b-93c3-4071d738add7?mode=DASHBOARD

Please follow the steps 2 to 4 as described above in the stage I visualization.

### Instructions for Stage III Visualization

This section shows the steps to run the stage III model. Please follow these steps:

Step 1. Paste the following URL link in your browser’s address line and go to that link: https://cloud.anylogic.com/model/4c291275-7bc4-4510-8fbd-e349bb59141e?mode=DASHBOARD

Please follow the steps 2 to 4 as described above in the stage I visualization.

## Note

Please note that the visualization models are hosted in AnyLogic Cloud. After running the model, the screen can be closed by clicking on the “X” button on top right-hand side. These models have an ‘Animated Run Time Limit’ in the cloud. The model can be closed and re-run if you get a message as shown below:

“Animated run time limit

The model has run for the maximum time allowed…”

Simply close the message box, then close the model by pressing on the “X” button and run the model by pressing on the play button as shown in Stage I instructions.

## References

[1] Deamer D.W. (2012). First Life: Discovering the Connections between Stars, Cells, and How Life Began.

[2] Bernstein M.P., Sandford S.A., Allamonda L.J. (1999). Life’s fur flung raw material. Sci. Am..

[3] Deamer D.W., Dworkin J.P., Sandford S.A., Bernstein M.P., Allamandola L.J. (2002). The first cell membranes. Astrobiology.

[4] Pizzarello J.R., Cronin J.R. (2000). Non-racemic amino acids in the Murchison and Murray meteorites. Geochem. Cosmochem. Acta.

[5] Marchi S., Bottke W.F., Elkins-Tanton L.T., Bierhaus M., Wuennemann K., Morbidelli A., Kring D.A. (2014). Widespread mixing and burial of Earth’s Hadean crust by asteroid impacts. Nature.

[6] Chatterjee S., Kolb V.M. (2018). The hydrothermal impact crater lakes: The crucibles of life’s origin. Handbook of Astrobiology.

[7] Chyba C., Sagan C. (1992). Endogenous production, exogenous delivery and impact-shock synthesis of organic molecules: An inventory for the origin of life. Nature.

[8] Chatterjee S., Clarke B. (2015). The RNA/protein world and the endoprebiotic origin of life. Earth, Life, and System.

[9] Chatterjee S. (2016). A symbiotic view of the origin of life at hydrothermal impact crater lakes. Phys. Chem. Chem. Phys..

[10] Cockell C.S. (2006). The origin and emergence of life under impact bombardment. Phil. Trans. R. Soc..

[11] Osiniski G.R., Tornabene L.L., Banerjee N.R., Cockell C.S., Flemming R., Izawa M.R.M., McCutcheon J., Parnell J., Preston L.J., Pickersgill A.E. (2013). Impact-generated hydrothermal systems on Earth and Mars. Icarus.

[12] Kring D.A. (2000). Impact events and their effect on the origin, evolution, and distribution of life. GSA Today.

[13] Martin W.F., Sousa F.L., Lane N. (2014). Energy at life’s origin. Science.

[14] Boehnke P., Harrison T.M. (2016). Illusory Late Heavy Bombardments. Proc. Nat. Acad. Sci. USA.

[15] Mojzsis S.J., Harrison T.M., Pidgeon T.T. (2001). Oxygen-isotope evidence from ancient zircons for liquid water at the Earth’s surface 4300 Myr ago. Nature.

[16] Kasting J.F. (2014). Atmospheric composition of Hadean-Early Archean Earth: Theimportance of CO. Geol. Soc. Spec. Pap..

[17] Carter C.W. (2015). What RNA world? Why a peptide/RNA partnership merits renewed experimental attention. Life.

[18] Carter C.W. (2016). An alternative to the RNA world. Nat. Hist..

[19] Carter C.W., Wills P.R. (2017). Interdependence, reflexivity, impedance matching, and the evolution of genetic coding. Mol. Biol. Evol..

[20] Harish A., Caetano-Anolles G. (2001). Ribosomal history reveals origin of modern protein synthesis. PLoS ONE.

[21] Bowman J.C., Hud N.V., Williams J.D. (2015). The ribosome challenge to the RNA world. J. Mol. Evol..

[22] Hazen R.M. (2005). Genesis: The Scientific Quest for Life.

[23] Caetano-Anolles K., Caetano-Anolles G. (2016). Piecemeal buildup of the genetic code, ribosome, and genomes from primordial tRNA building blocks. Life.

[24] Kunnev D., Gospodinov A. (2018). Possible emergence of sequence specific RNA aminoacylation via peptide intermediary to initiate Darwinian evolution and code through origin of life. Life.

[25] Farias S.T., Rego T.H., Jose M.V. (2016). tRNA core hypothesis for the transition from the RNA world to the ribonucleoprotein world. Life.

[26] Lupus A.N., Alva V. (2017). Ribosomal proteins as documents of the transition from unstructured (poly)peptides to folded proteins. J. Struct. Biol..

[27] Crick F.H.C. (1968). The origin of genetic code. J. Mol. Biol..

[28] Crick F.H.C. (1970). Central dogma in molecular biology. Nature.

[29] Walker S.I., Davies P.C.W. (2012). The algorithmic origins of life. J. R. Soc. Interface.

[30] Küppers B.O. (1990). Information and the Origin of Life.

[31] Rosen R. (1988). Complexity and information. J. Comput. Appl. Math..

[32] Von Neumann J., Burks A.W. (1966). Theory of Self-Reproducing Automata.

[33] Reenskaug T. (1979). MODELS-VIEWS-CONTROLLERS. http://heim.ifi.uio.no/~trygver/mvc/index.html.

[34] Cech T.R. (1986). RNA as an enzyme. Sci. Am..

[35] Cech T.R. (2009). Crawling out of the RNA world. Cell.

[36] Gilbert W. (1986). The RNA world. Nature.

[37] Orgel L.E. (1994). The origin of life. Sci. Am..

[38] Robertson M.P., Joyce G.F. (2012). The origins of the RNA world. Cold Spring Harb. Perspect. Biol..

[39] Cech T.R. (2000). The ribosome is a ribozyme. Science.

[40] Shapiro R. (2007). A simpler origin of life. Sci. Am..

[41] De Duve C. (1995). The beginnings of life on earth. Am. Sci..

[42] Wachterhäuser G. (1993). The cradle of chemistry of life: On the origin of natural products in a pyrite-pulled chemoautotrophic origin of life. Pure Appl. Chem..

[43] Damer B., Deamer D.W. (2015). Coupled phases and combinatorial selection in fluctuating hydrothermal pools: A scenario to guide experimental approaches to the origin of cellular life. Life.

[44] Lan P., Tan M., Zhang Y., Niu S., Chen J., Shi S., Qiu S., Peng X., Cai G., Cehng H. (2018). Structural insight into precursor tRNA processing by yeast ribonuclease P. Science.

[45] Dyson F. (2004). Origins of Life.

[46] Maynard Smith J., Szathmary E. (1999). The Origins of Life.

[47] Kampfner R.R. (1989). Biological information processing: The use of information for the support of function. Biosystems.

[48] Moreno A., Ruiz-Mirazo K., Terzis G., Arp G.R. (2011). The Informational nature of biological causality. Information and Living Systems: Philosophical and Scientific Perspectives.

[49] Shanks N., Pyles R.A., Terzis G., Arp G.R. (2011). Problem solving in the life cycles of multicellular organisms: Immunology and Cancer. Information and Living Systems: Philosophical and Scientific Perspectives.

[50] Miller W.B. (2018). Biological information systems: Evolution as cognition-based information management. Prog. Biophys. Mol. Biol..

[51] Zwass V. (2016). Information System. https://www.britannica.com/topic/information-system.

[52] Turing A.M. (1937). On computable numbers, with an application to the Entscheidungsproblem: A correction. Proc. Lond. Math. Soc..

[53] Turing A.M. (1952). The chemical basis of morphogenesis. Phil. Trans. R. Soc. Lond..

[54] Shanon C.E. (1948). A mathematical theory of communication. Bell Syst. Tech. J..

[55] Davies P. (1999). The Fifth Miracle.

[56] Biro J.H. (2011). Biological information—Definitions from a biological perspective. Information.

[57] Wiener N. (1965). Cybernetics, Second Edition: Or the Control and Communication in the Animal and the Machine.

[58] Von Bertalanffy L. (1968). General System Theory: Foundations, Development, Applications.

[59] Adriaans P., Zalta E.N. (2012). Information. The Stanford Encyclopedia of Philosophy.

[60] Buckland M. (1991). Information as a thing. J. Am. Soc. Inf. Sci..

[61] Floridi L. (2006). The logic of being informed. Log. Anal..

[62] Price J.R. (1980). An Introduction to Information Theory: Symbols, Signals, and Noise.

[63] Tlusty T. (2007). A model for the emergence of the genetic code as a transition in the noisy information channel. J. Theor. Biol..

[64] Woese C. (1973). Evolution of the genetic code. Naturwissen.

[65] Osawa S. (1995). Evolution of the Genetic Code.

[66] Woese C.R. (1967). The Genetic Code: The Molecular Basis for Genetic Expression.

[67] Koonin E.V. (2015). Why the central dogma: On the nature of biological exclusion principle. Biol. Dir..

[68] De Duve C. (2005). Singularities: Landmarks on the Pathways of Life.

[69] Wong J.T.F. (1975). A co-evolution theory of the genetic code. Proc. Nat. Acad. Sci. USA.

[70] Bada J.L. (2013). New insights into prebiotic chemistry from Stanley Miller’s spark discharge experiments. Chem. Soc. Rev..

[71] Chen I.A. (2015). Prebiotic chemistry: Replicating towards complexity. Nat. Chem..

[72] Wolf Y.I., Koonin E.V. (2007). On the origin of the translation system and the genetic code world by means of natural selection, exaptation, and subfunctionalization. Biol. Dir..

[73] Svoboda P., Cara A. (2006). Hairpin RNA: A secondary structure of primary importance. Cell. Mol. Life Sci..

[74] Eigen M., Winkler-Oswatitsch R. (1981). Transfer-RNA, an early gene. Naturwissen.

[75] Woese C.R. (1965). On the evolution of the genetic code. Proc. Nat. Acad. Sci. USA.

[76] Di Giulio M. (2004). The origin of tRNA molecule: Implications for the origin of protein synthesis. J. Theor. Biol..

[77] Freeman S. (2005). Biological Science.

[78] Di Giulio M. (1992). On the origin of transfer RNA molecule. J. Theor. Biol..

[79] Di Giulio M. (1995). Was it an ancient gene codifying for a hairpin RNA that, by means of direct duplication, gave rise to the primitive tRNA molecule?. J. Theor. Biol..

[80] Tamura K. (2015). Origins and early evolution of the tRNA molecule. Life.

[81] Tanaka T., Kikuchi Y. (2001). Origin of cloverleaf shape of transfer RNA-the double hairpin model: Implication for the role of tRNA intro and long extra loop. Viva Orig..

[82] Widmann J., Di Giulio M., Yarus M., Knight R. (2005). tRNA creation by hairpin duplication. J. Morphol. Evol..

[83] Nagaswamy U., Fox G.F. (2003). RNA ligation and the origin of tRNA. Orig. Life Evol. Biosph..

[84] Lahav N. (1999). Biogenesis: Theories of Life’s Origins.

[85] Zhou R., Basu K., Hartman H., Matocha C.J., Sears S.K., Vali H., Guzman M.I. (2017). Catalyzed synthesis of zinc clays by prebiotic central metabolites. Sci. Rep..

[86] Wong J.T.F. (1981). Coevolution of genetic code and amino acid biosynthesis. Trends Biochem. Sci..

[87] Ikehara K. (2002). Origins of gene, genetic code, protein and life: Comprehensive view of life systems from a GNC-SNS primitive code hypothesis. J. Biosci..

[88] Di Giulio M. (2008). An extension of the coevolution theory of the origin of the genetic code. Biol. Dir..

[89] Noller H.F. (2004). The driving force for molecular evolution of translation. RNA.

[90] Noller H.F. (2012). Evolution of protein synthesis from an RNA world. Cold Spring Harb. Perspect. Biol..

[91] Ramakrishnan V. (2010). The ribosome: Some hard facts about its structure and hot air about its evolution. Cold Spring Harb. Symp. Quant. Biol..

[92] Altstein A.D. (2015). The progene hypothesis: The nucleoprotein world and how life began. Biol. Dir..

[93] Ban N., Nissen P., Hansen J., Moore P.B., Steitz T.A. (2000). The complete atomic structure of the large ribosomal unit at 2.4 A resolution. Science.

[94] Noller H.F. (1993). On the origin of ribosome: Coevolution of subdomains of tRNA and rRNA. The RNA World.

[95] Schimmel P., Alexander R.W. (1998). All you need is RNA. Science.

[96] Nissen P., Hansen J., Ban N., Moore P.B., Steitz T.A. (2000). The structural basis of ribosome activity in peptide bond synthesis. Science.

[97] Orelle C., Carlson E.D., Szal T., Florin T., Jewett M.C., Mankin A.S. (2015). Protein synthesis by ribosomes with tethered subunits. Nature.

[98] Savir Y., Tlusty T. (2013). The ribosome as an optimal decoder: A lesson in molecular recognition. Cell.

[99] Alberts B., Johnson A., Lewis J., Raff M., Roberts K., Walter P. (2002). Molecular Biology of the Cell.

[100] Koonin E.V., Novozhilov A.S. (2008). Origin and evolution of the genetic code: The universal enigma. Life.

[101] Barbieri M. (2016). What is information?. Phil. Trans. R. Soc..

[102] Woese C.R. (2002). On the evolution of cells. Proc. Nat. Acad. Sci. USA.

[103] Dunhill P. (1966). Triplet nucleotide-amino acid pairing: A stereochemical division between protein and non-protein amino acids. Nature.

[104] Yarus M., Widmann J.J., Knight R. (2009). RNA-amino acid binding: A stereochemical era for the genetic code. J. Mol. Evol..

[105] Wong J.T.F. (2014). Emergence of life: From functional RNA selection to natural selection and beyond. Front. Biosci..

[106] Freeland S.J., Knight R.D., Landweber L.F., Hurst L.D. (2000). Early fixation of an optimal genetic code. Mol. Biol. Evol..

[107] Szathmary E. (1999). The origin of the genetic code: Amino acids as cofactors in an RNA world. Trends Genet..

[108] Rodin A.S., Szathmary E., Rodin S.N. (2011). On the origin of genetic code and tRNA before translation. Biol. Dir..

[109] Johnson D.B.F., Wang L. (2010). Imprints of the genetic code in the ribosome. Proc. Nat. Acad. Sci. USA.

[110] Ling J., Reynolds N., Ibba M. (2009). Aminoacyl-tRNA synthesis and translation quality control. Ann. Rev. Microbiol..

[111] Giege R., Sissler M., Florentz C. (1998). Universal rules and idiosyncratic features in tRNA identity. Nucleic Acid Res..

[112] Poole A.M., Jeffares D.C., Penny D. (1998). The path from the RNA worlds. J. Mol. Evol..

[113] Yarus M. (2000). RNA-ligand chemistry: A testable source of genetic code. RNA.

[114] Wong J.T.F., Ng S.K., Mat W.K., Hu T., Xue H. (2016). Coevolution theory of the genetic code at age forty: Pathway to translation and synthetic life. Life.

[115] Eigen M., Schuster P. (1997). The hypercycle: A principle of natural self-organization. Part A: Emergence of the hypercycle. Naturwissens.

[116] Lahav N. (2001). Prebiotic co-evolution of self-replication and translation in RNA world?. J. Theor. Biol..

[117] Copley S.D., Smith E., Morowitz H.J. (2005). A mechanism for the association of amino acids with their codons and the origin of genetic code. Proc. Nat. Acad. Sci. USA.

[118] Crick F.H.C. (1966). Codon-anticodon pairing: The wobble hypothesis. J. Mol. Biol..

[119] Knight R.D., Landweber L.F. (1998). Rhyme or reason: RNA-arginine interactions and the genetic code. Chem. Biol..

[120] Hartwell L.H., Hopfield J.J., Leibler S., Murray A.W. (1991). From molecular to modular cell biology. Nature.

[121] Dagley M.J., Lithgow T., Dalbey R.E., Koehler C.M., Tamanoi F. (2007). TOM and SAM machineries in mitochondrial import and outer membrane biogenesis. The Enzymes.

[122] Goodsell D.S. (2010). The Machinery of Life.

[123] Yockey H.P. (2005). Information Theory, Evolution, and the Origin of Life.

[124] Baum D.A., Lehman N. (2017). Life’s late digital revolution and why it matters for the study of the origins of life. Life.

[125] Reenskaug T. (2003). The Model-View-Controller (MVC) Its Past and Present. http://heim.ifi.uio.no/~trygver/2003/javazone-jaoo/MVC_pattern.pdf.

[126] Pesavento U. (1995). An Implementation of von Neumann’s Self-Reproducing Machine. Artif. Life.

[127] Arenas M. (2013). Computer programs and methodologies for the simulation of DNA sequence data with recombination. Front. Genet..

